# Improving the odds of drug development success through human genomics: modelling study

**DOI:** 10.1038/s41598-019-54849-w

**Published:** 2019-12-11

**Authors:** Aroon D. Hingorani, Valerie Kuan, Chris Finan, Felix A. Kruger, Anna Gaulton, Sandesh Chopade, Reecha Sofat, Raymond J. MacAllister, John P. Overington, Harry Hemingway, Spiros Denaxas, David Prieto, Juan Pablo Casas

**Affiliations:** 1 Institute of Cardiovascular Science, University College London, London, UK; 2 Health Data Research UK and UCL BHF Research Accelerator, London, UK; 3 Benevolent AI, London, UK; 4 European Molecular Biology Laboratory, European Bioinformatics Institute (EMBL-EBI), Wellcome Genome Campus, Cambridge, UK; 5 Institute of Health Informatics, University College London, London, UK; 6 Dorset County Hospital NHS Foundation Trust, Dorchester, UK; 7 Medicines Discovery Catapult, Mereside, Alderley Park, Alderley Edge, Cheshire, UK; 8 Massachusetts Veterans Epidemiology Research and Information Center (MAVERIC), Veterans Administration, Boston, MA USA; 9 Applied Statistics in Medical Research Group, Catholic University of Murcia (UCAM), Murcia, Spain

**Keywords:** Target identification, Medical research, Drug development

## Abstract

Lack of efficacy in the intended disease indication is the major cause of clinical phase drug development failure. Explanations could include the poor external validity of pre-clinical (cell, tissue, and animal) models of human disease and the high false discovery rate (*FDR*) in preclinical science. *FDR* is related to the proportion of true relationships available for discovery (*γ*), and the type 1 (false-positive) and type 2 (false negative) error rates of the experiments designed to uncover them. We estimated the *FDR* in preclinical science, its effect on drug development success rates, and improvements expected from use of human genomics rather than preclinical studies as the primary source of evidence for drug target identification. Calculations were based on a sample space defined by all human diseases – the ‘disease-ome’ – represented as columns; and all protein coding genes – ‘the protein-coding genome’– represented as rows, producing a matrix of unique gene- (or protein-) disease pairings. We parameterised the space based on 10,000 diseases, 20,000 protein-coding genes, 100 causal genes per disease and 4000 genes encoding druggable targets, examining the effect of varying the parameters and a range of underlying assumptions, on the inferences drawn. We estimated *γ*, defined mathematical relationships between preclinical *FDR* and drug development success rates, and estimated improvements in success rates based on human genomics (rather than orthodox preclinical studies). Around one in every 200 protein-disease pairings was estimated to be causal (*γ* = 0.005) giving an *FDR* in preclinical research of 92.6%, which likely makes a major contribution to the reported drug development failure rate of 96%. Observed success rate was only slightly greater than expected for a random pick from the sample space. Values for *γ* back-calculated from reported preclinical and clinical drug development success rates were also close to the *a priori* estimates. Substituting genome wide (or druggable genome wide) association studies for preclinical studies as the major information source for drug target identification was estimated to reverse the probability of late stage failure because of the more stringent type 1 error rate employed and the ability to interrogate every potential druggable target in the same experiment. Genetic studies conducted at much larger scale, with greater resolution of disease end-points, e.g. by connecting genomics and electronic health record data within healthcare systems has the potential to produce radical improvement in drug development success rate.

## Introduction

Almost all small molecule drugs and bio-therapeutics (such as monoclonal antibodies) act by perturbing the function of proteins. Drug development is therefore predicated on identifying those proteins or ‘targets’ that both play a causal role in a disease and are also ‘druggable’, i.e. amenable to pharmacological action by small molecule compounds, peptides or monoclonal antibody therapeutics. The ensuing challenges are to develop compounds specific for the target, with favourable pharmacokinetics and an acceptable toxicity profile, to prove target engagement, and to demonstrate clinical efficacy and safety in humans (Supplementary Note 1).

The extent of these challenges is revealed in an overall failure rate in drug development of over 96%, including a 90% failure rate during clinical development^1–6^. Failure rates are highest for drugs with a new mechanism of action against a previously ‘undrugged’ protein, and for diseases (e.g. Alzheimer’s disease) where the pathogenesis is poorly understood.

Consequences of expensive drug development failures for Pharma have included site closures, job losses, and pruned R&D budgets. Failed R&D also inflates the price of the few successful drugs that trickle through development programmes, which are priced so as to recoup the incurred cost of historical failures and provide shareholders with a return on their investment^7^. This cost is borne initially by healthcare providers but then transferred to citizens through health insurance premiums or taxation.

High failure rates also discourage real innovation in favour of derivative compounds with identical mechanisms of action to existing drugs (‘me too drugs’), minor formulation changes, or drug combinations, which all enjoy the same level of patent protection as drugs with a truly innovative mechanism of action, where the development risk is greater^8^. The result is that some diseases have few, if any, effective therapies, whilst others have a surplus of similar medicines jockeying for a market share. However, since healthcare providers are increasingly sophisticated in their assessment of the value of new medicines, derivative drugs with marginal benefits are now less likely to be taken up by healthcare systems than they once were^9^.

Governments, who are conflicted in their need to ensure cost-efficient healthcare on the one hand, but to support the pharmaceutical sector as a major employer and taxpayer on the other, has explored schemes to reduce barriers to market access for selected drugs^10–12^, but such schemes do not address the root of the drug development problem.

These issues suggest the need for a fresh approach that directly addresses the reasons for high rates of drug development failure^13–15^.

Superseding poor pharmacokinetics and toxicity, lack of efficacy in the intended indication has recently emerged as the major reason for late stage drug development failure, usually established in a randomised controlled clinical trial (RCT), the final step in the drug development pipeline^16–21^. A failure of this type is effectively an expensive demonstration that the target plays no role in the disease.

The reason for the high rate of late stage failure from lack of efficacy can be traced to two system flaws:

1. Preclinical experiments in isolated systems (cells, tissue preparations, isolated organs) together with animal disease models, which are used for the identification and validation of drug targets to progress into clinical phase testing, turn out to be poorly predictive of human efficacy
2. The pivotal clinical experiment, the RCT, is the final step in the drug development pipeline, which means that risk accumulates as a development programme progresses inflating the cost of any failure

The poor predictive ability of preclinical studies for human efficacy (an aspect of the so-called ‘reproducibility crisis’ in laboratory science) can be attributed in part to correctable flaws in experimental design including infrequent use of randomisation and blinding^22–25^.

However, errors of statistical inference leading to a high false discovery (*FDR*) rate may be equally important.

It can be shown (Supplementary Note 2 and Table 1) that

1 $$FDR=\frac{\alpha (1-\gamma )}{(1-\beta )\,\gamma +\alpha \,(1-\gamma )}$$

where:

**Table 1:** The relationship between *α*, *β* and*γ*, the true discovery rate (*TDR*) and the false discovery rate (*FDR*).

Outcome	Causal pairings	Non-causal pairings	Hypotheses tested	*TDR*	*FDR*
Declared positive	*γ*(1 − *β*)	*α*(1 − *γ*)	[*γ*(1 − *β*)] + [*α*(1 − *γ*)]	$\frac{\gamma (1-\beta )}{\gamma (1-\beta )\,+\,\alpha \,(1-\gamma )}$	$\frac{\,\alpha (1-\gamma )\,}{(1-\beta )\,\gamma \,+\,\alpha \,(1-\gamma )}$
Declared negative	*γβ*	(1 − *α*)(1 − *γ*)	[*γβ*] + [(1 − *α*)(1 − *γ*)]		
	*γ*	1 − *γ*	1		

$$\gamma =\mathrm{proportion}\,\mathrm{of}\,\mathrm{true}\,\mathrm{target} \mbox{-} \mathrm{disease}\,\mathrm{relationships}$$

$$\beta =\mathrm{false} \mbox{-} \mathrm{negative}\,\mathrm{rate}$$

$$1-\beta =\mathrm{power}\,(\mathrm{detection}\,\mathrm{rate}\,\mathrm{for}\,{\rm{a}}\,\mathrm{real}\,\mathrm{effect})$$

$$\alpha =\mathrm{false} \mbox{-} \mathrm{positive}\,\mathrm{rate}$$

*FDR* gives the probability of no causal relationship given success was declared, by applying Bayes rule to the above quantities.

False discoveries likely greatly outnumber true discoveries in preclinical research^26^ because:

- The proportion of true relationships available for discovery (*γ*) is greatly outweighed by the proportion of false ones (1 − *γ*)
- The usual experimental false positive rate (*α*) of 0.05 leads to many false relationships being declared as real^27–32^
- Studies are often too small to reliably detect real relationships because the power(1 − *β*) is often lower than that pre-specified at the study design stage. Over optimistic estimates of effect sizes also means that when true relationships are detected, the effect sizes will be overestimated^30^

The result is that seemingly promising but flawed target-disease indication hypotheses are liable to progress from preclinical into clinical phase development only to stumble expensively at phase 2 or 3 for lack of efficacy.

The high *FDR* in standard preclinical research could be reduced by routinely setting more stringent values for (1 − *β*) and *α*^32^. However, there is a penalty to pay in the requirement for larger sample sizes (Supplementary Note 2). This is outwardly at odds with the 3R principles that encourage reduction in the number of animals sacrificed in medical research. However, ultimately, a smaller number of larger but definitive preclinical experiments may utilise fewer animals than numerous small, equivocal experiments undertaken in pursuit of an eventually futile hypothesis.

Nevertheless, other aspects of preclinical experimentation are unalterable: the proportion of true relationships available for discovery (*γ*) is fixed; experiments in isolated systems will never be fully representative of the situation in the whole animal; nor will animal models of human disease ever be completely reliable predictors of human success. A different solution is needed to address these limitations.

Relationships between variation in the genome and normal development and behaviour, physiology, metabolism, and disease susceptibility, (collectively, the phenotype), have been progressively uncovered in the last two decades. This has been enabled, in large part, by a single research design – the genome wide association study (GWAS). But the GWAS design is also beginning to reveal its potential as a new resource for drug development. GWAS have ‘rediscovered’ the known treatment indication or mechanism-based adverse for around 70 of the 670 known targets of licensed drugs^33^. This observation suggests that new drug targets for diseases with few effective therapies could also be identified using the same approach. Retrospective analyses have shown that the probability of a gene being associated with a human disease given that it encodes an approved drug target is greater than expected by chance^34^. Studies using variants in genes encoding individual targets have accurately predicted success or failure in RCTs^35,36^, helped separate mechanism-based from off-target actions of new drugs^37,38^, and identified new treatment indications and repurposing opportunities for established drugs^39^ (Supplementary Information). Genetic prediction of pharmacological action has been shown to encompass both small molecule drugs and bio therapeutics, on proteomics and metabolomics^40^, as well as physiological biomarkers and disease end-points. Collectively, these examples illustrate the potential of genetics and genomics to address the nub of the drug development problem: matching the right drug target with the right disease through GWAS (target identification); and delineating the diverse impacts of perturbing an individual target on a wide range of outcomes (target validation).

GWAS overcome many of the design flaws inherent in standard preclinical testing in isolated cells, tissues and animal models. They are an experiment in the correct organism (the human); have the lowest false discovery rate in any field of biomedicine (Supplementary Note 3); provide the systematic, concurrent interrogation of every potential drug target on the condition of interest (rather than a few targets selected from a larger pool); and exploit the unique attributes of genetic variation (fixed and allocated at random), which mimics the design of the pivotal experiment in drug development, the RCT^41–44^.

Studies that exploit the naturally randomised allocation of genetic variants that instrument an exposure of interest for causal inference have been termed Mendelian randomisation studies. Where the exposure of interest is the protein encoded by a specific gene and this is a drug target, the paradigm has been referred to as Mendelian randomisation for drug target validation (see Supplementary Information, Ref 1), since it was inspired by, and represents a special case of the Mendelian randomisation paradigm, which was applied initially to help determine the causal relevance of environmental exposures or disease related biomarkers^45^. A GWAS study can be considered to be a type of Mendelian randomisation analysis for drug target validation where variants in *every gene* encoding a drug target are interrogated for their association with a disease at the same time. This is made possible because naturally occurring variants in or around a gene (whether common or rare, coding or non-coding) are ubiquitous in the genome. Those that influence expression or activity of the encoded protein can, through their associations with biomarkers and disease end-points, anticipate the effect of pharmacological action on the same protein where this is druggable. Such an approach is disease agnostic, though it may be unsuited to aspects of cancer drug development, where somatic rather than germ line mutations perturb the targets of interest, or to the development of anti-infective drugs, in cases where the therapeutic drug target is in the pathogen rather than the human host.

In this paper, we develop a new conceptual framework and apply simple probabilistic reasoning to (a) explain why failure and inefficiency in orthodox preclinical drug development is the norm, and success the exception; and (b) estimate the probability of development success given the gene encoding the drug target is associated with the corresponding disease.

## Methods

**Figure 1:**
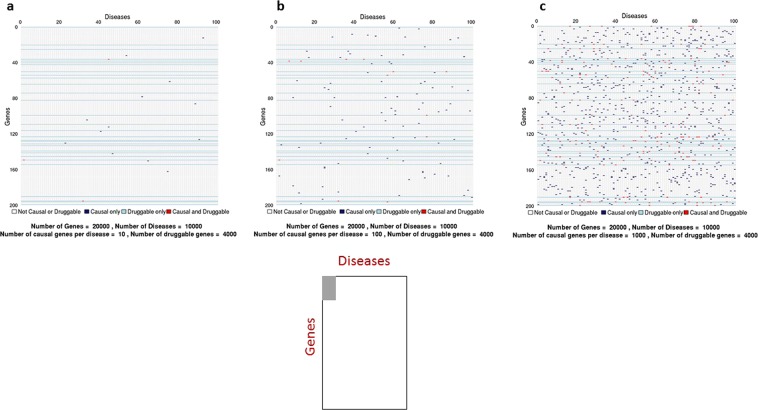
Sample space (*N*_*G*_ × *N*_*T*_) defined by 10,000 human diseases (columns) and 20,000 protein coding genes (rows). Expanded region comprising 1/10,000tℎ of the whole sample space is enlarged: (**a)** based on 10*th* causative genes per disease); (**b)** (based on 100 causative genes per disease); and **c** (based on 1000 causative genes per disease). Each cell represents a unique gene-disease pairing. Dark blue cells indicate causal gene-disease pairings, light blue cells druggable gene-disease pairings, with red cells indicating causal and druggable gene disease pairings.

Since drug development depends on identifying proteins that play a causal role in a disease of interest, we introduce the concept of a sample space spanned by all human diseases – the ‘disease-ome’ – represented as columns; and all protein coding genes – ‘the protein coding genome’– represented as rows. The result is a matrix of unique gene- (or equivalently protein-) disease pairings (Fig. 1).

We focus on common (multifactorial) human diseases of potential therapeutic interest that have both genetic and environmental contribution (Supplementary Note 4). We assume subsets of all the proteins encoded in the genome (Supplementary Note 5**)** play a causal role in any disease (Supplementary Note 6), and that only certain proteins are amenable to targeting by small molecule drugs or bio-therapeutics, leading to the concept of the ‘druggable genome: the set of genes encoding actual or potential targets of drugs (Supplementary Note 7).

We therefore establish some definitions.

$$\{G\}\,{\rm{is}}\,{\rm{the}}\,{\rm{set}}\,{\rm{of}}\,{\rm{protein}}-{\rm{coding}}\,{\rm{genes}}$$

$$\{D\}\,{\rm{is}}\,{\rm{the}}\,{\rm{set}}\,{\rm{of}}\,{\rm{common}}\,{\rm{human}}\,{\rm{diseases}}$$

$$\{GD\}\,{\rm{is}}\,{\rm{the}}\,{\rm{set}}\,{\rm{of}}\,{\rm{all}}\,{\rm{possible}}\,{\rm{gene}}\,({\rm{or}}\,{\rm{protein}})-{\rm{disease}}\,{\rm{pairs}}$$

$$\{C\}\,{\rm{is}}\,{\rm{the}}\,{\rm{set}}\,{\rm{of}}\,{\rm{causal}}\,{\rm{genes}}\,{\rm{for}}\,{\rm{a}}\,{\rm{given}}\,{\rm{disease}}$$

$$\{CD\}\,{\rm{is}}\,{\rm{the}}\,{\rm{set}}\,{\rm{of}}\,{\rm{all}}\,{\rm{causal}}\,{\rm{gene}}-{\rm{disease}}\,{\rm{pairs}}$$

{*T*}isthesetofgenesencodingdruggabletargets: the druggable genome

**Figure 2:**
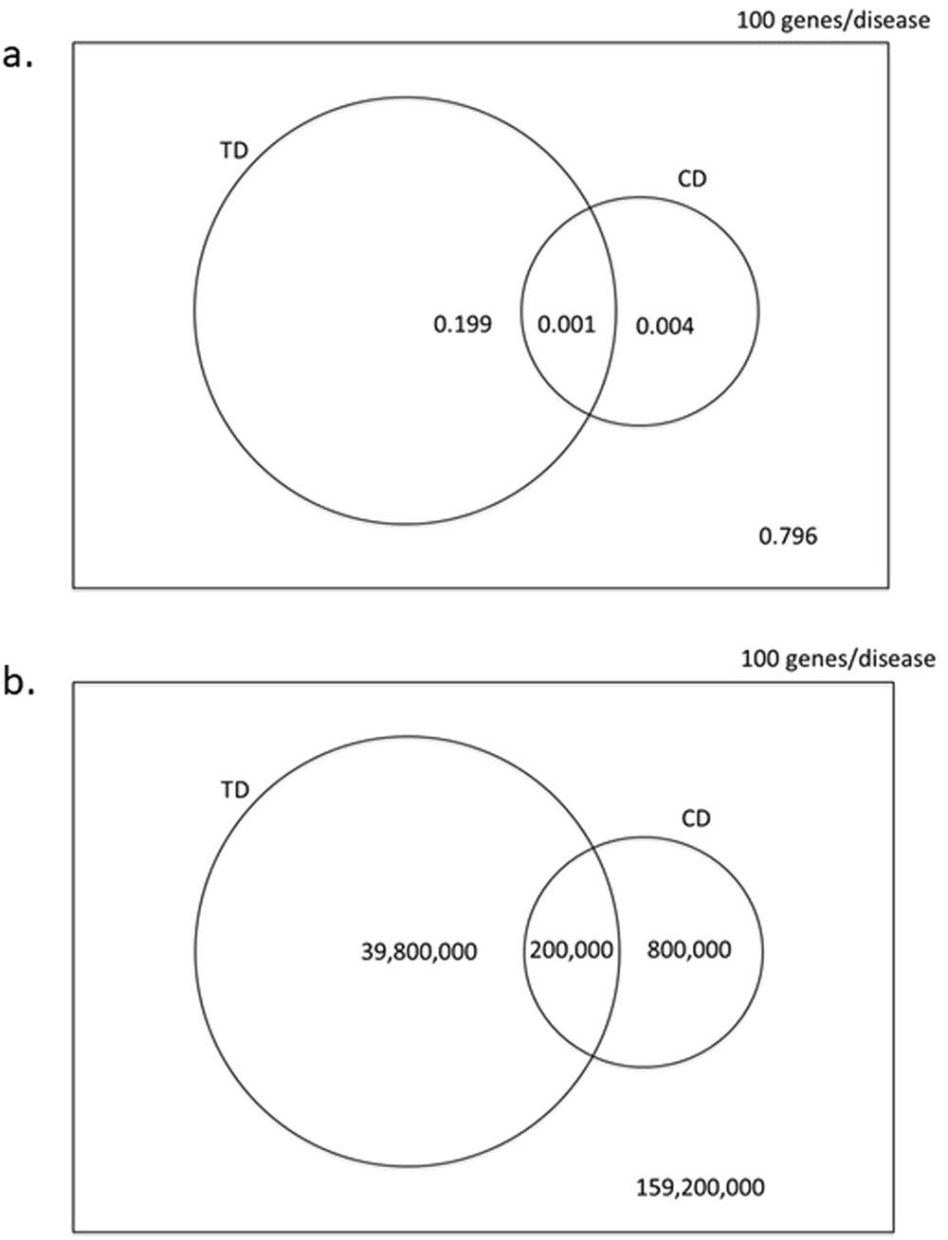
Venn diagram illustrating the (**a)** the probabilities of selecting and (**b**) the number of causal, druggable gene-disease pair ($CD\cap TD$), a druggable gene disease pair (*TD*) and a causal, gene disease pair (*CD*) from 200 × 10^6^ gene disease pairings, 100 causal genes per disease and 4000 druggable genes from the 20,000 in the genome. (Not to scale).

Based on arguments rehearsed in Supplementary Notes 4–7 (see also Table S1 and Fig. 2**)**, we set the following parameters:

$${N}_{G}=\,{\rm{Total}}\,{\rm{number}}\,{\rm{of}}\,{\rm{protein}}-{\rm{coding}}\,{\rm{genes}}=20,\,000$$

$${N}_{D}\,={\rm{Total}}\,{\rm{number}}\,{\rm{of}}\,{\rm{complex}}\,{\rm{human}}\,{\rm{diseases}}=10,\,000$$

$${N}_{GD}={\rm{Total}}\,{\rm{number}}\,{\rm{of}}\,{\rm{gene}}-{\rm{disease}}\,{\rm{pairs}}=10,\,000\times 20,\,000=200\times {10}^{6}$$

$$C={\rm{the}}\,{\rm{number}}\,{\rm{of}}\,{\rm{causal}}\,{\rm{genes}}\,{\rm{in}}\,{\rm{a}}\,{\rm{given}}\,{\rm{disease}}$$

$$\bar{C}={\rm{the}}\,{\rm{average}}\,{\rm{number}}\,{\rm{of}}\,{\rm{causal}}\,{\rm{genes}}\,{\rm{per}}\,{\rm{disease}}=100$$

$${N}_{CD}={\rm{Total}}\,{\rm{number}}\,{\rm{of}}\,{\rm{causal}}\,{\rm{gene}}-{\rm{disease}}\,{\rm{pairs}}=100\times 10,\,000=1\times {10}^{6}$$

$${N}_{T}={\rm{Total}}\,{\rm{number}}\,{\rm{of}}\,{\rm{genes}}\,{\rm{encoding}}\,{\rm{druggable}}\,{\rm{targets}}=4000$$

We next formalise assumptions on which we base the subsequent calculations. Although some of the assumptions are oversimplifications, and exceptions can be identified from current drugs and diseases, they help to estimate certain ‘base-case’ probabilities. In Supplementary Note 8, we dissect these parameters and assumptions, and explore the impact of any modifications on our estimates.

**Assumption 1**: Each gene encodes a unique protein with a single function

**Assumption 2**: A given protein can influence the risk of more than one disease

**Assumption 3**: The probability of a protein influencing the pathogenesis of one disease is independent of the probability that it influences any other

**Assumption 4**: Drug treatments for human disease target proteins encoded in the germ line (We exclude drug targets encoded by the abnormal genome of cancer cells as well as antimicrobials, which typically target proteins encoded in the genomes of pathogens. For further discussion, see Supplementary Note 8**)**.

**Assumption 5**: The probability that a protein affects disease pathogenesis and the probability the protein can be targeted by a drug is independent

**Assumption 6**: Inaccurate target selection is the exclusive reason for clinical phase drug development failure

**Assumption 7:** DNA sequence variants in and around a gene encoding a drug target that alter expression or activity of the encoded protein (*cis*-acting variants), are ubiquitous in the genome

**Assumption 8:** The association of *cis-*acting variants with biomarkers and disease end-points in a population genetic study accurately predict the effects of pharmacological modification of the encoded target in a clinical trial

**Assumption 9:** Genotyping arrays used in GWAS provide comprehensive, appropriately powered coverage of the genome, and associations discovered at any one gene are independent of those detected at any other gene

We use simple frequencies, binomial or hypergeometric distributions, and 2 × 2 tables to calculate a range of metrics relevant to drug development success, and to compare target identification based on standard preclinical models with target identification through GWAS.

## Results

### Part A. Target identification through orthodox preclinical development

#### False discovery rate in preclinical science and drug development success rate

Ioannidis^27^ and others have provided empirical evidence from many research fields of extremely high rates of false discovery, leading to pervasive unreliability of the evidence base used to inform drug development^46^. In Bayesian terms, the prior probability of correctly pairing a causal gene (or protein) with a disease may be close to that of the background probability of a success in a *random pick* from the sample space.

Let us assume as a start point that this is the case. Then, using assumptions 1–3, the probability (*P*_*c*_) that any gene- (or, equivalently, any protein)-disease pairing selected at random from the set of all possible gene-disease pairs {*GD*} also belongs to the set of causal gene-disease pairs {*CD*} is given by:

2 $${P}_{C}=\frac{{N}_{CD}}{{N}_{GD}}$$

Or;

3 $${P}_{C}=\frac{\bar{C}}{{N}_{G}}$$

Using either equation, and taking $\bar{C}=100$; *P*_*C*_ = 0.005

If $$\bar{C}=1000,{P}_{C}\,$$= 0.05

If $$\bar{C}=10,\,{P}_{C}$$= 0.0005

As follows from Eq. 3, *P*_*c*_ is independent of the number of diseases under consideration, as long as $\bar{C}\,$ is constant.

*P*_*C*_ can also be interpreted as the proportion of causal relationships amongst all possible gene-disease pairings, and can hence be represented as *γ*_*C*_, the proportion of causal protein-disease relationships available for discovery (Supplementary Note 2).

Therefore:

4 $${P}_{C}={\gamma }_{C}$$

If preclinical experiments are initiated based on target-disease pairings drawn at random from the sample space, where $\bar{C}=100$; *γ*_*C*_ = 0.005; *α* = 0.05; and (1 − *β*) = 0.8, then using Eq. 1,

$$FDR=\frac{\alpha (1-\gamma )}{(1-\beta )\,\gamma +\alpha \,(1-\gamma )}=92.6 \% $$

This *FDR* estimate is very close to that made previously by Ioannidis^26^ and also close the observed rate of drug development failure. We return to this point in a later section.

#### A priori probability of accurate drug target identification

Only a portion of the genome encodes proteins readily accessible to small molecule drugs, monoclonal antibodies or peptides that currently comprise the major chemical categories of medicines.

The probability(*P*_*T*_) of selecting a druggable gene (protein)-disease pairing at random is given by:

5 $${P}_{T}=\frac{{N}_{T}}{{N}_{G}}$$

$${P}_{T}=\frac{4,000}{20,000}=0.2$$

To estimate the probability *P*_*CT*_ of selecting a disease-causing *and* druggable protein-disease pairing at random from the sample space, we take the probability that a protein affects disease pathogenesis and the probability the protein can be targeted by a drug to be independent (**Assumption 5)**.

Therefore,

6 $${P}_{CT}={P}_{c}\times {P}_{T}$$

$$\begin{array}{l}{P}_{CT}=0.005\times 0.2\\ \,{P}_{CT}=0.001\end{array}$$

Corresponding probabilities and counts for scenarios in which $\bar{C}=100,\,{\rm{and}}\,\bar{C}=1000$ are shown in Figs. S1 and S2 and Table S2. Note that these probabilities are independent of *N*_*D*_, the total number of diseases under consideration.

Following the arguments presented previously (Eq. 4), *P*_*CT*_ can also be interpreted as *γ*_*CT*_, the proportion of causal, druggable gene-disease pairs from the sample set of all gene-disease pairings.

From Eq. 1, with $\bar{C}=100$, *γ*_*CT*_ = 0.001, *α* = 0.05; and (1 − *β*) = 0.8 the *FDR* for druggable and causal protein disease pairings is estimated as 98.4% (Table 1).

However, the probability of more direct interest is that of identifying a druggable, disease-causing gene having already specified the disease of therapeutic interest. Since we assume the probability of a protein influencing the pathogenesis of one disease is independent of the probability that it influences any other **(Assumption 3**) *P*_*C*_, *P*_*T*_ and *P*_*CT*_ are the same for each individual disease, as they are for the sample space overall.

For any given disease, with *C* causal genes, we can therefore write:

7 $$\begin{array}{rcl}{P}_{c} & = & \frac{C}{{N}_{G}}\\ {P}_{T} & = & \frac{{N}_{T}}{{N}_{G}}\\ {P}_{CT} & = & {P}_{c}\times {P}_{T}=(\frac{C}{{N}_{G}})(\frac{{N}_{T}}{{N}_{G}})\end{array}$$

**Figure 3:**
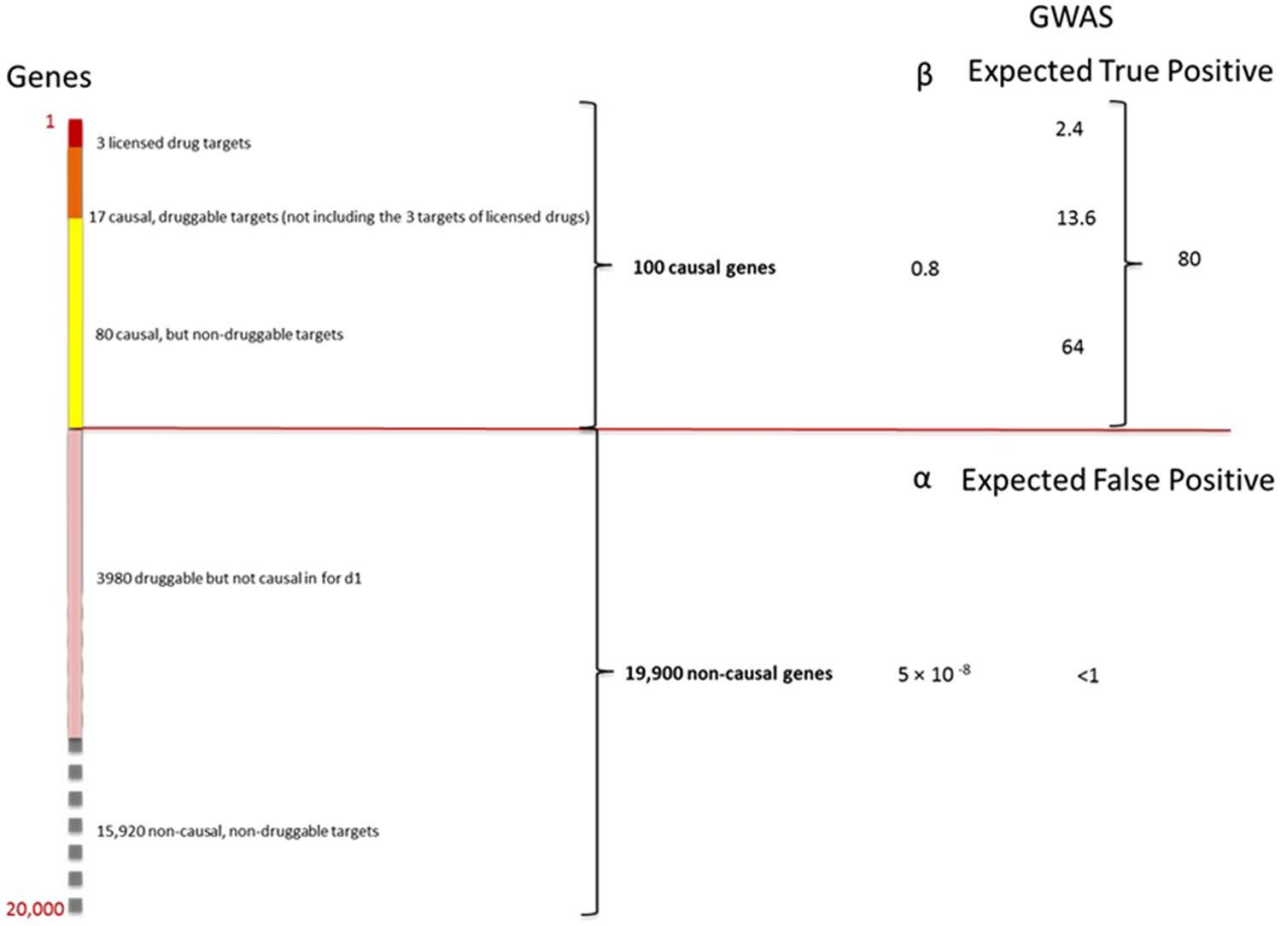
Re-assorted ‘therapeutic genome’ of a hypothetical disease (*d*_1_). The 20,000 protein coding genes are organised into 100 causal and 19,900 non-causal genes. Causal genes are further subdivided into 20 that are also druggable and 80 that are not. Of the 20 causal, druggable genes, 3 are the targets of licensed drugs for the treatment of *d*_1_. Of the non-causal genes, 3980 are druggable but not causal for *d*_1_. The right hand panel indicates the expected number of true and false positive genes (including druggable genes) expected in a GWAS of *d*_1_ undertaken with a sample size that provides power, 1 − *β* = 0.8 and type 1 error rate of *α* = 5 × 10^−8^ at all loci.

These estimates can now be used to re-assort all genes in the genome from a therapeutic perspective for any given disease (Fig. 3).

For example, in a hypothetical disease (*d*_1_), where *C* = 100, the expected number of causal *and* druggable genes is given by:

$${P}_{CT}\times {N}_{G}=(\frac{100}{20,\,000})(\frac{4000}{20,\,000})\times 20,\,000=\,20$$

*C* − 20 = 80 causal genes would therefore be categorized as non-druggable. Of the *N*_*G*_ − *C* = 19,900 non-causal genes, one fifth ($\frac{{N}_{T}}{{N}_{G}}\times 19,900=3980$) would be expected to be druggable but not causal in disease *d*_1_ (though of course some could be causal and of therapeutic interest in a different disease). The remaining 19,900 − 3980 = 15,920 genes would be classified as neither causal for *d*_1_, nor druggable.

Table S2 illustrates the influence of different estimates of *C* on *P*_*C*_(*γ*_*C*_) and *P*_*CT*_(*γ*_*CT*_).

Based on Eqs. 3–7, we can also write

$${\gamma }_{CT}=(\frac{C}{{N}_{G}})(\frac{{N}_{T}}{{N}_{G}}\,)$$

This equation suggests routes by which the *a priori* probability of accurate drug target identification might be increased. *C* is not amenable to manipulation, being largely determined by evolutionary forces; *N*_*G*_ is also fixed; however, *N*_*T*_ could be increased by developing technologies that allow a broader range of gene products to be targeted therapeutically. The development of therapeutic monoclonal antibodies has already increased *N*_*T*_ by permitting targeting of proteins that were not previously amenable to a small molecule therapeutic strategy.

*γ*_*CT*_ could also be increased by constraining the sample space to the druggable genome. We could then write:

$${\gamma }_{CT}=(\frac{C}{{N}_{G}})(\frac{{N}_{T}}{{N}_{T}}\,)=(\frac{C}{{N}_{G}})$$

If *C* = 100, $${\gamma }_{CT}=\frac{100}{20,000}=0.005$$

Thus, the simple expedient of focusing target identification on the 4000 druggable genes, rather than all 20,000 protein-coding genes, increases *γ*_*CT*_ by a factor of five from 0.001 to 0.005: among the set of druggable genes, all causal genes are automatically both causal and druggable.

Alternatively, if it were possible, hypothetically, to reliably remove genes considered to have a low or no probability of playing a causal role in the disease of interest, i.e. focusing on the set {*N*_*C*'_}, where:

$\{{N}_{C^{\prime} }\},=$set of likely to be causal genes in the disease of interest

We could then write:

$${\gamma }_{CT}={P}_{CT}=(\frac{C}{{N}_{{C}^{\text{'}}}})(\frac{{N}_{T}}{{N}_{G}}\,)$$

If it were possible, hypothetically, to reliably remove genes considered to have a low or no probability of playing a causal role in the disease of interest, i.e. focusing on the set of causal genes, then:

$$\mathop{\mathrm{lim}}\limits_{{N}_{C^{\prime} }\to C}\,[(\frac{C}{{N}_{C^{\prime} }})(\frac{{N}_{T}}{{N}_{G}}\,)]\to (\frac{C}{C}\,)(\frac{{N}_{T}}{{N}_{G}}\,)=0.2$$

In the limit, among an exclusively causal set of genes, the probability of being causal and druggable is simply the probability of being druggable (**Assumption 5)**. Eliminating non-causal while retaining causal genes is the crux of the target identification problem. We show later why GWAS (or whole genome or exome sequencing studies) address this issue as an inherent feature of their study design.

#### *A posteriori estimates* of true and false relationships explored in contemporary drug development

If the vast majority of research findings are false^26^, then the proportion of target-disease indication pairings studied in drug development should be close to that from a random pick from all possible target-indication pairs.

To estimate if this is the case, we use reported preclinical and success rates^2,21^ to make *a posteriori* estimates of the proportion of true target-disease relationships explored in preclinical and clinical phase development. We compare these *a posteriori estimates* to the *a priori* estimates based on a random pick of target-disease pairings in the sample space.

To facilitate the calculations, we reduce drug development to a two-stage process: a preclinical component (stage 1), whose function is to predict target-disease pairings destined for clinical phase success, and a clinical component (stage 2), whose function is to evaluate target-disease pairings brought forward from stage 1. Success in stage 2 is thus dependent on the predictive performance of stage 1. Since clinical phase drug development failure due to incorrect target specification accounts for around two in every three late-stage failures^2,21^, we utilize a further simplifying assumption (**Assumption 6)** that inaccurate target selection is the exclusive reason for clinical phase (stage 2) drug development failure.

Key variables in the following section are indexed by the lower-case suffix *pc* to denote preclinical and the lower-case suffix *c* to denote clinical stage development. Possible outcomes from pre-clinical and clinical phase development are summarized Table 2, where:

**Table 2:** The relationship *α*, *β*, and *γ TP*, *TN*, *FP FN*, and the declared success rate (*s*) in preclinical and clinical drug development (see text for details).

		True relationship	No true relationship	All
**Stage 1:** **Preclinical development** ${\boldsymbol{(}}{\boldsymbol{pc}}{\boldsymbol{)}}$	**Declared success**	${{{TP}}_{{pc}}=\gamma }_{{pc}}(1-{\beta }_{{pc}})$	${{{FP}}_{{pc}}=\alpha }_{{pc}}(1-{\gamma }_{{pc}})$	${S}_{{pc}}$
	**Declared failure**	${{{FN}}_{{pc}}=\gamma }_{{pc}}{\beta }_{{pc}}$	${{TN}}_{{pc}}=(1-{\alpha }_{{pc}})(1-{\gamma }_{{pc}})$	$1-{S}_{{pc}}$
	**All**	${\gamma }_{{pc}}$	$1-{\gamma }_{{pc}}$	1
**Stage 2:** **Clinical Development** ${\boldsymbol{(}}{\boldsymbol{c}}{\boldsymbol{)}}$	**Declared success**	${{TP}}_{c}={\gamma }_{c}(1-{\beta }_{c})$	${{{FP}}_{c}=\alpha }_{c}(1-{\gamma }_{c})$	${S}_{c}$
	**Declared failure**	${{FN}}_{c}={\gamma }_{c}{\beta }_{c}$	${{TN}}_{c}=(1-{\alpha }_{c})(1-{\gamma }_{c})$	$1-{S}_{c}$
	**All**	${\gamma }_{c}$ = ${{TDR}}_{{pc}}$	$1-{\gamma }_{c}$	1

$$\gamma ={\rm{proportion}}\,{\rm{of}}\,{\rm{true}}\,\mathrm{target}-\mathrm{disease}\,{\rm{relationships}}$$

*TP* = true positive rate

*FP* = false positive rate

*TN* = true negative rate

*FN* = false negative rate

*S* = declared success rate

1 − *S* = declared failure rate

*TDR* = true discovery rate

If a *clinical* phase drug development programme follows every declared *preclinical* success, the proportion of true target disease relationships in *clinical* phase development is equivalent to the *preclinical* true discovery rate, so we can write:

8 $${\gamma }_{c}=TD{R}_{pc},\,({\rm{where}}\,TD{R}_{pc}=\frac{T{P}_{pc}}{{S}_{pc}})$$

It can be also be shown, by substitution and re-arrangement (Supplementary Note 9) that;

9 $$TD{R}_{c}=\frac{T{P}_{c}}{{S}_{c}}=\frac{TD{R}_{pc}\,(1-{\beta }_{c})}{TD{R}_{pc}(1-{\beta }_{c})+{\alpha }_{c}(1-TD{R}_{pc})}$$

By further substitution and re-arrangement (see Supplementary Note 9):

10 $$TD{R}_{C}=\frac{1}{1+(\frac{{\alpha }_{c}}{1-{\beta }_{c}})(\frac{{\alpha }_{pc}}{1-{\beta }_{pc}})(\frac{1-{\gamma }_{pc}}{{\gamma }_{pc}})}$$

Equation 10 illustrates that the clinical phase true discovery rate can be resolved mathematically into terms that encompass clinical phase power and experimental false positive rate $({\rm{the\; term}}\frac{{\alpha }_{c}}{1-{\beta }_{c}})$, preclinical phase power and experimental false positive rate $({\rm{the\; term}}\frac{{\alpha }_{pc}}{1-{\beta }_{pc}})$, and the true relationships available for discovery $({\rm{the\; term}}\frac{1-{\gamma }_{pc}}{{\gamma }_{pc}})$. In this sense, Eq. 10 can be conceived as a mathematical summary of the probabilities and parameters determining drug development success. Equation 10 expresses *TDR*_*C*_ as the odds of a randomly chosen drug being effective, the Bayes factor provided by a preclinical discovery, and the Bayes factor provided by a clinical discovery.

Using the calculations elaborated in Supplementary Note 9, and based on published ‘success rates’ for preclinical (*S*_*pc*_ = 0.4)^2^ and clinical development (*S*_*c*_ = 0.1)^2,22^ and assuming values of *α* = 0.05 and 1 − *β* = 0.8, in both preclinical and clinical development, we estimate*γ*_*c*_ = 0.0667 and *γ*_*pc*_ = 0.03335; at *α*_*pc*_ = 0.386 and *FDR*_*pc*_ = 0.933.

**Figure 4:**
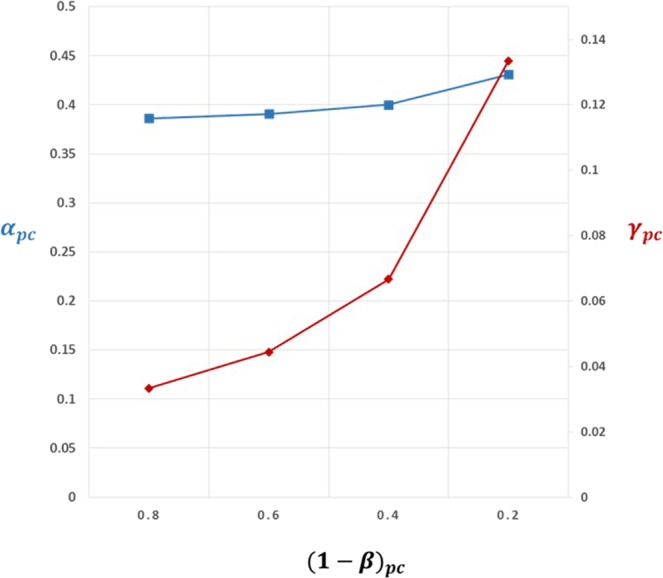
Back calculation of proportion of true target-disease relationships (*γ*_*pc*_) studied in preclinical development, inferred from observed rates of clinical success (*S*_*C*_ = 0.1) and preclinical success (*S*_*pc*_ = 0.4). Estimates of *γ*_*pc*_ assume power in clinical phase development(1 − *β*_*c*_) = 0.8 and false positive rate in clinical development, *α*_*c*_ = 0.05, so that the proportion of true target-disease relationships in clinical development, *γ*_*c*_ = 0.0667. The graph shows estimates of *γ*_*pc*_ (red line) for a range of values for power (1 − *β*_*pc*_) in preclinical development and corresponding estimates of the preclinical false positive rate, *α*_*pc*_ (blue line). (See text for details).

Figure 4 illustrates values of *γ*_*pc*_ and *α*_*pc*_ for a range of values for 1 − *β*_*pc*_ from 0.2 to 0.8,using a fixed value of *γ*_*c*_ = 0.0667. For values of 1 − *β*_*pc*_ in this range, values for *γ*_*pc*_ lie in the range 0.033 to 0.133, representing between a 6.5-fold to 26.5-fold enrichment in the proportion of true relationships actually studied in preclinical drug development over a random pick from a sample space demarcated by all diseases and the druggable genome (*γ*_*pc*_ = 0.005). Although these enrichment rates for established preclinical drug development might appear substantial, this degree of enrichment is insufficient to prevent a large proportion of false target-disease relationships being pursued during clinical phase development. This accounts for the low rates of clinical success. It also raises the possibility that a large proportion of declared clinical successes are actually themselves false discoveries, as illustrated by estimated values of *TDR*_*c*_ (Table 2).

#### Parallel development programmes for a single success

Pursuing multiple drug development programmes in parallel, each pursuing a different target, recognizing that the majority will fail, is a common, though inefficient strategy in contemporary drug development. For example, 1120 unique pipeline drug programmes for Alzheimer’s disease were initiated across the industry in the period 1995–2014^47^.

**Figure 5:**
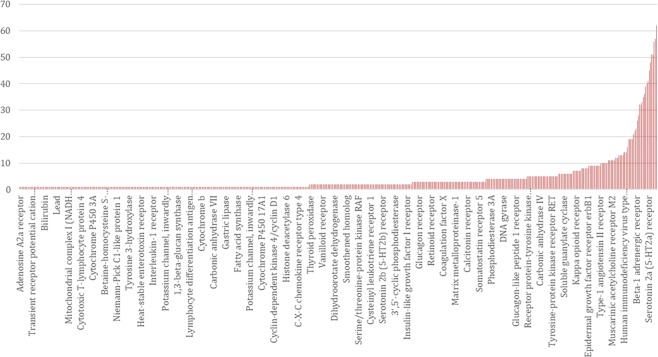
Distribution of number of licensed drug compounds per target.

Around 4 in 100(0.04) preclinical drug development programmes yield licensed drugs. However, this estimate is based on the success rates of compounds rather than targets. The success in early development of a first-in-class molecule for a given disease indication is often followed by a flurry of development programmes, distributed across several companies, based on the same target and disease indication. The consequence is that multiple drugs may emerge, all in the same class. Using the ChEMBL database, we estimate a median of 2 (mean of 4) licensed drugs per efficacy target (Fig. 5). Therefore, the overall developmental success rate for targets could be around half that of compounds i.e. 2 in 100(0.02).

With an overall developmental success rate for targets of 0.02, how many parallel programmes (*N*) should be pursued in order to have a 90% chance of at least one success?

Assuming all programmes are independent, the probability of all *N* programmes failing is:

$${(1-{P}_{s})}^{N}$$

where *P*_*s*_ = with in programme success rate

A 90% probability of at least 1 success equates to a 10% probability of no success in any programme (i.e. a 10% probability of all programmes failing). Therefore:

$${(1-{P}_{s})}^{N}=0.1$$

If *P*_*s*_ = 0.02

$$N=\frac{\log \,0.1}{\log \,(1-0.02)}=114$$

Thus, 114 parallel, independent programmes, should be pursued on average, to have a 90% probability of at least one developmental success; 34 programmes to have an 50% (evens) chance of at least one success. Values of *N* for a range of hypothetical values of *P*_*s*_are shown in Table S3.

#### Impact of a target selection step in orthodox preclinical drug development

Logistics and cost preclude orthodox (non-genomic) pre-clinical studies based on cells, tissues and animal models from evaluating the potential causal role of every protein in every disease. This imposes a selection step in drug development in which a subset of targets must first be prioritized for inclusion in preclinical drug development programmes. By contrast, as we elaborate later, a GWAS is capable of interrogating every target in parallel, without a selection step.

This selection step in standard preclinical drug development introduces two constraints. First, it results in slow progress in the investigation of target-disease indication hypotheses. To illustrate, the sample space spanned by the druggable genome and human diseases contains *N*_*T*_ × *N*_*D*_ = 40 × 10^6^ unique druggable gene (or protein target)-disease pairs, of which 0.005 × (40 × 10^6^) = 200,000 would be expected to be causal $({\rm{if}}\,\bar{C}=100)$. A recent survey estimated only 15, 101 unique human target-indication pairings have been studied in drug development programmes over the last two decades, representing just 0.04% of this theoretical sample space^48^.

The second constraint is illustrated by a further probability consideration. The probability that 0, 1, 2, … *A* causal targets occurs in a sample of size *N* (where each member of the sample corresponds to an independent development programme based on a different drug target –disease indication pairing), drawn without replacement from the pool of 4000 druggable genes (proteins), of which *C* are causal for the disease of interest, is given by the hypergeometric distribution where:

$$P(A)=\frac{(\begin{array}{c}C\\ A\end{array})(\begin{array}{c}4,\,000-C\,\\ N-A\end{array})}{(\begin{array}{c}4,\,000\\ N\end{array})}$$

The expected number of causal, druggable targets *E*(*A*) in the sample of development programmes is given by:

$$E(A)=N(\frac{C}{4,\,000}),\,{\rm{with}}\,{\rm{SD}}=\sqrt{\frac{N\,C\,(4,\,000-{\rm{C}})(4,\,000-N)}{4,\,{000}^{2}(4,\,000-1)}}$$

Expected values for *A* based on a range of values of *N* and *C* are shown in Table S3. Four preclinical development outcomes are therefore possible: (a) one or more true positives is correctly identified with no false positives; (b) a mixture of one or more true and false positives emerge; (c) there are no positive findings; or, (d) in a worst-case scenario, one or more false positive results emerge with no true positives.

**Figure 6:**
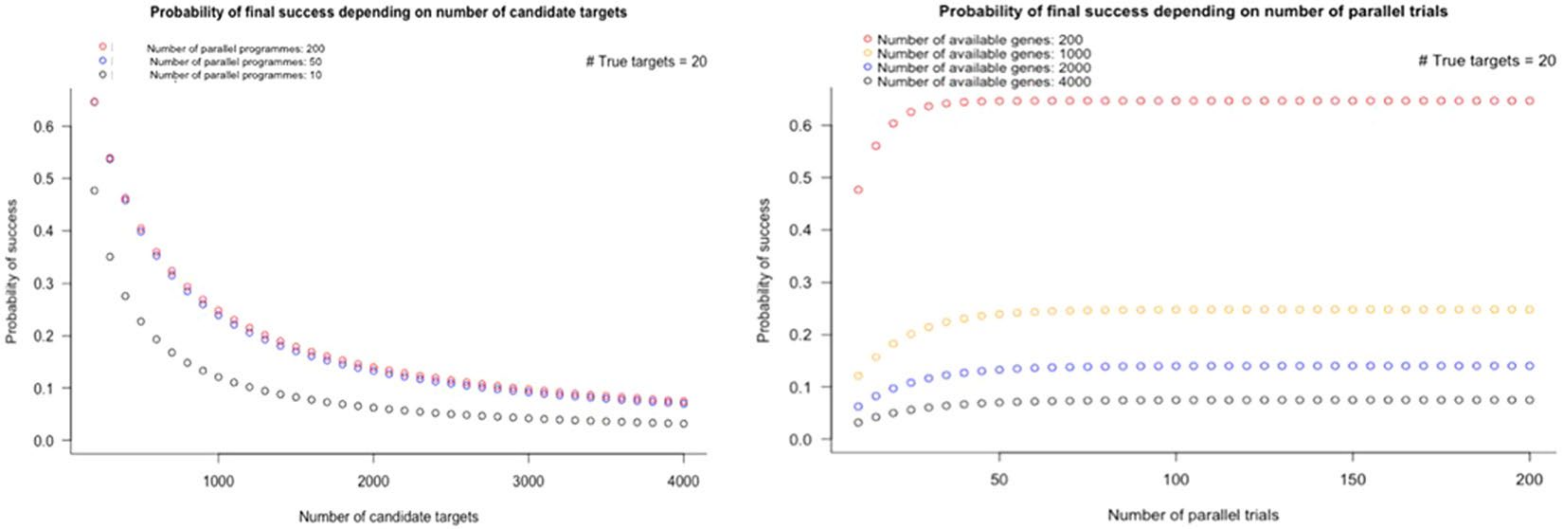
Probability of orthodox drug development success according to the number of candidate targets in the initial sampling frame (left panel) and the number of parallel preclinical development programmes pursued (right panel). The calculations assume there are 4000druggable genes and 20 causal, druggable targets per disease.

Unless *N* is very large (e.g. 200 independent preclinical programmes proceeding in parallel, each evaluating a different target), there is a very low probability of a causal, druggable target being included in the set of programmes selected for preclinical studies, based on a random pick. Let us assume one nominally positive target is pursued for clinical development under the three scenarios that generate positive findings from preclinical studies (regardless of whether they are true or false positives), and that correct target selection is the only barrier to eventual drug development success (**Assumption 9**). Under the first scenario, clinical development will always be successful, under the second it will sometimes be successful and under the fourth never successful. The overall probabilities of eventual development success are given by equations in Supplementary Note 10 and the results are shown in Tables S4 and S5 and Fig. 6. With 20 causal, druggable targets to find, increasing the number of parallel preclinical programmes from 20 to 50 to 200 has a modest impact on drug development success if these are picked from the full set of 4000 druggable proteins. The expected number of true positives will only be greater than the number of false positives if the set of targets in the sampling frame is relatively low (<400 targets) and all causal, druggable targets are retained in the sample. This emphasises the need for very strong priors before embarking on a drug development programme.

#### Probability of repurposing success

It would appear attractive to identify new disease indications for drugs that failed to show efficacy for the original indication, but which have proved safe in man; or to expand indications for a drug already effective in one disease to another condition (Table S6). However, repurposing or indication expansion relies on the assumption that different diseases share at least some common drug targets. How likely is this?

The probability of repurposing success can be considered from three perspectives:

- How many diseases are likely to be influenced by the perturbation of a single therapeutic target?
- How many diseases need to be considered for at least one pair of diseases to share a common therapeutic target, under the assumption of independence?
- How many diseases need to be studied to find at least one that will be affected by pharmacological perturbation of a particular target of interest?

*Diseases influenced by perturbation of a single protein*: We showed previously in equation 2 (${\rm{assuming}}\,\bar{C}=100$, *N*_*D*_ = 10,000, and *N*_*G*_ = 20,000):

11 $${P}_{C}=\frac{{N}_{CD}}{{N}_{GD}}=\frac{C}{{N}_{G}}=0.005$$

With *P*_*C*_ = 0.005 the expected number diseases (*E*_*D*_) affected by any given gene (with standard deviation *S*_*D*_) is given by:

$${E}_{D}={P}_{C}\times {N}_{D}=0.005\times 10,000=50$$

$${S}_{D}=\sqrt{(1-{P}_{C})\times {P}_{C}\times {N}_{D}}=\sqrt{0.995\times 0.005\times 10,\,000}=7$$

*E*_*D*_ declines the fewer diseases (*N*_*D*_) under consideration, or if $\bar{C} < 100$ (see Table S2). Since the estimate of *E*_*D*_ should be precisely the same for a gene encoding a druggable as a non-druggable target, under **Assumption 5**, it can be inferred that even the most specific of medicines is likely to influence a range of conditions; leading either to mechanism-based adverse effects, efficacy in more than one condition, or some combination of the two. In fact, under the assumptions above, we are 95% confident that perturbation of a therapeutic target will affect between 36 and 64 diseases and only 1 in 1000 targets would affect 28 or fewer conditions.

*Shared therapeutic targets*: Consider two diseases. If we assume $\bar{C}=100$, the first disease in the pair could have any 100 of the 20,000 genes in the genome in its causal set. The probability of the second disease sharing a number *x*_1_ of the 100 genes already involved in the first disease is given by the hypergeometric distribution:

$$P({x}_{1})=\frac{(\begin{array}{c}100\\ {x}_{1}\end{array})(\begin{array}{c}20000-100\\ 100-{x}_{1}\end{array})}{(\begin{array}{c}20000\\ 100\end{array})}$$

So, the probability that the two diseases do not share any causal gene is:

$$P({x}_{1}=0)=\frac{(\begin{array}{c}100\\ 0\end{array})(\begin{array}{c}20000-100\\ 100-0\end{array})}{(\begin{array}{c}20000\\ 100\end{array})}=0.605$$

If we study a third disease, the probability of that disease sharing *x*_2_ of the 200 genes involved in the previous two diseases would be:

$$P({x}_{2})=\frac{(\begin{array}{c}200\\ {x}_{2}\end{array})(\begin{array}{c}20000-200\\ 100-{x}_{2}\end{array})}{(\begin{array}{c}20000\\ 100\end{array})}$$

So, the probability of the third disease not sharing a single gene with the other two (*x*_2_ = 0) is:

$$P({x}_{2}=0)=\frac{(\begin{array}{c}200\\ 0\end{array})(\begin{array}{c}20000-200\\ 100-0\end{array})}{(\begin{array}{c}20000\\ 100\end{array})}=0.365$$

So the total probability of the three diseases not sharing any of the genes is:

$$P({x}_{1}=0)\times P({x}_{2}=0)=0.605\times 0.365=0.221$$

With four diseases, the probability of none of them sharing a gene is <5%, and for eight diseases it is less than 1 in a million: it is almost certain that at least two diseases from this pool of eight, will share at least one common susceptibility gene.

*Number of diseases that need to be studied to identify at least one that is affected by perturbation of a given target*: The answer to the third question follows the same reasoning as that used previously to estimate the number of drug development programmes that need to be pursued in parallel to have at least a 90% or greater chance of at least one development success. With *P*_*C*_ = 0.005(i.e. focusing on the druggable genome), 460 diseases would need to be studied to have ≥90% chance of identifying at least one condition that is causally affected by perturbation of a particular target of interest. When $\bar{C}=1000$, the number of diseases that need to be studied is 45.

Despite these considerations, the ultimate challenge for repurposing remains the same as that for *de novo* drug development: knowing precisely which targets are important in which diseases and therefore which targets are shared among a set of diseases of interest. We show in the next section how a human genomic approach to drug development is well placed to address this critical issue.

### Part B. Target identification through GWAS

Design features of GWAS that address the major contributions to drug development failure are: (1) investigation of humans, not animal models; (2) a much more stringent∝ value (typically 5 × 10^−8^) than is routine in orthodox preclinical studies^49^; (3) concurrent interrogation of every drug target in parallel obviating the need for a selection step; and, (4), the naturally randomised allocation of genetic variants that mimics the design of a randomised controlled trial.

To attempt to quantify potential efficiency gains from using GWAS rather than standard preclinical models for drug target identification, we review the number of licensed drug targets already ‘rediscovered’ by GWAS; estimate the expected ‘yield’ of drug targets from a well powered GWAS in a disease of interest; and the predictive accuracy of GWAS for drug target identification, compared to the conventional preclinical study-based approach.

#### Rediscovery of licensed drug target-disease indications by a GWAS

Examples of the apparently sporadic ‘rediscovery’ by GWAS of drug targets already exploited for the treatment of the corresponding disease, as well as rediscoveries of the known mechanism-based adverse effects of several drug classes are included in Table 3 and a linked paper^33^. Are such rediscoveries serendipitous or predictable?

**Table 3:** (following pages). Illustrative examples of mapping SNPs curated in the GWAS catalogue to genomic linkage dis-equilibrium (LD) intervals containing targets of licensed and clinically used drugs (adapted with modification from.*Finan C, Gaulton A, et al. Sci. Translational Med. 2017 Mar 29*; *9(383). pii: eaag1166. doi: 10.1126/scitranslmed.aag1166*).

Gene	Drug	Molecule type	Curation code	GWAS EFO term	Drug Indication (FDB)	Associated Variant	Reference (pmid)	Minimun distance from druggable gene (bp)	Distance rank of druggable gene	Number of Genes In LD interval	Number of Druggable genes in LD interval
ALDH2	DISULFIRAM	Small molecule	1	alcohol drinking|drinking behavior	Alcoholism (adjunctive treatment)	rs11066280| rs12229654| rs2074356|rs671	21270382| 21372407| 23364009| 24277619	6016–790230	1–18	22–33	2–4
PDE4D	AMINOPHYLLINE	Small molecule	1	asthma	Acute asthma|Acute exacerbation of chronic obstructive airways disease|Bronchial asthma|Chronic obstructive pulmonary disease|Left ventricular failure - cardiac failure - cardiac asthma|Reversible airways obstruction|Routine maintenance therapy in chronic bronchitis and asthma	rs1588265	19426955	448153	1	2	1
IGF1R	MECASERMIN	Protein	1	body height	Growth failure due to primary IGF-1 deficiency	rs2871865	20881960| 25429064	2696	1	2	1
TNFSF11	DENOSUMAB	Antibody	1	bone density	Prevention of skeletal related events in advanced malignancy involving bone|Treatment of bone loss associated with hormone ablation in prostate cancer|Treatment of osteoporosis in postmenopausal women to prevent fractures	rs17536328| rs9525638	24945404	6157–8295	1	1	1
ESR1	TAMOXIFEN CITRATE	Small molecule	1	breast carcinoma	Carcinoma of breast|Infertility - female - anovulatory	rs140068132| rs3757318|rs9383938	22976474| 23535729| 25327703	9531–63713	1–2	2	1
PLG	ALTEPLASE	Enzyme	1	coronary heart disease|large artery stroke|stroke	Acute ischaemic stroke: fibrinolytic treatment| Thrombolysis in acute myocardial infarction| Thrombolysis of occluded central venous access devices|Thrombolytic treatment in acute massive pulmonary embolism	rs10455872	24262325	113152	3	3	2
TNF	ADALIMUMAB	Antibody	1	Crohn’s disease	Active polyarticular juvenile chronic arthritis-inadequate response to MTX|Active progressive rheumatoid arthritis|Moderate to severe plaque psoriasis: when other treatment is inappropriate|Moderate/severe ulcerative colitis: when other treatment is inappropriate|Rheumatoid arthritis when inadequate response to DMARDs incl. methotrexate|Severe active rheumatoid arthritis|Severe ankylosing spondylitis in adults if conventional therapy inadequate|Treatment of active & progressive psoriatic arthritis when DMARD inadequate|Treatment of active Crohn’s disease	rs1799964	21102463	1036	2	13	4
CACNA1D	AMLODIPINE	Small molecule	1	diastolic blood pressure	Essential hypertension when stabilised on same ingreds.in same proportions|Hypertension-not adequately controlled by individual components|Prinzmetal’s angina|Prophylaxis of chronic stable angina pectoris|Treatment of essential hypertension|	rs9810888	25249183	106912	1	1	1
NPC1L1	EZETIMIBE	Small molecule	1	LDL cholesterol|low density lipoprotein cholesterol measurement|total cholesterol measurement	Combined hyperlipidaemia: lipid lowering therapy adjunct to diet|Homozygous familial hypercholesterolaemia (adjunct to statin therapy)|Homozygous familial hypercholesterolaemia: Adjunct to diet|Homozygous sitosterolaemia (phytosterolaemia)|Primary hypercholesterolaemia (hyperlipidaemia type IIa): Adjunct to diet|Primary hypercholesterolaemia: lipid lowering therapy adjunct to diet	rs2072183	20686565| 24097068	1734	1	1	1
PPARA	GEMFIBROZIL	Small molecule	1	LDL cholesterol|low density lipoprotein cholesterol measurement|total cholesterol measurement	Mixed hyperlipidaemia when statin is contraindicated or not tolerated|Primary hypercholesterolaemia: lipid lowering therapy adjunct to diet|Reduction of cardiac events in hypercholesterolaemia|Severe hypertriglyceridaemia with or without low HDL cholesterol	rs4253772	24097068	12050	1	7	2
CASR	CINACALCET HYDROCHLORIDE	Small molecule	1	calcuim measurment	Homoeopathic|Hypercalcaemia due to malignant disease|Hypercalcaemia in primary HPT when parathyroidectomy contraindicated|Secondary hyperparathyroidism in end stage renal disease: treatment	rs17251221| rs1801725	20661308| 20705733| 24068962	1585–12095	1	5	1
IL6R	TOCILIZUMAB	Antibody	1	rheumatoid arthritis	Active juvenile idiopathic arthritis (unresp to NSAIDs) in comb with MTX|Active juvenile idiopathic arthritis when inadequate response to NSAIDs|Rheumatoid arthritis (unresp to DMARD/TNF inhib.) in comb with methotrexate|Rheumatoid arthritis when inadequate response to DMARDs incl. methotrexate	rs2228145	24390342	14956	1	1	1
TNF	ADALIMUMAB	Antibody	1	rheumatoid arthritis	Active polyarticular juvenile chronic arthritis-inadequate response to MTX|Active progressive rheumatoid arthritis|Moderate to severe plaque psoriasis: when other treatment is inappropriate|Moderate/severe ulcerative colitis: when other treatment is inappropriate|Rheumatoid arthritis when inadequate response to DMARDs incl. methotrexate|Severe active rheumatoid arthritis|Severe ankylosing spondylitis in adults if conventional therapy inadequate|Treatment of active & progressive psoriatic arthritis when DMARD inadequate|Treatment of active Crohn’s disease	rs2596565	24532677	190015	24	145	27
ABCC8	GLIPIZIDE	Small molecule	1	type II diabetes mellitus	Non insulin dependent diabetes mellitus when diet has failed	rs5219	19056611	4860–5802	3	5	3
ABCC8	GLYBURIDE	Small molecule	1	type II diabetes mellitus	Type 2 diabetes (NIDDM) not controlled by diet,weight loss & exercise alone	rs5215|rs5219	17463248| 17463249| 19056611| 24509480	4860–5802	3	5	3
ABCC8	NATEGLINIDE	Small molecule	1	type II diabetes mellitus	Control of type-2 diabetes (NIDDM) with metformin if metformin inadequate	rs5219	19056611	4860–5802	3	5	3
ABCC8	REPAGLINIDE	Small molecule	1	type II diabetes mellitus	Control of type-2 diabetes (NIDDM) with metformin if metformin inadequate|Type 2 diabetes (NIDDM) not controlled by diet,weight loss & exercise alone	rs5219	19056611	4860–5802	3	5	3
KCNJ11	GLIMEPIRIDE	Small molecule	1	type II diabetes mellitus	Type 2 diabetes (NIDDM) not controlled by diet,weight loss & exercise alone	rs5219	19056611	1224–1306	1	5	3
KCNJ11	GLIPIZIDE	Small molecule	1	type II diabetes mellitus	Non insulin dependent diabetes mellitus when diet has failed	rs5219	19056611	1224–1306	1	5	3
KCNJ11	GLYBURIDE	Small molecule	1	type II diabetes mellitus	Type 2 diabetes (NIDDM) not controlled by diet,weight loss & exercise alone	rs5215|rs5219	17463248| 17463249| 19056611| 24509480	1224–1306	1	5	3
KCNJ11	NATEGLINIDE	Small molecule	1	type II diabetes mellitus	Control of type-2 diabetes (NIDDM) with metformin if metformin inadequate	rs5219	19056611	1224–1306	1	5	3
KCNJ11	REPAGLINIDE	Small molecule	1	type II diabetes mellitus	Control of type-2 diabetes (NIDDM) with metformin if metformin inadequate|Type 2 diabetes (NIDDM) not controlled by diet,weight loss & exercise alone	rs5219	19056611	1224–1306	1	5	3
PPARG	PIOGLITAZONE HYDROCHLORIDE	Small molecule	1	type II diabetes mellitus	Combination treatment of Type 2 diabetes with insulin|Control of type-2 diabetes if metformin+sulphonylurea therapy is inadequate|Monotherapy for type2 diabetes if overweight and metformin inappropriate|Oral combination treatment of type 2 diabetes	rs1801282	24509480	64258	1	1	1
SCN1A	OXCARBAZEPINE	Small molecule	1	Mesial temporal lobe epilepsy with hippocampal sclerosis|febrile seizures	Epilepsy - combination of both partial and tonic-clonic seizures|Epilepsy - partial seizures	rs7587026	24014518	5773–52194	1	3	1
GRIN3B	MEMANTINE HYDROCHLORIDE	Small molecule	1	Alzheimers disease	Moderate to severe Alzheimer’s disease|No information available	rs115550680	23571587	40689	8	8	2
SLC22A12	SULFINPYRAZONE	Small molecule	1	urate measurement	Gout (prophylaxis)|Gouty arthritis|Hyperuricaemia	rs2078267|rs478607	20884846| 23263486	23999–108243	2–3	2–3	2
SLC22A11	PROBENECID	Small molecule	1	urate measurement|uric acid measurement		rs17300741|rs2078267	19503597| 20884846| 23263486	6233–8364	1	1–2	1–2
SCN2A	CARBAMAZEPINE	Small molecule	2	febrile seizures	Epilepsy - grand mal|Epilepsy - partial seizures|Epilepsy - tonic-clonic seizures|Prophylaxis of manic-depressive illness unresponsive to lithium|Trigeminal neuralgia	rs3769955	25344690	14186	1	1	1
DIO1	PROPYLTHIOURACIL	Small molecule	3	thyroxine|thyroxine measurement	Hyperthyroidism|Thyrotoxic crisis|Unlicensed product	rs2235544	23408906	1189	1	4	1
PDE4D	DIPYRIDAMOLE	Small molecule	4	asthma	Alternative to exercise stress in thallium-201 myocardial imaging|Ischemic stroke: Secondary prevention (with/without aspirin)|Secondary prevention of ischaemic stroke|Secondary prevention of transient ischaemic attacks|Thromboembolism+prosthetic heart valve: prophylaxis (+oral anticoagulant)|Transient ischemic attacks: Secondary prevention (with/without aspirin)	rs1588265	19426955	448153	1	2	1
ACHE	RIVASTIGMINE	Small molecule	4	resting heart rate	Mild - moderate dementia in Alzheimer’s disease|Mild - moderate dementia in idiopathic Parkinson’s disease	rs12666989|rs314370	20639392	861–34407	3–7	9	4
ACHE	NEOSTIGMINE METHYLSULFATE	Small molecule	4	heart rate	Myasthenia gravis|Paralytic ileus|Paroxysmal supra-ventricular tachyarrhythmias|Post operative distention| Post operative urinary retention|Reversal of residual competitive neuromuscular block|Unlicensed product	rs13245899	23583979	861–34407	1–7l	9	4
CHRM2	TOLTERODINE TARTRATE	Small molecule	4	heart rate	Symptomatic treatment of urinary urgency, frequency or urge incontinence	rs2350782	23583979	62368	1	3	1

The gene encoding the drug target is listed using Human Genome Nomenclature Catalogue designation. Drug names and indications are from First Data bank. GWAS SNPs are listed according to Refseq number and physical distances are in base pairs (bp). Curation code refers to the correspondence between the treatment indication and GWAS disease or trait association (see Text). Examples are shown of treatment indication rediscoveries which refer to a drug target indication-genetic association match (Curation code 1 = precise match, code 2 = disease area match). For many of these the drug target gene is the sole occupant of the LD interval defined by the GWAS SNP. Examples come from a variety of disease areas and, for some diseases (e.g. type 2 diabetes and rheumatoid arthritis), multiple target rediscoveries are noted. Examples of rediscoveries of mechanism of action (curation code 3) and mechanism-based side effects are also seen (curation code 4).

Among diseases with at least one licensed drug treatment, the total number of targets exploited by such drugs will vary. For example, nine drug classes (corresponding to nine different drug targets) contain compounds currently licensed for the treatment of type 2 diabetes but only two therapeutic classes contain compounds licensed for treatment of dementia. We can safely assume, from the efficacy of these drugs, that their targets (along with others, yet to be identified) play a causal role in the course of those diseases.

Consider the hypothetical disease (*d*_1_), for which *g*_1_, *g*_2_ … *g*_*n*_ independent genes encode targets of drugs that have already been licensed on the basis of proven efficacy in the condition. Let us assume that a GWAS in disease *d*_1_ utilises a genotyping array with adequate coverage of all *n*licensed drug target genes, that the probability of missing such a target is the false negative rate(*β*) and therefore there is a probability ((1 − *β*_1_), (1 − *β*_2_) … (1 − *β*_*n*_)) of detecting the genetic association at each of these loci. Thus (1 − *β*_*i*_) is the power (or the detection rate) for a real effect of gene *g*_*i*_in disease *d*_1_.

We consider testing for a genetic association at the locus encoding each drug target in each hypothetical GWAS of *d*_1_ to be an independent trial (**Assumption 7**), where success equates to detection of an association at the locus and failure to overlooking the association. If there are 3 licensed drug targets in disease *d*_1_ available for rediscovery, and the power to detect true associations is the same at all 3 target loci i.e. (1 − *β*_1_) = (1 − *β*_2_) = (1 − *β*_3_) = (1 − *β*). A GWAS in *d*_1_ might detect 0, 1, 2 orall 3 of the known drug targets, and the probability that each of these situations occurs is given by the binomial distribution:

$$P\,(x)=(\begin{array}{c}{n}_{1}\\ x\end{array}){(1-\beta )}^{x}{\beta }^{{n}_{1}-x}$$

$$P\,(x)={\rm{the}}\,{\rm{probability}}\,{\rm{of}}\,{\rm{detecting}}\,x\,{\rm{licensed}}\,{\rm{drug}}\,{\rm{targets}}$$

$${n}_{1}={\rm{the}}\,{\rm{number}}\,{\rm{of}}\,{\rm{licensed}}\,{\rm{drug}}\,{\rm{targets}}\,{\rm{in}}\,{\rm{disease}}\,{d}_{1}$$

$${n}_{1}-x={\rm{the}}\,{\rm{number}}\,{\rm{of}}\,{\rm{undetected}}\,{\rm{licensed}}\,{\rm{drug}}\,{\rm{targets}}\,{\rm{in}}\,{\rm{disease}}\,{d}_{1}$$

$$\beta ={\rm{Type}}\,{\rm{II}}\,({\rm{false}}\,{\rm{negative}})\,{\rm{error}}\,{\rm{rate}}\,{\rm{at}}\,{\rm{each}}\,{\rm{genetic}}\,{\rm{locus}}$$

If *β* = 0.2, the probability (*P*) that a GWAS in disease *d*_1_:

- Detects none of the three licensed drug target genes, *P*(*x* = 0) = *β*^3^ = 0.008
- Detects only one of the three licensed drug target genes but misses the remaining two, *P*(*x* = 1) = 3*β*^2^(1 − *β*) = 0.096
- Detects only two of the three licensed drug target genes but misses the other, *P*(*x* = 2) = 3*β*(1 − *β*)^2^ = 0.384
- Detects all three licensed drug target genes, *P*(*x* = 3) = (1 − *β*)^3^ = 0.512
- Detects at least one of the three licensed drug target genes, *P*(*x* > 0) = 1 − *β*^3^ = 1 − 0.008 = 0.992

In general, if power at all loci in a GWAS of a disease *d*is (1 − *β*) and there are *n*_*d*_ licensed drug targets to rediscover, the expected number of drug targets rediscovered (*E*_*d*_) and its standard deviation (*S*_*d*_) will be given by:

$${E}_{d}={n}_{d}\,(1-\beta )$$

$${S}_{d}=\sqrt{{n}_{d}\,\beta \,(1-\beta )}$$

In the worked example, we would therefore expect 2.4(*SD* = 0.7) of the 3 possible licensed drug targets to be rediscovered, on average.

Suppose we do one GWAS for each of *K* different diseases (*d*_1_, *d*_2_ … *d*_*K*_) where, for each disease, the number of licensed targets available for rediscovery is (*n*_1_, *n*_2_, … *n*_*K*_). If we assume that the power to detect an association at gene *i* encoding the target of licensed drug is the same for all drug targets in *all* GWAS *j*, regardless of disease (i.e. (1 − *β*_*i*,*j*_) = (1 − *β*) for all *i* and *j*), then the expected number of true drug target-indication rediscoveries (*E*_*T*_) across the *K* GWAS would be the sum of the expected rediscoveries in each GWAS. Therefore:

$${E}_{T}={E}_{1}+{E}_{2}+\ldots +{E}_{K}$$

$${E}_{T}=(1-\beta ){n}_{1}+(1-\beta ){n}_{2}+\ldots +(1-\beta ){n}_{K}$$

$${E}_{T}=(1-\beta )({n}_{1}+{n}_{2}+\ldots +{n}_{K})$$

Thus,

$${E}_{T}=(1-\beta ){N}_{K}$$

Where

*N*_*K*_ = (*n*_1_ + *n*_2_ + … + *n*_*K*_) = the total number of licensed drug targets for *K* diseases

Dividing and multiplying the above equation by *K*, we obtain:

$${E}_{T}=K(1-\beta ){N}_{K}/K$$

$${E}_{T}=K(1-\beta )\bar{n}$$

Where;

$\bar{n}$ = *N*_*K*_/*K* = the average number of targets of licensed drugs per disease

The standard deviation (*SD*_*T*_) is given by:

$$S{D}_{T}=\sqrt{\beta (1-\beta )\,\bar{n}\,K}$$

Suppose a GWAS was done for each of 200 different diseases, each with power (1 − *β*) = 0.8 to detect each true licensed target, and $\bar{n}$ = 3(i.e. an average of 3 targets per disease and *N*_*K*_ = $\bar{n}$*K* = 600 potentially re-discoverable target-disease combinations in total).

The total number of licensed drug target rediscoveries from the combined dataset would be expected to be:

$${E}_{T}=(1-\beta ){N}_{K}=480$$

$$S{D}_{T}=\sqrt{0.2\times 0.8\times 600}=9.8$$

Values of *E*_*T*_ for a range of plausible values of *β* and $\bar{n}$, given *K* = 200 are provided in Table S7.

It seems reasonable to ask if the number of licensed drug target rediscoveries already made by GWAS is close to that expected from these arguments. However, the answer is not straightforward. It requires enumerating the number of GWAS that have already been done for conditions that correspond to either a treatment indication or a mechanism based adverse effect for at least one licensed drug target, and counting the total number of licensed drug targets represented across all these conditions (since some diseases may be connected with multiple licensed drug targets). Different disease terminologies used to catalogue GWAS, drug indications and adverse effects hamper these efforts. There is also a requirement to make strong assumptions about the average power of eligible GWAS to detect a true association at a gene encoding a licensed drug target.

However, the question can also be inverted: given the observed number of rediscoveries, what was the average power of GWAS to rediscover loci encoding licensed drug targets for the same indication or through a known mechanism-based adverse effect? We previously reported that GWAS to 2015 had encompassed 315 unique MeSH disease terms and led to the ‘rediscovery’ of 74 of the 670 or so known licensed drug targets, either through treatment indication, or mechanism-based adverse effect association^33^.

To estimate average power, we use:

$${E}_{T}=K(1-\,\beta )\,\bar{n}$$

$$(1-\,\beta )=\frac{{E}_{T}}{\bar{n}\,K}$$

$$(1-\beta )=\frac{74}{\bar{n}\times 315}$$

$$(1-\beta )=\frac{74}{315}\times \frac{1}{\bar{n}\,}$$

$$(1-\beta )=\frac{0.23}{\bar{n}\,}$$

If $$\,\bar{n}=1,(1-\beta )=0.23$$

If $\bar{n} < 1,(1-\beta ) > 0.23$ (as would be the case if some GWAS concerned diseases with no licensed drug target available for rediscovery)$\mathrm{If}\,\bar{n} > 1,(1-\beta ) < 0.23$

Despite the modest estimated average power, the discovery by GWAS of around 74 of the 670 or so known licensed targets, suggests the approach shows promise as a means of identifying target-disease indication pairings more systematically in the future, particularly if power were to be enhanced. We return to this point in a later section.

#### Estimated yield of druggable targets from a GWAS

In the previous section, we discussed the rediscovery of known licensed drug targets by GWAS. In this section, we discuss the potential for GWAS to specify new drug targets for common diseases prospectively.

For example, take the hypothetical disease (*d*_1_), where *C* = 100, and the expected number of causal and druggable genes is 20. Assuming a GWAS in *d*_1_interrogates each of the causal protein-coding genes with power (1 − *β*) = 0.8, the expected number of causal, druggable targets (*E*_*CT*,*d*1_) identified by such a GWAS is given by:

$${E}_{CT,d1}={n}_{CT,d1}(1-\beta )$$

(where *n*_*CT*,*d*1_is the true number of causal, druggable targets in *d*_1_)

$${E}_{CT,1}=20\times 0.8=16$$

$$S{D}_{CT,1}=\sqrt{{n}_{CT,d1}\,\beta \,(1-\beta )}=1.8$$

The probability of a GWAS detecting *x* = 0, 1, 2, 3, 4, … all 20 of the available causal, druggable targets is again given by the binomial distribution:

$$P\,(x)=(\begin{array}{c}{n}_{CT,d1}\\ x\end{array}){(1-\beta )}^{x}{(\beta )}^{{n}_{CT,d1}-x}$$

where:

*P*(*x*) is the probability of detecting *x* causal, druggable targets

*n*_*CT*,*d*1_ is the number of causal, druggable targets in disease *d*_1_ (20 in this example)

*n*_*CT*,*d*1_ − *x* is the number of causal, druggable targets not detected in the GWAS

(1 − *β*) is the power of the GWAS to detect a true association at a genetic locus (set at 0.8 in this analysis and assumed to be homogeneous for all loci)

In summary, with *C* = 100, *P*_*C*_ = 0.005, *P*_*T*_ = 0.2, i.e. *P*_*CT*_ = 0.001,a GWAS with power 1 − *β* = 0.8 at all loci would be expected to discover 16 (*SD*1.8) of the 20 available, causal, druggable targets, on average. Moreover, it would be extremely unlikely that a GWAS with (1 − *β* = 0.8) at all loci, would discover fewer than 10druggable targets.

The exceedingly stringent type 1 error rate (*α*) incorporated in GWAS (e.g. 5 × 10^−8^) also makes the probability of even one false target discovery being present among the declared associations very low indeed (Fig. 3). These calculations suggest that adequately powered GWAS (designed with appropriate consideration of the distribution of genetic effect sizes, sample size and comprehensive coverage of sequence variation in protein coding genes) should provide a highly accurate and reliable way of specifying drug targets for human diseases, addressing the high *FDR* problem that underpins inefficiency in drug development.

#### Comparison of orthodox preclinical drug development vs. human genomics as a predictive test for drug development success

Consider orthodox non-genomic preclinical (stage 1) drug development programmes with base case parameters defined by the sample space, *N*_*G*_ × *N*_*D*_ where:

$${N}_{G}={\rm{Total}}\,{\rm{number}}\,{\rm{of}}\,{\rm{protein}}-{\rm{coding}}\,{\rm{genes}}=20,\,000$$

$${N}_{D}={\rm{Total}}\,{\rm{number}}\,{\rm{of}}\,{\rm{complex}}\,{\rm{human}}\,{\rm{diseases}}=10,\,000$$

$$\bar{C}={\rm{Average}}\,{\rm{number}}\,{\rm{of}}\,{\rm{causal}}\,{\rm{genes}}\,{\rm{per}}\,{\rm{disease}}=100$$

$${N}_{T}={\rm{Total}}\,{\rm{number}}\,{\rm{of}}\,{\rm{genes}}\,{\rm{encoding}}\,{\rm{druggable}}\,{\rm{targets}}=4,\,000$$

From Eq. 7, we can infer that the proportion of causal and druggable target-disease indication pairs available for rediscovery is;

$${\gamma }_{pc}=(\frac{\bar{C}}{{N}_{G}})(\frac{{N}_{T}}{{N}_{G}}\,)=(\frac{100}{20,\,000})(\frac{4,\,000}{20,\,000}\,)=0.001$$

Setting *α*_*pc*_ and *β*_*pc*_ to 0.05 and 0.2 respectively, see previous note, and assuming it were somehow possible to evaluate every protein in every disease in such studies, then *TDR*_*pc*_ = 0.016 and *FDR*_*pc*_ = 0.984.*TDR*_*pc*_ increases to 0.14 and the *FDR*_*pc*_ falls to 0.86 if $\bar{C}=1000$ $({\gamma }_{pc}=\frac{1}{100})$, but the corresponding values are 0.002 and 0.998 if $\bar{C}=10$ $({\gamma }_{pc}=\frac{1}{10,000})$ (Table 4).

**Table 4:** *A priori* estimates of preclinical (*pc*), clinical (*c*) and overall (*o*) drug development success contrasting orthodox (non-genomic) with genomic approaches.

$\bar{C}$	*γ*_*pc*_	*α*_*pc*_	*β*_*pc*_	*FDR*_*pc*_	*S*_*pc*_	*TDR*_*pc*_ = *γ*_*c*_	*α*_*c*_	*β*_*c*_	*FDR*_*c*_	*TDR*_*c*_	*S*_*c*_	*S*_*o*_
**a**												
10	0.0001	0.05	0.2	0.9984024	0.05008	0.0015976	0.05	0.2	0.97503657	0.02496343	0.051198203	0.00256
100	0.001	0.05	0.2	0.98423645	0.05075	0.01576355	0.05	0.2	0.79601594	0.20398406	0.06182266	0.00314
1000	0.01	0.05	0.2	0.86086957	0.0575	0.13913043	0.05	0.2	0.27887324	0.72112676	0.154347826	0.00888
10	0.0001	0.00000005	0.2	0.00062455	0.00008	0.99937545	0.05	0.2	0.000039057	0.99996094	0.79953159	0.000064
100	0.001	0.00000005	0.2	0.000062434	0.0008	0.99993757	0.05	0.2	3.9023E-06	0.9999961	0.799953175	0.00064
1000	0.01	0.00000005	0.2	6.1875E-06	0.008	0.99999381	0.05	0.2	3.8672E-07	0.99999961	0.799995359	0.0064
**b**												
10	0.0005	0.05	0.2	0.99205955	0.050375	0.00794045	0.05	0.2	0.8864745	0.1135255	0.055955335	0.00282
100	0.005	0.05	0.2	0.9255814	0.05375	0.074418605	0.05	0.2	0.43736264	0.56263736	0.105813953	0.00569
1000	0.05	0.05	0.2	0.54285714	0.0875	0.45714286	0.05	0.2	0.06909091	0.93090909	0.392857143	0.03438
10	0.0005	0.00000005	0.2	0.00012492	0.00040005	0.99987508	0.05	0.2	7.8085E-06	0.99999219	0.799906309	0.00032
100	0.005	0.00000005	0.2	0.000012437	0.00400005	0.99998756	0.05	0.2	7.7734E-07	0.99999922	0.799990672	0.0032
1000	0.05	0.00000005	0.2	0.000001875	0.04000008	0.99999881	0.05	0.2	7.4219E-08	0.99999993	0.799999109	0.032

*TDR*, *FDR*, *S*_*pc*_, *S*_*c*_ and *S*_*o*_ are presented at different values of *α* (Type 1 error rate) *β* (Type 2 error rate) and *γ* (proportion causal and druggable targets).

(**a)** ${\gamma }_{pc}=(\bar{C}/{N}_{G})({N}_{T}/{N}_{G})$when the sample space is defined by ${N}_{G}\times {N}_{D}$, and (**b)** ${\gamma }_{pc}=(\bar{C}/{N}_{G})({N}_{T}/{N}_{T}\,)$ when the sample space ${N}_{T}\times {N}_{D}$ is restricted to the druggable genome. See text for details.

In striking contrast, with the same sample space but a genomic approach to target identification, where (1 − *β*) = 0.8, *α* = 5 × 10^−8^ and all 20,000 targets encoded by the genome are, by definition, interrogated simultaneously, *TDR*_*pc*_ = 0.999, and *FDR*_*pc*_ = 0.001. This is a reversal of *TDR*_*pc*_ and *FDR*_*pc*_values when compared to the orthodox (non-genomic) preclinical approach. The performance of genomic studies for target identification, based on these values of *α* and 1 − *β*, is little affected by 100-fold differences in $\overline{C\,}$ and*γ*_*pc*_ (Table 4**)**.

As we showed previously, if sampling were restricted to the a sample space demarcated by the druggable genome, *N*_*T*_ × *N*_*D*_, where;

$${N}_{D}\,={\rm{Total}}\,{\rm{number}}\,{\rm{of}}\,{\rm{complex}}\,{\rm{human}}\,{\rm{diseases}}=10,000$$

$${N}_{T}={\rm{Total}}\,{\rm{number}}\,{\rm{of}}\,{\rm{genes}}\,{\rm{encoding}}\,{\rm{druggable}}\,\mathrm{targets}\,=4000$$

$$\bar{C}={\rm{Average}}\,{\rm{number}}\,{\rm{of}}\,{\rm{causal}}\,{\rm{genes}}\,{\rm{per}}\,{\rm{disease}}=100$$

$${N}_{TD}={\rm{Total}}\,{\rm{number}}\,{\rm{of}}\,{\rm{possible}}\,{\rm{druggable}}\,{\rm{gene}}-{\rm{disease}}\,{\rm{pairs}}=4,000\times 10,000=40\times {10}^{6}$$

$${\gamma }_{pc}=(\frac{\bar{C}}{{N}_{G}})(\frac{{N}_{T}}{{N}_{T}}\,)=(\frac{100}{20,000}\,)(\frac{4,000}{4000}\,)=0.005$$

Focusing orthodox (non-genomic) preclinical studies on this restricted sample space (with conventional values for *α* and (1 − *β*) marginally increases the *TDR*_*pc*_(from 0.016 to 0.08) and reduces *FDR*_*pc*_ but also only marginally (from 0.998 to 0.920). Applying the genomic approach in the same sample space, where (1 − *β*) = 0.8, and *α* = 5 × 10^−8^, and all 4,000 druggable targets encoded by the genome are interrogated simultaneously, the already high *TDR*_*pc*_ increases to 0.9999, and the already low *FDR*_*pc*_ would fall further to 0.0001 (Table 4).

Based on **Assumption 7 (**DNA sequence variants in and around a gene encoding a drug target that alter expression or activity of the encoded protein (*cis*-acting variants), are ubiquitous in the genome) the approach of applying the usual type 1 error rate (*α*) used in a GWAS (5 × 10^−8^) but to association tests undertaken on only the 2% or so of the genome occupied by protein coding genes (or perhaps 0.5% of the genome occupied by genes encoding druggable targets) should reduce the multiple testing burden by about 50-fold compared to a standard GWAS, where association tests are undertaken genome wide. Moreover, the use of gene rather than SNP based association testing (e.g. using Predixscan^50^, VEGAS^51^ and FastBAT^52^) would also help mitigate the multiple testing burden.

It might be argued that *TDR*_*pc*_ and *S*_*pc*_ in conventional (non-genomic) preclinical pipelines could also be enhanced by simply setting a more stringent false positive rate in experiments involving cells, tissues and animal models. This is correct, but the change would have practical consequences. Very substantial increases in sample size would be required to maintain power. However, attending to the type 1 error rate issue alone fails to address the problem of the questionable validity of many animal models of human disease. It is also predicated on being able to evaluate every protein in every disease, a task we know to be beyond the capability of orthodox (non-genomic) preclinical studies based on cells, tissues and animal models.

Turning now to clinical (stage 2) development, *α*_*c*_ and 1 − *β*_*c*_ are typically set to 0.05 and 0.8 respectively, so it is also possible to examine the influence of variation in *γ*_*pc*_, *α*_*pc*_ and *β*_*pc*_ on preclinical (*S*_*pc*_), clinical (*S*_*c*_) and overall success (*S*_*o*_ = *S*_*pc*_ × *S*_*c*_), using Eqs. 9 and 10. The results are summarised in Table 4.

For orthodox (non-genomic) preclinical development, with sampling from the whole genome (where $\bar{C}=100,\,1-{\beta }_{pc}=0.8$, ${\alpha }_{pc}=0.05,\,{\gamma }_{pc}=\frac{1}{1000\,})$, *S*_*pc*_ = 0.05(*TDR*_*pc*_ = 0.016; *FDR*_*pc*_ = 0.984) and *S*_*c*_ = 0.06(*TDR*_*c*_ = 0.2; *FDR*_*c*_ = 0.8) giving an overall declared drug development success rate *S*_*o*_ = *S*_*pc*_ × *S*_*c*_ = 0.003 (Table 4).

With the same parameters $(\bar{C}=100,\,{\gamma }_{pc}=\frac{1}{1000\,})$, but with the genomic approach replacing orthodox non-genomic preclinical programme*s*, *S*_*pc*_ = 0.0008(*TDR*_*pc*_ = 0.99994; *FDR*_*pc*_ = 0.00006), *S*_*c*_ = 0.79995(*TDR*_*c*_ = 0.999996; *FDR*_*c*_ = 0.000004), and *S*_*o*_ = 0.00064. It may at first seem surprising that *S*_*pc*_ (and *S*_*o*_) is actually lower for genomic than orthodox (non-genomic) stage 1 development, because of a higher stage 1 ‘failure’ rate. However, a stage 1 ‘failure’ in a GWAS simply refers to a null association with the disease of interest of a specific gene (from all 20,000 evaluated in a single study), which is very different from the expensive failure of a lengthy orthodox preclinical development programme focusing on a single target at a time. The high ‘failure rate’ (i.e. high rate of null associations) in GWAS reflects the much more stringent *α*_*pc*_ in this type of study design, which results in a much lower *FDR*_*pc*_ and much higher *TDR*_*pc*_. Since *TDR*_*pc*_ = *γ*_*c*_, the GWAS design ensures fewer false relationships are carried forward into clinical development when compared to the non-genomic approach. Consequently, *TDR*_*c*_ is much increased with the genomic (compared to non-genomic) preclinical target identification.

## Discussion

### Summary of findings

In summary, the calculations indicate that a genomic approach to preclinical target validation has the potential to reverse the probability of drug development success when compared to the established (non-genomic) approach.

Drug development success has previously been constrained by:

- The apparently widespread contamination of the scientific literature by false discoveries, which undermines the validity of the hypotheses used to prioritise the selection of drug targets for different diseases;
- The poor predictive accuracy of orthodox preclinical studies, arising due to shortfalls in design and animal-human differences in pathophysiology;
- The limitation of such preclinical studies in only being able to study a handful of targets at a time, imposing a need for selecting only a subset of all possible targets
- The system flaw in drug development that sees the definitive target validation step (the RCT) deferred to the end of the drug development pipeline.

With reasonable assumptions about the number of protein coding genes, druggable proteins and human diseases, and using probabilistic reasoning, we estimated that the observed success rate in drug development $( \sim \frac{4}{100}$ for compounds; $\sim \frac{2}{100}{\rm{for\; targets}})$ only marginally exceeds the probability $(\frac{1}{200})$ of correctly selecting a causal, druggable protein-disease pair through a random pick from a sample space defined by the 4,000 genes that are predicted to encode druggable targets and 10,000 diseases, assuming an average of 100 causal genes per disease. With a target success rate of $\frac{2}{100}$, based on the orthodox (non-genomic) approach to target selection and validation, over 100 independent drug development programmes for each disease need to proceed in parallel to have a 90% probability of even one success.

Based on reported clinical and preclinical success rates, and making reasonable assumptions about values of clinical phase type 1 and type 2 error rates (*α*_*c*_ and *β*_*c*_),we also found evidence that the proportion of true target disease relationships studied in preclinical development is small, that these form only the minor proportion of nominally positive findings that are brought forward in to clinical phase studies. This likely contributes to the high preclinical false discovery rate and low clinical phase success rate.

Even applying the assumption that the probability of a protein influencing the pathogenesis of one disease is independent of the probability of it influencing any other, we show that it is highly likely that even small groups of diseases taken at random share at least one common target. This implies numerous opportunities should exist for therapeutic repurposing, but also that even highly specific modification of any target still runs a high risk of mechanism-based adverse effects. The balance between the two remains to be discovered. However, knowledge of the effect of target-specific perturbation on multiple disease outcomes currently remains incomplete because the orthodox approach to target identification and validation is neither systematic nor comprehensive.

In contrast to established non-genomic, approaches to preclinical drug development, GWAS deliver a methodical and reliable means of specifying the correct drug targets for a disease, provided that the genotyping arrays that are deployed have sufficient coverage of the druggable genome, and that the studies are adequately powered. GWAS differ from established non-genomic preclinical experiments for target identification in that the evidence source is the human not an animal model; the false positive (type 1) error rate is low (typically set at 5 × 10^−8^); every potential drug target is interrogated in parallel (not just a selected subset); and the study design shares features of an RCT, the pivotal step in drug development. For these reasons, we suggest that genetic studies will soon be universally regarded as an indispensable, though not exclusive element of drug development for common diseases. By improving the efficiency and reliability of target identification, GWAS and similar genetic study designs offer the potential to overturn the currently poor odds of success currently beleaguering drug development.

### Implications for drug development

Despite the opportunities highlighted by this paper, GWAS are yet to be optimally designed or sufficiently widely deployed to maximise their potential for drug development. Most genotyping arrays used in early GWAS provided incomplete coverage of variation in genes encoding druggable targets. To address this, we recently assembled variant content for the Illumina DrugDev genotyping array, designed to for low-cost, high-volume genotyping of samples to support genetic association studies for drug target selection and validation (‘druggable GWAS’)^33^.

The range of diseases studied has also been limited. The 400 or so unique diseases and biomarkers tackled by GWAS so far represents only a fraction of the thousands of disease terms listed by classification systems or ontologies, or that are observed in electronic health record datasets (Supplementary Note 4). Sample sizes in most GWAS may also have been too small to detect all contributing genes and all relevant drug targets.

GWAS up to now have also typically been undertaken one disease at a time using investigator-led, research-funded case collections. Yet, when the findings are collated, the same genetic loci or even variants are seen to contribute to more than one disorder, a phenomenon referred to as ‘pleiotropy’^53^. Pleiotropy can arise through a number of mechanisms, but where explained by the involvement of the same protein in the pathogenesis of different diseases, it unveils opportunities to repurpose therapies ineffective in one condition for another, to expand indications for already effective therapies, and to identify potential mechanism-based adverse effects of target perturbation. Undertaking GWAS one disease at a time, while efficient for accumulating large numbers of cases with a particular condition, is inefficient for the investigation of pleiotropy as a means of target validation and developing repurposing hypotheses.

To realise the full potential of genomics for drug target identification and validation, comprehensive capture of variation in the genome (by sequencing or genotyping) needs to be connected to the diversity of human phenotype at even larger scale than now, with attention to multiple biological layers and disease end-points. There are several routes to achieving this.

#### Amalgamating large cohort studies and consortia across the globe

GWAS in population based research cohort studies allows interrogation of multiple phenotypes in the same dataset. Such studies are well placed to evaluate genetic associations with mRNA and protein expression, with metabolite level and measures of organs and systems function. Even when obtained in different datasets, information of this type can be connected using a variety of statistical methods, because natural genetic variation (unaffected by disease and allocated at random) provides a fixed anchor point, exploiting the central dogma of the molecular biology that posits a unidirectional flow of information from DNA to RNA to protein^54^ and, via downstream mechanisms, to disease. In recognition of this, the Global Genomic Medicine Collaborative (G2MC) is gathering information on large cohorts worldwide^55^.

#### Embedding genomics in whole healthcare systems

However, cases of common diseases accrue slowly in cohort studies, such that power to detect the effects of common variants on such conditions may be limited. This is partly addressed by meta-analysis of summary level data from the many existing cohorts and consortia, and through the ongoing assimilation of data from very large national biobanks^56^. Nevertheless, additional effort will also be required to increase the scale, breadth and depth of disease outcomes captured. An efficient approach would be to embed genomic analysis within the healthcare setting so that information on natural genetic variation could be linked to the wealth of laboratory, imaging, and diagnostic data captured routinely during each clinical episode to provide insight both on disease aetiology and to unveil new drug targets^57^.

Some population cohort and healthcare genomics initiatives of this type are beginning, some in conjunction with Pharma (Table 5), but if their use is to be expanded, funders, healthcare providers, patients and populations will need to be convinced of the benefits of this new model for drug development. Legitimate concerns about data security and the secondary use of data also need to be addressed, an issue to which we return later. If successful, a new model of drug development might supervene because population and healthcare data typically resides outside the domain of the pharmaceutical industry within the academic and healthcare sectors, which, in many countries, are wholly or substantially state-run. In turn, this would dictate that a new funding and delivery structure might need to be established, at least for the component of drug development that relates to target identification and validation.

**Table 5:** Selected examples of Academia, Pharma, and Pharma-Academia initiatives concerning genomics and drug development.

Initiative	Partners	Drug development model	Aims
Accelerating Drug Development and Repurposing Incubator at Vanderbilt University^a^	Multiple departments at Vanderbilt University Medical Centre	Academic incubator	De-identified genotype data linked to de-identified demographic and health record data to aid precision drug development and drug repurposing
DECODE Genetics^b^	Decode is a subsidiary of Amgen, a biopharmaceutical company	Within-company	Discover genetic variation underlying human disease in the Icelandic population with the aim of diagnosing, treating and preventing disease
Open Targets^c^	GSK, Biogen, European Bioinformatics Institute, Wellcome Trust Sanger Institute	Pre-competitive, open access	Public-private initiative based on the use of genomics for drug target validation
Astra Zeneca Centre for Genomics Research	Human Longevity, Inc Wellcome Trust Sanger Institute Institute for Molecular Medicine, Finland	Within-company	‘Integrated genomics initiative to transform drug discovery and development across (AZ’s) entire therapeutic pipeline’
Eisai Andover Innovative Medicines Institute^e^	Seeking collaborations with external scientific partners	Pre-competitive research consortia	‘Executing novel therapeutic targets validated by human genetics’
Regeneron Genetics Centre^f^	Geisinger Health System, and other health service and academic partners	Within-company	‘Comparing genetic information against medical histories.to develop new means of diagnosing, preventing and/or treating medical conditions’
GSK-Regeneron UK Biobank Partnerhship^g^	GSK, Regeneron and UK Biobank	Industry academia partnership, with 9 month exclusivity period for Pharma partners	Exome sequencing of stored DNA from UK Biobank participants: 50,000 samples in year 1, 500,000 by year 3.

^a^http://online.liebertpub.com/doi/10.1089/adt.2016.772

^b^http://www.decode.com/

^c^https://www.opentargets.org/

^d^https://www.astrazeneca.com/media-centre/press-releases/2016/AstraZeneca-launches-integrated-genomics-approach-to-transform-drug-discovery-and-development-22042016.html

^e^http://us.eisai.com/research/andover-innovative-medicines-institute

^f^https://www.regeneron.com/genetics-center

^g^http://www.ukbiobank.ac.uk/2017/03/gsk-regeneron-initiative-to-develop-better-treatments-more-quickly.

**Figure 7:**
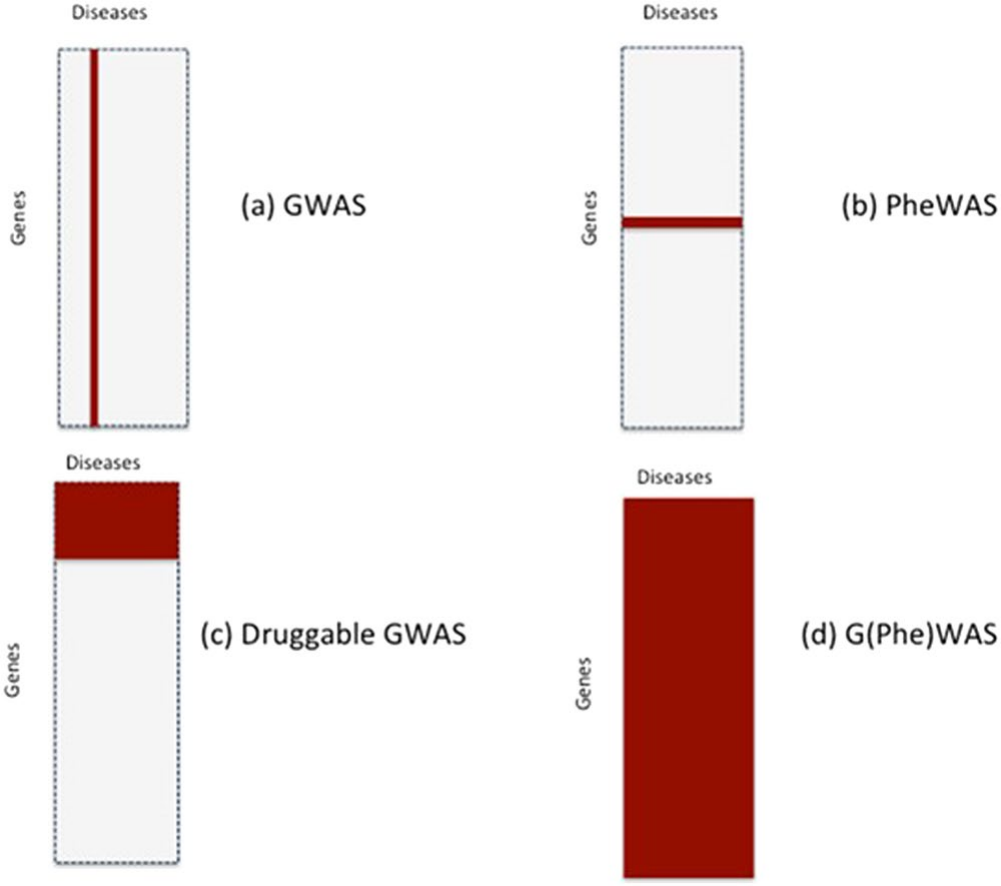
Study designs relevant to drug target identification and validation based on human genomics: (**a**) conventional genome-wide association analysis in which variation in 20,000 genes is tested against a single disease; (**b**) phenome wide association analysis of a gene encoding a drug target in which variation in a single druggable gene is evaluated against many (all) diseases; (**c**) druggable genome and phenome wide association analysis; and (**d**) whole genome and phenome wide association analysis.

There would be additional benefits from such an effort. We have focused here mainly on GWAS for matching targets to a disease (target identification). However, in related work (see Appendix 1) we (and others) have shown that the principle can also be used to anticipate the spectrum of effects of pharmacological action on a specific target on biomarkers, disease surrogates and clinically relevant disease end-points (sometimes called phenome wide association analyses; PheWAS) for target validation (Fig. 7). PheWAS (or Mendelian randomisation for drug target validation) has been used to accurately predict phase 3 trial outcomes, distinguish on- from off- target effects of drugs, correctly identify detailed biomarker profiles of therapeutic response, and to identify repurposing opportunities for licensed therapies. This underscores the view that such studies are not just useful for target identification but can also for inform drug development programmes from start to finish by indicating biomarkers of therapeutic response to measure in phase 1/2 clinical studies, and the relevant spectrum of clinical outcomes that should be ascertained in clinical trials. The incorporation of outcomes in clinical trials that are anticipated to be affected by pharmacological action on a particular target (*target-specific outcomes* of both efficacy and safety) would represent a departure from the current norm where end-points in a particular therapeutic area tend to be uniform regardless of the target being evaluated. Genetic information could also be useful for compound optimisation since the profile of biomarker effects of a SNP in a gene encoding a drug target should be those of a clean drug with no off-target actions. Where compounds are developed that have actions that are distinct from those observed in a genetic study, these may be off-target effects, and suggest that a more specific compound may need to be developed before the programme progresses. By the same principle, PheWAS would inform which clinical efficacy and safety end-points should be specified as outcomes in RCTs of compounds against a specified target. The spectrum of outcomes could differ from target to target, even for two targets being evaluated for the same primary disease indication. RCTs would need to be powered for both safety and efficacy outcomes, so that the balance between the benefits and any risk of target modification can be quantified before licensing. It should reduce the problem of mechanism-based side effects only emerging post marketing. This would also ensure that RCTs do not fail for failure to select the correct end-points, or because of the contamination of composite end-points (and thereby dilution of any treatment effect) by inclusion of outcomes that are unaffected by target modification.

There are a number of inherent assumptions and limitations to the approach we describe. We provide an extensive discussion of these issues in Supplementary Note 8. In brief, we justify our estimates of the number of human disease entities, protein coding genes, genes encoding druggable targets and the likely number of causal genes critical to the pathogenesis of common diseases. We have assumed that each gene encodes a single protein with a unique function; that a protein can influence the risk of more than one disease; that the probability that a gene influences one disease is independent of the probability that it influences another; that the probability of a protein being causal for a disease and druggable is independent; that variants in a gene encoding a drug target that affect expression or function are ubiquitous in the genome and can accurately predict the effect of pharmacological action on the same protein;, and that these variants are adequately captured by commonly used genotyping arrays. We discuss the validity of all these assumptions and the impact that the failure of these assumptions would have on the inferences that we draw in Supplementary Note 8.

Finally, most common disease genetic association studies that might inform drug development that have been performed to date have been undertaken in population-based longitudinal cohorts or case-control control datasets, where cases typically represent the first occurrence of a condition (e.g. a coronary heart disease event). However, first-in-class agents for many other common conditions, are tested or used initially patients with established disease, for prevention of disease progression or recurrence^58^. Mendelian randomization studies for target identification and validation in longitudinal clinical cohorts with established disease are few, currently limited by the available datasets, and also perhaps by potential biases arising from survivorship of, or indexing by, an initial event, that may limit inferences that can be drawn^59^. Nevertheless, the rediscovery by GWAS of over 70 drug targets suggests that genes influencing disease onset can, in many (but perhaps not all) cases, provide useful insight on targetable pathways for prevention of progression or recurrence of common conditions.

## Conclusions

The fundamental problem in contemporary drug development has been the unreliability of target identification leading to low development success rates, inefficiency and escalating cost to healthcare users. Genomics now provides a tool to address the problem directly by accurate identification of proteins that both play a controlling role in a disease and which are amenable to targeting by drugs. Maximising the opportunities arising from this paradigm requires the wider use of genomics in the healthcare setting and with this, the active participation of healthcare users in drug development. The democratisation of drug development through human genomics could have the consequence of reducing wasted investment, increasing value for investors and, eventually, reducing drug price inflation for healthcare providers. It might also provide the sorely needed stimulus for true drug development innovation, to the benefit of patients, health systems, business and society.

## Supplementary information

Supplementary Information 

Supplementary Dataset 

## Data Availability

Data sharing is not applicable to this article as no datasets were generated or analysed during the current stud.
