# Supplementary material for: Improving the odds of drug development success through human genomics: modelling study
Source: Sci Rep. 2019 Dec 11;9:18911. doi: 10.1038/s41598-019-54849-w (PMC6906499; doi:10.1038/s41598-019-54849-w)
Supplement: Supplementary file 1 — Supplementary Information [file 41598_2019_54849_MOESM1_ESM.docx]

**Improving the odds of drug development success through human genomics – SUPPLEMENTARY SECTION - NOTES**

Aroon D. Hingorani^1, 2#^ (a.hingorani@ucl.ac.uk)

Valerie Kuan^1, 2^* (v.kuan@ucl.ac.uk)

Chris Finan^1, 2^ (c.finan@ucl.ac.uk)

Felix A. Kruger^3^ (felix.kruger@benevolent.ai)

Anna Gaulton^4^ (agaulton@ebi.ac.uk)

Sandesh Chopade^1, 2^ (sandesh.chopade15@ucl.ac.uk)

Reecha Sofat^2, 5^ (r.sofat@ucl.ac.uk)

Raymond J. MacAllister^6^ (r.macallister@icloud.com)

John P. Overington^1,^ ^7^ (jpo@md.catapult.org.uk)

Harry Hemingway^2, 5^ (h.hemingway@ucl.ac.uk)

Spiros Denaxas^2, 5^ (s.denaxas@ucl.ac.uk)

David Prieto^5,9^* (d.prieto-merino@ucl.ac.uk)

Juan Pablo Casas^8^ (Juan.CasasRomero@va.gov)

^1^Institute of Cardiovascular Science, University College London, London, UK

^2^Health Data Research, UK

^3^Benevolent AI, London, UK

^4^European Molecular Biology Laboratory, European Bioinformatics Institute (EMBL-EBI), Wellcome Genome Campus, Cambridge, UK

^5^Institute of Health Informatics, University College London, London, UK

^6^Dorset County Hospital NHS Foundation Trust, Dorchester, UK

^7^Medicines Discovery Catapult, Mereside, Alderley Park, Alderley Edge, Cheshire, UK

^8^ Massachusetts Veterans Epidemiology Research and Information Center (MAVERIC), Veterans Administration, Boston MA, USA

^9^Applied Statistics in Medical Research Group, Catholic University of Murcia (UCAM), Spain

^#^Corresponding author email: [a.hingorani@ucl.ac.uk](mailto:a.hingorani@ucl.ac.uk)

*Contributed equally

**Contents**

**Supplementary note 1.** The process of drug development

**Supplementary note 2.** False discovery rate ($FDR$) in biomedical research

**Supplementary note 3.** Resolution of the high false discovery rate problem in the field of common disease genetics

**Supplementary note 4.** Estimating the number of human disease entities

**Supplementary note 5.** Estimating the number of protein coding genes in the human genome

**Supplementary note 6.** Number of causal genes for any given disease

**Supplementary note 7.** The druggable genome

**Supplementary note 8.** Assumptions, parameters and limitations

**Supplementary note 9.** Proportion of true relationships studied in contemporary drug development

**Supplementary note 10.** Calculation of the probability of success for a company that initiates $N$ parallel pre-clinical trials but will only pursue one of the signals to a further clinical trial.

**Supplementary tables.**

**Supplementary figures.**

**Supplementary section references.**

**Supplementary note 1.** The process of drug development

Developing a drug with a new mechanism of action requires fulfilling a series of tasks in sequence^1^:

1) Selecting a disease for which there is a deficit in existing therapies;

2) Identifying a pathogenic mechanism and potential drug target (almost all of which are proteins);

3) Screening for and optimising a compound (sometimes a small molecule or, increasingly, a monoclonal antibody or peptide) that specifically modulates the function of the target protein, is free of toxicity and has the desired pharmacokinetic properties;

4) Demonstrating target engagement by the compound (through the use of biomarkers or surrogate measures of the disease process); and,

5) Demonstrating efficacy against the disease end-point in tandem with an adequate safety profile.

Operationally, this is achieved in two stages: preclinical and then clinical. Preclinical studies utilise isolated cells, organoid cultures, tissue preparations ex vivo, and (if available) animal models of human disease. They test the hypothesis that the selected target plays a controlling role in the disease of interest (proof of concept) and that the compound has an adequate safety profile. If preclinical studies are encouraging, a critical decision is made to progress to clinical evaluation. This is initially through healthy volunteer studies for pharmacokinetics, dose finding and tolerability (Phase 1); and then exposure of a small number of patients often evaluating surrogate measures of disease (Phase 2). If these studies appear promising, a larger randomised (Phase 3) outcome trial will follow, typically 10 or more years after programme initiation, following several hundred million pounds of investment.

**Supplementary note 2.** False discovery rate ($FDR$) in biomedical research

A frequent misconception in biomedical research is that the false discovery rate ($FDR$) and the Type 1 (false positive) error rate ($\alpha$) are equivalent^2^. The reason this is not the case is illustrated by a hypothetical example. Imagine a field of study in which experiments are undertaken with robust design: all interventions are allocated at random and, in each experiment, the estimated treatment effect has informed the sample size such that the experimental false positive error rate ($\alpha$) is $0.05$ and the Type 2 (false negative) error rate ($\beta$), is $0.2$. The power, $(1- \beta)$, which can be conceptualised as the detection rate for a real effect, is therefore $0.8$. We introduce a third parameter ($\gamma$), the proportion of true relationships out of all those tested in the field. In the current illustration, we assume$\gamma=0.1$. **Table 1a** (main text file) illustrates that, despite the robust experimental design, these parameters dictate that $36\%$ (not $5\%$) of nominally positive experimental outcomes are false discoveries. In general, $FDR$ is related to $\alpha, \beta$ and $\gamma$ as follows:

$FDR = \frac{\alpha(1-\gamma)}{(1-\beta) \gamma+ \alpha(1-\gamma)}$

**Supplementary Table 1b and Table 2** (main text file) demonstrate how $FDR$ varies at different values of $\alpha, \beta$ and $\gamma$. Reducing $\alpha$ has the effect of reducing $FDR$. Increasing $\beta$ (equivalent to reducing power, e.g. from $0.8$ to $0.2$, which is close to the mean power recently found in a survey of preclinical studies in the field of neuroscience)^3^ increases $FDR$ (from $36\%$ to $69\%$ in this example, so that false discoveries would then outnumber true discoveries by about 2:1). $FDR$ increases as the proportion of true relationships ($\gamma$) decreases. In addition, it is not widely appreciated that real effects, even when present can be overestimated by small studies, because a positive finding must be extreme for it to exceed the usual experimental significance threshold (a similar notion to small study bias in clinical trials, and the winner’s curse^2^).

**Supplementary note 3.** Resolution of the high false discovery rate problem in the field of common disease genetics

Three major factors contributed to the resolution of the high $FDR$ problem in the field of common disease genetics in the candidate gene era. These were:

a) The development of fixed content genotyping arrays that, to a first approximation, could interrogate all genes in a genome, not just a subset of them, triggering the move from candidate gene to whole-genome (genome-wide) association studies (GWAS);

b) Recognition that a much more stringent α-value threshold would be needed in such studies to minimize false discoveries, as can be observed from **Table 2** (main text), where changing $\alpha$from $0.05$ to $5 \times{10}^{-8}$ (the now widely used genome wide Type I error rate) reverses$TDR$ and $FDR$

c) Understanding that larger sample sizes than had been usual would be needed to retain power in the context of the much stricter α-value threshold. As a consequence, clinicians and scientists began to assemble large collections of patients with diseases of interest (and controls) and, by necessity, to work together in consortia to achieve datasets of the necessary size, pooling information from individual studies in a statistically robust way using meta-analysis, a technique which, by then, had already become well-established in the clinical trial setting. A GWAS incorporating data from over $200,000$ individuals by meta-analysis would now be viewed as unexceptional. The findings from GWAS are curated by a number of repositories^4,5^ including the NHGRI-EBI GWAS catalog at <https://www.ebi.ac.uk/gwas/>.

**Supplementary note 4.** Estimating the number of human disease entities

Estimating the exact number of human diseases is a surprisingly challenging task. Clinical priorities have led to definitions of disease that rely on characteristic clusters of symptoms and signs supported to a varying degree by biophysical, laboratory, radiological or histological tests that detect abnormalities of structure or function. Defining disease on the basis of manifestations rather than cause means that diagnoses may be remote from the molecular mechanisms leading to disease, many of which remain unknown. In this paper, we set aside rare monogenic conditions, focusing instead on common (multifactorial) human diseases of potential therapeutic interest that have both a genetic and environmental contribution. A list of medical coding schemes covering such diseases, from clinical terminologies to disease classification systems, is shown in **Table S1**. Standard vocabularies of medical terms such as SNOMED CT (Systematised Nomenclature of Medicine - Clinical Terms) which includes Read Clinical Terms Version 3 (CTV3), which are used in electronic health records, capture clinically relevant data related to individuals and their care. The difficulty with using these vocabularies to enumerate diseases is that multiple codes can refer to a single disease, both because of duplicate terms (largely rectified in SNOMED CT) and the hierarchical nature of these vocabularies. In addition, disease diagnoses comprise only a proportion of the descriptive terms, with many covering symptoms, procedures, treatments, drugs and healthcare administration. The International Classification of Diseases (ICD) is widely regarded as the authoritative classification system for causes of death and illnesses. Its use in recent revisions has been broadened to medical records indexing and reimbursement. Approximately 4,000 of over 12,000 classes in the tenth revision, ICD-10, refer to health administration and external causes of morbidity and mortality and their consequences. Of the more than 8,000 remaining classes, (fewer than 500 of which are specific for rare diseases)^6,7^, overlaps occur within the hierarchical coding structure, such that a particular disease may be described by several codes. The same is true of disease and phenotype ontologies. Categorisation schemes such as the Clinical Classification Software developed by the US Agency for Healthcare Research and Quality (AHRQ), the Expanded Diagnostic Clusters (EDC) developed at Johns Hopkins University and the PheWAS Catalog designed at Vanderbilt University, collapse ICD codes into a smaller number of clinically meaningful categories that can be useful for presenting descriptive statistics.

We recognize that it is problematic to define diseases based on the use of coding schemes such as ICD-10 , utilized primarily for billing and record keeping, which offer a finite list of possible disease options, and which classify disease mainly according to appearance rather than cause. We also recognize that an ultimate outcome of research on the genetic basis of human disease may be the reclassification of disease according to molecular mechanism rather than appearance. As diseases often lie on a spectrum, with overlaps in both disease phenotypes and genetic causation, defining discrete disease entities often involves a degree of subjectivity. In the post-genomic era, biomedical ontologies have been created to provide controlled terms for biological attributes. The emphasis of coverage in the Human Phenotype Ontology (HPO) is on phenotypic abnormalities and clinical observations rather than diseases, while the Experimental Factor Ontology (EFO) describes experimental variables from the cellular to disease level in the European Bioinformatics Institute (EBI) databases. The Human Disease Ontology (DO) is a biomedical resource of standardised disease concepts organised by disease aetiology. It addresses the complexity of disease nomenclature through extensive cross mapping and integration of ICD, Online Mendelian Inheritance in Man (OMIM), Orphanet, EFO, National Cancer Institute (NCI) Thesaurus, SNOMED CT and MeSH concepts. As of 20 January 2016, the DO had 9,196 terms. The number of terms in the DO is regularly updated with technical and conceptual advances in disease phenotyping and will increase with improved understanding of molecular pathways. Therefore, given the current state of knowledge, we propose that a figure of 10,000 is a reasonable estimate of the number of common human diseases with genetic susceptibility. However, we explain in earlier sections why the various probabilities we have estimated do not depend on the absolute number of disease entities under consideration.

**Supplementary note 5.** Estimating the number of protein coding genes in the human genome

As summarised by Pertea and Salzberg^8^, estimates of the number of human protein-coding genes have been revised progressively downward since the early 1960s. Very early estimates, predating the first draft of the human genome by around 40 years, were based on extrapolation from emerging information on the amino acid sequences of proteins^9^, or theoretical considerations^10^. When the human genome project was at its planning stage, the number of human genes was projected to stand at 50-100,000 (National Institutes of Health/Department of Energy report on the Human Genome Project). However, when the initial results emerged, the estimate was revised to around 25-30,000 genes^11^. With more exhaustive sequencing of the genome and its transcripts, more detailed annotation of sequence, comparative analysis of proteomic and sequence data, and the construction of a tissue based map of the human proteome^12^, the consensus estimate of the number of protein coding genes has fallen yet again^13^. Summary statistics on the human genome are now regularly updated by the GENCODE project. The resource has catalogued a consensus value for the number of human genes since 2009, at which time 22,250 protein-coding genes were listed. In the latest data freeze (March 2016, Version 25), the number of genes listed is 19,950. For this reason, we settle on umber of human on a figure of 20,000 for the primary analyses in this paper.

**Supplementary note 6.** Number of causal genes for any given disease

Estimating a reasonable figure for the number of susceptibility genes for common diseases is a critical parameter when estimating probabilities of drug development success and requires consideration of the genetic architecture of these conditions. The approach we took in this article implicitly accepts the front-running, common-variant, common-disease hypothesis, which states that complex diseases and associated biological traits are determined by the additive (perhaps occasionally synergistic) action of common, small effect variants in a large number of human genes. Under this model, every individual carries a different repertoire of largely independently inherited variants. (This model also has implications for the success or otherwise of precision medicine therapies). The diametrically opposed hypothesis is that the association of multiple SNPs at any locus with a disease or trait seen in GWAS occurs exclusively because common SNPs mark the presence of unobserved, rare (large effect) variants present in subsets of the population (a phenomenon referred to as ‘synthetic association’) . Rare variants of this type are under-represented in the commonly used genotyping arrays used in GWAS, may be difficult to impute from haplotype reference panels, and should be better captured by exome or whole genome sequencing. However, evidence from post GWAS fine mapping studies, and a recent report on the genetic architecture of type 2 diabetes, in which whole genome sequencing allowed an unbiased survey of both common and rare variant effects in tandem, continues to provide evidence for the common variant common disease hypothesis^14^. However, it is also clear that rare, or infrequent, large effect, coding variants can also coexist in any given gene.

Our estimates of the average number of protein-coding genes that play a causal role in any given disease draws on findings from previous genome wide association studies (GWAS; **see Supplementary note 3**). This is the only routinely used study design that estimates the influence of every gene (and protein) on a disease systematically. The ability to detect disease-causing genes differs from one GWAS to the next, depends both on the underlying genetic effect in the disease of interest and the available sample size. We therefore confine our consideration to those GWAS and meta-analysis of GWAS (meta-GWAS) with the very largest sample sizes. Examples of such meta-GWAS include inflammatory bowel disease (60,000 individuals studied; 99 loci identified)^15^, type 2 diabetes (150,000 individuals; 150 loci)^16^, and coronary heart disease (200,000 individuals; 46 loci)^17^. Thus, each of these meta-GWAS has identified in the order of 100 susceptibility loci per disease. The number of disease-associated loci may not equate precisely to the number of causal genes per disease, and it may also be anticipated that yet larger sample sizes will yield yet more loci, because much of the heritability of common disorders remains unexplained^18^. There is also a school of thought that all genes (and proteins) play some role in all diseases – the infinitesimal^19^ or omnigenic model^20^– which we discuss in more detail later. However, with these caveats, we assume, initially, that there are 100 causal genes per disease on average. As it is conceivable that even more loci will be uncovered by further increases in sample size, we also estimated relevant probabilities for 1000 ‘causal’ genes per disease (corresponding to around 200 druggable genes per disease). We consider a further 10-fold increase in the number of causal genes (to 10,000 genes per disease in total) is unlikely, if only because the observed rates of drug development failure from lack of efficacy would be difficult to explain if half of all genes in the genome (corresponding to 2000 of the 4000 druggable genes under Assumption 8) critically affected risk of any given disease.

**Supplementary note 7.** The druggable genome

In 2002, at a time when the human genome was thought to contain ~30,000 protein coding genes, Hopkins and Groom estimated that 120 targets had already been exploited by licensed drugs but that ~3000 genes in total encoded proteins potentially accessible to small molecule agents, coining the term ‘the druggable genome^21^. Subsequent estimates of the druggable genome have included between 2000 and 10,000 genes depending on the data set used and assumptions made^22,23^. Our recent work in developing a genotyping array with marker coverage of genes encoding actual or potential drug targets, led to a revised estimate that approximately 4000 human genes (or about one fifth of the protein-coding genome; see **Box 4**) encode druggable proteins^24^. We use this estimate in the calculations that follow. Notably more than half of the known small molecule drug targets belong to four key gene families: class I G-protein coupled receptors (GPCRs), nuclear receptors, and ligand- or voltage gated ion channels, while targets for monoclonal antibodies or peptide therapeutics are cell membrane-bound or secreted and circulating proteins^25^. Rask-Anderson et al^26^ note around 555 targets are already exploited by currently licensed drugs (around 12% of the druggable genome) with a further 475 unique targets being the subject of investigation in clinical trials. More recently, Santos et al. estimated that FDA approved drugs for human diseases target 667 proteins encoded by the human genome^25^. Therefore, in combination, about a quarter of the druggable genome (one-twentieth of the whole genome), has already been drugged by licensed therapies or those in clinical phase development. Note again that antimicrobial treatments that interfere with targets in a pathogen rather than human host, and cancer treatment targets encoded by an abnormal cancer cell genome, distinct from the germ line, are excluded from these estimates.

**Supplementary note 8.** Assumptions and limitations

The inferences we have drawn depend on the validity of our assumptions. We now explore these in more detail before addressing some important limitations.

**Assumption 1**: *Each gene encodes a unique protein with a single function*

We assumed a 1:1 relationship between genes and proteins, implicitly arguing that any protein has a single function, echoing the historic one-gene one-protein hypothesis of Beadle and Tatum^27^. However, genes can encode alternative mRNA transcripts, some of which may be translated into different proteins^28^. Ensembl (v.87) contains 22,264 protein coding genes encoding 87,662 transcripts. Post-translational modifications increase the complexity of the proteome while some proteins may also contain domains that serve distinct functions^29^. Other proteins, referred to as ‘moonlighting proteins’ appear to have the ability to undertake alternative functions depending on the cellular context, even in the absence of splice variants or distinct functional domains^30^. Moreover, some drugs may interact with a protein-binding pocket composed of elements of two or more protein subunits, each encoded by a different gene. (An example is the benzodiazepine class of drugs that bind to GABA-A receptors at the interface of two of its subunits). Thus, the assumed 1:1 relationship between genes, proteins, protein functions and drug targets, is an undoubted simplification, posing an additional challenge for drug development to not only target the right protein, but also the correct subtype and isoform, sometimes in the right cellular context.

We recently re-estimated the extent of the druggable genome based on up to date annotations of protein coding genes, information on protein motifs targeted by drugs that have been licensed since prior estimates of the druggable genome were made, and by incorporating predicted targets of monoclonal antibody therapeutics which are either membrane-bound or secreted proteins identifiable by specific motifs in their primary structure^24^. This estimate of approximately 4479 druggable, protein-coding genes was used to inform the content of a new genotyping array developed specifically to facilitate genetic studies for drug target identification. This figure was rounded (conservatively) to 4000 genes for the illustrative calculations used in the current paper. We recognize this estimate is not fixed but likely to be revised with time as new therapeutic modalities are developed , evidenced by recent clinical successes of RNA therapeutics , of gene therapy , and of gene editing technologies that may play a therapeutic role in certain rare disorders . However, we believe it is a reasonable first approximation that drugs that act by interfering with the action of proteins readily target only a subset of human gene products, and that the factors that determine whether a protein is druggable and whether it plays a controlling role in a disease are somewhat distinct. This echoes the arguments made by others, that the challenge in drug development is to identify the proteins that lie at the intersection of druggability and disease regulation, and that human genomics is in a unique position to delineate this set of proteins for each disease of interest.

**Assumption 2**: *A given protein can influence the risk of more than one disease*

It has been estimated that nearly 20% of the genes and 5% of the SNPs currently curated by the GWAS catalogue exhibit (pleiotropic) associations with more than one trait^31^ and that many human traits share common genetic influences^32,33^. For example, variants in *GCKR* (type 2 diabetes, non-alcoholic steatotic hepatitis, uric acid, glucose, triglycerides), *IL6R* (coronary heart disease, asthma, abdominal aortic aneurysm) and *SH2B3 (*haemopoetic traits, low-density lipoprotein (LDL)-cholesterol concentration, blood pressure, autoimmune conditions, and coronary heart disease*)* have been associated with diverse diseases and traits. Although the potential mechanisms underlying pleiotropic associations are numerous^34^, one explanation is that a single protein might play a controlling role in several pathophysiological processes. Since a proportion of such genes could encode druggable targets, the corollary is that treatments proven to be effective in one disease have the potential to be successfully repurposed for another. Prior examples of repurposing successes and broadening of treatment indications also support this assumption (**Table S6**). A further consequence is that drugs used to treat one disease could have adverse effects on other conditions, depending on the direction of effect. For example, it is known now that statins, which inhibit HMG-coA reductase reduce the risk of coronary heart disease by lowering LDL-cholesterol. However, they also modestly increase risk of type 2 diabetes, an effect shown by Mendelian randomisation to be mechanism-based^35^. By implication, study designs that interrogate the association of variants in genes encoding a druggable target with a broad range of disease biomarkers and clinical diagnoses in parallel (sometimes called phenome wide association analysis – PheWAS^36^) should offer a systematic and comprehensive means to identify repurposing and indication expansion opportunities, as well mechanism-based adverse effects. We return to this point in a later section.

**Assumption 3**: *The probability of a protein influencing the pathogenesis of one disease is independent of the probability that it influences any other*

We have shown that even in the presence of this ‘independence’ assumption, it is highly likely that diseases share causal proteins, as supported by evidence from GWAS, providing one explanation for the observation of genetic pleiotropy.

In reality, the independence assumption is very likely to breakdown for certain groups of diseases, with one consequence being that certain disease groups are even more likely to share common targets, offering increased opportunity for therapeutic repurposing. Autoimmune diseases provide some of the clearest examples. As an illustration, monoclonal antibody therapeutics that target tumour necrosis factor-α for treatment of rheumatoid arthritis, also show efficacy in inflammatory bowel diseases^37^. Ustekinumab, a monoclonal antibody that targets interleukin-12/23 receptor shows efficacy in both psoriasis and inflammatory bowel disease^38^. Other examples are provided by conditions that might, at first sight, appear to be less likely to share a therapeutic target. For example, monoclonal antibodies targeting vascular endothelial growth factor have found use in the treatment of age-related macular degeneration as well as certain cancers, and it is now known that the pathogenesis of both diseases involves angiogenesis^39^. However, such agents also raise blood pressure and increase risk of thrombotic vascular events as a consequence of their mechanism of action^40^.

If diseases related by common mechanism were to be grouped as adjacent columns in the sample space (**Figure 1 main text**), and the genes encoding functionally related proteins as adjacent rows, with the sample space being marked using contours corresponding to probabilities of any target-disease paring being disease-causing, then ridges and troughs of higher and lower probability would be observed to emerge from an otherwise flat, homogenous probability space that corresponds to the independence assumption. In due course, we believe the genetic approach we describe will uncover more diseases with common underpinning, that this will enable reconfiguration of gene and disease relationships in the sample space, and will support more rational medication repurposing and indication expansion programmes^41^. Nevertheless, at present, given the very broad spectrum of human diseases, we consider our simplifying assumption to serve as a useful start point for the concepts we develop and calculations we make.

**Assumption 4**: *Drug treatments for human disease target proteins encoded in the germ line.*

We excluded from consideration the treatment of many infectious diseases, where proteins in the pathogen rather than the host are the therapeutic targets, as well as cancer, where treatment targets are mutated or aberrantly expressed proteins encoded by the abnormal genome of the cancer cell. However, with these restrictions, proteins encoded by the germ line serve as the therapeutic targets of >80% of licensed drugs^42,43^. This simplifying assumption is therefore robust for the sample space as we define it.

**Assumption 5**: *The probability that a protein affects disease pathogenesis and the probability the protein can be targeted by a drug is independent*

This assumption is more speculative. An argument could be made that genes included in our recent update of the druggable genome^24^ that encode the protein targets of small molecule drugs are more likely than other genes to be disease causing. This is because druggability predictions are based, in part, on membership of protein families containing licensed drug targets that, by definition, are both druggable and play a controlling role in disease susceptibility. However, this bias should not apply to the 2000 or so genes that were included in the druggable set because of sequence similarity to drugged proteins, or because they encode extracellular regions that are targetable by monoclonal antibodies^24^. Moreover, the converse argument is equally plausible that druggable genes are less likely than others to be pathogenic, because the druggable set is enriched for proteins with natural ligands that sub serve key cellular functions. Evolutionary forces might therefore exert purifying selection on deleterious variants in such genes, if they were to affect reproductive fitness.

Empirical evidence on this issue is limited. In our own recent analysis using findings curated in the GWAS catalogue^24^, we find that the proportion of druggable genes present in regions of LD with disease-associated SNPs is an approximately constant proportion of all genes present in such regions, that this is consistent across disease categories, and close to the proportion of druggable genes in the genome overall (i.e. $\sim4000/20,000=0.2$). This would be expected if disease association and druggability were independent. However, others have found an apparent enrichment of druggable genes among disease-associated loci^44^. We expect this uncertainty will be resolved as more GWAS are undertaken in a wider range of diseases with the purpose of drug target identification and validation.

**Assumption 6**: *Inaccurate target selection is the exclusive reason for clinical phase (stage 2) drug development failure*

Drug development can fail for numerous reasons including idiosyncratic compound toxicity, incorrect dosing, unfavourable pharmacokinetics, incorrect end-point selection, mechanism-based adverse effects and commercial considerations. Nevertheless, recent reviews have documented lack of efficacy (despite adequate target engagement) as the reason for clinical phase drug development failures in around two-thirds of cases (see main text). With this assumption, we will have over attributed failure due to inaccurate drug target selection. However, adjustment of the relevant estimates by the multiplication factor of $2/3$ (to account for other reasons for failure) would not overturn our broad conclusions, given the orders of magnitude improvement in developmental success rates predicted from the genomic approach.

**Assumption 7:** *DNA sequence variants in and around a gene encoding a drug target, that alter expression or activity of the encoded protein (cis-acting variants) are ubiquitous in the genome*

GWAS of mRNA expression and protein concentration provide hundreds of empirical examples of SNPs influencing the expression of nearby genes (acting in *cis*) leading to the concept of expression (e) and protein (p) quantitative trait loci (QTL)^45–50^. Recently, the ENCODE, ROADMAP and GTEX projects have catalogued variants with functional effects on both local (*cis*) and distant (*trans*) gene expression in a variety of cell types and tissues^51–53^. As datasets enlarge and improved proteomics platforms encompass a broader set of human proteins, we anticipate the catalogue of *cis* pQTLs will expand, providing a larger armamentarium of such variants in genes encoding druggable targets that serve as important tools for drug target identification and validation.

**Assumption 8:** *The association of cis-acting variants with biomarkers and disease end-points in a population genetic study accurately predict the effects of pharmacological modification of the encoded target in a clinical trial*

The reliability of this assumption has been demonstrated by comparisons of the associations of *cis*-acting variants in genes encoding the targets of licensed drugs in population studies, and the effect of treatments targeting the same protein in clinical trials, using a common set of biomarkers and disease outcomes as the readout. Applied examples of this paradigm have now been used to predict the eventual failure in clinical trials of first-in-class drugs for prevention and treatment of cardiovascular disease^54,55^, to separate on- from off-target effects of drugs^56,57^, and to identify indication expansion opportunities for established drugs^58^. This concordance may seem surprising given that drugs typically target the *action* of proteins while variants identified by GWAS are typically non-coding, probably influencing mRNA and thence protein *expression*. Nevertheless, the empirical findings are compelling, with recent studies indicating that the concordance between the effects of genetic variants and drugs targeting corresponding proteins can extend across scores of biomarkers and disease end-points^59^. These proof-of-concept examples (**Appendix 1**) now provide strong motivation for scaling the approach to interrogate the association of *cis*-acting variants in all druggable genes against the full spectrum of diseases and biomarkers in parallel.

Coding region (loss- and gain-of-function) variants have also been shown to be useful tools for drug target selection and validation^60,61^. As falling costs lead to an expansion in sequencing studies, including in populations with a high rates of consanguinity, thereby enriched for homozygous loss of function variants^62^, we also anticipate that a broader spectrum of druggable genome variation will be discovered encompassing rare, low frequency and common variants in both coding regions (influencing function) and non-coding regions (influencing expression) that, when linked to phenotype and disease outcome, will provide invaluable information for target identification and validation.

**Assumption 9:** *Genotyping arrays used in GWAS provide comprehensive, appropriately powered coverage of the genome, and associations discovered at any one gene are independent of those detected at any other gene*

We have made the assumption that the genotyping arrays used in GWAS provide comprehensive coverage of all genes (including all druggable genes), that all such studies are conducted such that power is 0.8 at all loci, with $\alpha=5\times{10}^{-8}$, and that the discovery of any one genetic locus is independent of any other. We recognise that in reality, power in many GWAS is likely to be much lower than 0.8 suggesting that additional loci are likely to be identified by increased sample size. We also recognise that the local correlation between SNPs (linkage disequilibrium; LD) can lead to ambiguity on the source of the association signal(s) at any locus identified by a GWAS (placing uncertainty on the role of any implicated drug target). We showed previously that GWAS to date have identied LD regions containing a single druggable gene in around $10.5\%$ of cases^24^, and $31.9\%$of such LD regions contain 2 or more genes, at least one of which encodes a druggable target. However, to begin to address the issue of verifying the causal gene(s) in an associated region, sequencing projects have led to haplotype reference panels that enable dense imputation and fine mapping of association signals^63^. *In silico* approaches based on functional annotation of the genome have been developed, as have statistical-, pathway-, and eQTL- co-localisation methods, to address this problem, together with scoring systems that assimilate results from multiple methods with various degrees of weighting^64^. An alternative approach to elucidation of causal signals with translational potential is to flip the problem by focusing genetic association studies exclusively on *cis*-acting variants within the druggable genome – ‘druggable genome wide association studies’. To that end, we recently designed the content of a new genotyping array, with dense marker coverage of genes encoding druggable targets^24^, facilitating a gene-centric approach to disease association studies for drug development. The array design also enables gene-based, not just SNP-based, association tests. The inclusion of common, non-coding as well as less frequent coding variation, should also enable the construction of allelic series^65^ (the genetic counterpart of a pharmacological dose response relationship).

**Limitations**

There are a number of limitations to our analysis.

We have argued that *cis*-acting variation is widespread in the human genome, but it may not be universal. In the absence of natural variation in a gene encoding a drug target of interest, influencing its expression or activity, it would be impossible to use the approach described to anticipate the pharmacological action of a corresponding drug. However, there may be ways of addressing this issue in the infrequent instances where this occurs. For example, in the absence of variants reliably influencing expression of the gene encoding interleukin-6, variants in the gene encoding the interleukin-6 receptor were used to model the effect of interference with interleukin-6 signaling on coronary heart disease risk, through pharmacological blockade of the receptor rather than the ligand^58^.

Theoretically, since genetic influences on protein expression or activity are present from early life, they may entrain developmental adaptation (canalization) through changes in other pathways that mitigate any biologically adverse effect on the system as a whole^66^. Thus, the null association of variants in a gene encoding a drug target of interest in a particular disease need not completely exclude it as a therapeutic target. This is because drugs, particularly for common diseases, are administered late in life, when developmental adaptation is inactive. Yet there are now numerous instances of both common (small effect) and rare (large effect) variants in genes encoding druggable targets that reliably anticipate the effects of drugs for late life diseases (see **Appendix 1**). Thus, it would seem that canalization is a more theoretical than practical consideration for genomic identification and validation of therapeutic targets.

We have observed that *cis*-acting variants in a gene encoding a drug target can anticipate both the pattern and rank order of effects of the corresponding drug on disease biomarkers. However, the effect sizes observed, particularly with common genetic variants, are typically one fifth to tenth that of the cognate drug. Thus, there remains the possibility that if certain biological actions are only observed beyond some threshold, achieved through target perturbation by a potent drug, but not by the weak effect of natural genetic variation n, such variants will fail to anticipate the full spectrum of effects of drug treatment. Thus, any discrepancy in the effects of genetic variants and drug action might arise not only from off-target actions of a drug (not shared by natural genetic variation), but also because of on-target threshold effects. The availability of common (weak effect) and rare (large effect) genetic variants in the same gene that allows the construction of an allelic series (effectively a genetic dose-response curve), may go some way toward mitigating this possibility in specific cases^67^.

We noted previously that local correlation between SNPs (LD) might lead to ambiguity on the source of the association signal(s) at any locus. Since LD can extend beyond gene boundaries, this issue can affect gene-centric as well as whole genome association studies, though perhaps less so. In such gene-centric studies, there remains the possibility that disease and biomarker associations attributed to the local gene of interest in fact arise from effects of adjacent genes. Approaches for exploring and accounting for this possibility were discussed earlier.

The genomic approach to target identification and validation we describe is also necessarily limited by the range of available phenotypes. Failure to comprehensively capture phenotypes influenced by perturbation of the target of interest, could lead to incomplete anticipation of the effect of drug treatment. Recently, the monoclonal antibody romosozumab targeting sclerostin for the treatment of osteoporosis was developed based on the observation that patients with rare mutations in the encoding gene have increased bone mass. This agent increased bone mineral density and reduced osteoporotic fracture rate in two phase 3 randomised trials but, in one of the trials, the rate of serious adverse cardiovascular events was also increased^68,69^. Since prior genetic studies, which had focused mainly on patients with rare mutations, had not evaluated cardiovascular end-points, it remains uncertain whether the apparent adverse signal of cardiovascular safety is real and if so, whether it is an on- or off-target, or threshold effect.

Finally, most common disease genetic association studies that might inform drug development that have been performed to date have been undertaken in population-based longitudinal cohorts or case-control control datasets, where cases typically represent the first occurrence of a condition (e.g. a coronary heart disease event). However, first-in-class agents for CHD, and for many other common conditions, are tested or used initially patients with established disease, for prevention of disease progression or recurrence^70^. Mendelian randomization studies for target identification and validation in longitudinal clinical cohorts with established disease are few, currently limited by the available datasets, and also perhaps by potential biases arising from survivorship of, or indexing by, an initial event, that may limit inferences that can be drawn^71,72^. Nevertheless, the rediscovery by GWAS of over 70 drug targets suggests that genes influencing disease onset can, in many (but perhaps not all) cases, provide useful insight on targetable pathways for prevention of progression or recurrence of common conditions.

In our *a priori* and *a posteriori* calculations of $\gamma_{pc}$ and other relevant metrics, we artificially reduced drug development to two steps: a preclinical component to predict target-disease pairings destined for clinical phase success (stage 1), and a clinical component (stage 2) to evaluate target-disease pairings brought forward from stage 1. The approach allowed the generation of formulas that highlight the key variables influencing drug development success, and some estimates of their values, based on observed success rates. These calculations should be viewed as no more than an illustration to help inform developers of the key variables influencing success rates.

**Supplementary note 9. Proportion of true relationships studied in contemporary drug development**

Possible outcomes from pre-clinical and clinical phase development are summarized in the embedded tables below.

| **Stage 1:**  **Preclinical development**  $\boldsymbol{(pc)}$ | **True relationship** | **No true relationship** | **All** |
| --- | --- | --- | --- |
| **Declared**  **success** | ${{TP}_{pc}=\gamma}_{pc}\left( 1-\beta_{pc} \right)$ | ${{FP}_{pc}=\alpha}_{pc}\left( 1-\gamma_{pc} \right)$ | $S_{pc}$ |
| **Declared**  **failure** | ${{FN}_{pc}=\gamma}_{pc}\beta_{pc}$ | ${TN}_{pc}=(1-\alpha_{pc})\left( 1-\gamma_{pc} \right)$ | $1-S_{pc}$ |
|  | $\gamma_{pc}$ | $1-\gamma_{pc}$ | 1 |

| **Stage 2:**  **Clinical Development**  $\boldsymbol{(c)}$ | **True relationship** | **No true relationship** | **All** |
| --- | --- | --- | --- |
| **Declared**  **success** | ${TP}_{c}=\gamma_{c}\left( 1-\beta_{c} \right)$ | ${{FP}_{c}=\alpha}_{c}\left( 1-\gamma_{c} \right)$ | $S_{c}$ |
| **Declared**  **failure** | ${FN}_{c}=\gamma_{c}\beta_{c}$ | ${TN}_{c}=(1-\alpha_{c})\left( 1-\gamma_{c} \right)$ | $1-S_{c}$ |
|  | $\gamma_{c}$ = ${TDR}_{pc}$ | $1-\gamma_{c}$ | 1 |

$\gamma=\text{proportion of }\text{true}\text{ target-disease relationships}$

$TP=$ true positive rate

$FP=$ false positive rate

$TN=$ true negative rate

$FN=$ false negative rate

$S=$ declared success rate

$1-S=$ declared failure rate

Declared preclinical successes ${(S}_{pc})$ comprise both true and false positive findings. Therefore:

$S_{pc}={TP}_{pc}+ {FP}_{pc}$ = $\gamma_{pc}\left( 1-\beta_{pc} \right) + \alpha_{pc}\left( 1-\gamma_{pc} \right)$

The proportion of true positive findings among reported preclinical successes equates to the preclinical true discovery rate ($T{DR}_{pc}$), where:

${TDR}_{pc}$ = $\frac{{TP}_{pc}}{S_{pc}}$ = $\frac{{TP}_{pc}}{{{TP}_{pc}+FP}_{pc}}$ = $\frac{\gamma_{pc}\left( 1-\beta_{pc} \right)}{\gamma_{pc}\left( 1-\beta_{pc} \right) + \alpha_{pc}\left( 1-\gamma_{pc} \right)}$

${(FDR}_{pc}=1- {TDR}_{pc})$

If a *clinical* phase drug development programme follows every declared *preclinical* success, the proportion of true target disease relationships in clinical phase development is equivalent to the *preclinical* true discovery rate, so we can write:

$\gamma_{c}$ = ${TDR}_{pc}$

(**Equation 8)**

Similarly, for clinical phase (stage 2) development:

$S_{c}$= ${TP}_{c}+ {FP}_{c}$ = $\gamma_{c}\left( 1-\beta_{c} \right) + \alpha_{c}\left( 1-\gamma_{c} \right)$

${TDR}_{c}$ = $\frac{{TP}_{c}}{{{TP}_{c}+FP}_{c}}$ = $\frac{\gamma_{c}\left( 1-\beta_{c} \right)}{\gamma_{c}\left( 1-\beta_{c} \right) + \alpha_{c}\left( 1-\gamma_{c} \right)}$

Since $\gamma_{c}$ = ${TDR}_{pc}$ (**Equation 8**)

${TDR}_{c}$ = $\frac{{TDR}_{pc} \left( 1-\beta_{c} \right)}{{TDR}_{pc}\left( 1-\beta_{c} \right) + \alpha_{c}\left( 1-{TDR}_{pc} \right)}$

(**Equation 9)**

$S_{c}= {TDR}_{pc}\left( 1-\beta_{c} \right) + \alpha_{c}\left( 1-{TDR}_{pc} \right)$

These equations underline the close mathematical relationship between preclinical and clinical discovery and success rates, which can be formalised as follows:

${TDR}_{c}$ = $\frac{{TDR}_{pc} \left( 1-\beta_{c} \right)}{{TDR}_{pc}\left( 1-\beta_{c} \right) + \alpha_{c}\left( 1-{TDR}_{pc} \right)}$

Dividing the numerator and denominator by${TDR}_{pc} \left( 1-\beta_{c} \right)$

and then rearranging:

${TDR}_{c}$ = $\frac{1}{1+\left( \frac{\alpha_{c}}{1-\beta_{c}} \right)\left( \frac{{1-TDR}_{pc}}{{TDR}_{pc}} \right)}$

${TDR}_{c}$ = $\frac{1}{1+\left( \frac{\alpha_{c}}{1-\beta_{c}} \right)\left( \frac{1}{{TDR}_{pc}}-1 \right)}$

Since,

${TDR}_{pc}$ $=\left( \frac{\gamma_{pc}\left( 1-\beta_{pc} \right)}{\gamma_{pc}\left( 1-\beta_{pc} \right) + \alpha_{pc}\left( 1-\gamma_{pc} \right)} \right)$

$\frac{1}{{TDR}_{pc}}=\frac{\gamma_{pc}\left( 1-\beta_{pc} \right) + \alpha_{pc}\left( 1-\gamma_{pc} \right)}{\gamma_{pc}\left( 1-\beta_{pc} \right)}$

Consequently,

${TDR}_{c}$ = $\frac{1}{1+\left( \frac{\alpha_{c}}{1-\beta_{c}} \right)\left( \frac{\alpha_{pc}\left( 1-\gamma_{pc} \right)}{\gamma_{pc}\left( 1-\beta_{pc} \right)} \right)}$

Rearranging,

${TDR}_{C}=\frac{1}{1+\left( \frac{\alpha_{c}}{1-\beta_{c}} \right)\left( \frac{\alpha_{pc}}{1-\beta_{pc}} \right)\left( \frac{{1-\gamma}_{pc}}{\gamma_{pc}} \right)}$

(**Equation 10)**

Both $\gamma_{c}$ and $\gamma_{pc}$ can be estimated from observed preclinical and clinical success rates as follows:

$S_{c}$= ${TP}_{c}+ {FP}_{c}$

$S_{c}$= $\gamma_{c}\left( 1-\beta_{c} \right) + \alpha_{c}\left( 1-\gamma_{c} \right)$

$S_{c}$= $\gamma_{c}-\beta_{c}\gamma_{c}+ \alpha_{c}-\alpha_{c}\gamma_{c}$

$S_{c}-\alpha_{c}$= $\gamma_{c}-\beta_{c}\gamma_{c}-\alpha_{c}\gamma_{c}$

$S_{c}-\alpha_{c}$= $\gamma_{c}(1-\beta_{c}-\alpha_{c})$

Therefore,

$\gamma_{c}$= $\frac{S_{c}-\alpha_{c}}{(1-\beta_{c})-\alpha_{c}}$

**(Equation 11)**

We previously established (**Equation 8)** that

$\gamma_{c}={TDR}_{pc}=$ $\frac{{TP}_{pc}}{S_{pc}}$

Since${TP}_{pc}$ $=\gamma_{pc}\left( 1-\beta_{pc} \right)$

$\gamma_{c}$ = $\frac{\gamma_{pc}\left( 1-\beta_{pc} \right)}{S_{pc}}$

Rearranging, we have

$\gamma_{pc}$ = $\frac{\gamma_{c}S_{pc}}{(1-\beta_{pc})}$

**(Equation 12)**

The reported clinical success rate, $S_{c}=0.1$. Assuming $\alpha_{c}=0.05, \beta_{c}=0.2$ (commonly used false positive and negative rates for clinical trials) and using **Equation 11**:

$\gamma_{c}$= $\frac{S_{c}-\alpha_{c}}{(1-\beta_{c})-\alpha_{c}}$ $=0.0667$,

Since,

${TDR}_{c}$ = $\frac{{TP}_{c}}{S_{c}}$

${TDR}_{c}$ = $\frac{\gamma_{c}\left( 1-\beta_{c} \right)}{S_{c}}$

${TDR}_{c}$ = $\frac{0.067 \times0.8}{0.1}$

${TDR}_{c}$ = 0.56

${FDR}_{c}$ = 1$-$0.56=0.44

This calculation suggests that nearly one in two declared clinical trial successes may be a false discovery.

Since $\gamma_{c}={TDR}_{pc}$ and ${TDR}_{pc}=1-{FDR}_{pc}$

${TDR}_{pc}=0.0667$

${FDR}_{pc}=1-0.0667=$ 0.9333

Now,

$\gamma_{pc}$ = $\frac{\gamma_{c}S_{pc}}{1-\beta_{pc}}$

The reported preclinical success rate, $S_{pc}=0.4$

Using the value $\gamma_{c}=0.0667$, and setting power for preclinical studies at $\left( 1-\beta_{pc} \right)=0.8,$ we have:

$\gamma_{pc}$ = $\frac{0.0667 \times0.4}{0.8}$

$\gamma_{pc}=0.03335$

In estimating $\alpha_{pc}$, we use the following:

$S_{pc}={TP}_{pc}+ {FP}_{pc}$

$S_{pc}$= $\gamma_{pc}\left( 1-\beta_{pc} \right) + \alpha_{pc}\left( 1-\gamma_{pc} \right)$

$S_{pc}$= $\gamma_{pc}-\beta_{pc}\gamma_{pc} +\alpha_{pc}- \alpha_{pc}\gamma_{pc}$

$\alpha_{pc}- \alpha_{pc}\gamma_{pc}=$ ${S_{pc}-\gamma}_{pc}+\beta_{pc}\gamma_{pc}$

$\alpha_{pc}(1- \gamma_{pc})=$ ${S_{pc}-\gamma}_{pc}(1-\beta_{pc})$

$\alpha_{pc}=$ $\frac{{S_{pc}-\gamma}_{pc}\left( 1-\beta_{pc} \right)}{\left( 1- \gamma_{pc} \right)}$

Note: the term ${S_{pc}-\gamma}_{pc}\left( 1-\beta_{pc} \right)$=${S_{pc}-{TP}_{pc}=FP}_{pc}$

Therefore $\alpha_{pc}=$ $\frac{{FP}_{pc}}{\left( 1- \gamma_{pc} \right)}(\text{see embedded table)}$

With $S_{pc}=0.4; \gamma_{pc}=0.03335$; and $1-\beta_{pc}=0.8$;

$\alpha_{pc}=0.386$

**Supplementary note 10. Calculation of the probability of success for a company that initiates** $\boldsymbol{N}$ **parallel pre-clinical trials but will only pursue one of the signals to a further clinical trial.**

Suppose industry selects $N$ targets at random from a pool of $t$ targets where only $c$ targets are causal to the disease of interest. The $N$ pre-clinical programmes will generate a number of positive signals of which the company will select **only** to progress to clinical phase following which there will be a licensing success (if the signal comes from a true target) or failure if the preclinical signal is a false positive. To calculate the probability of eventual licensing success we consider a situation where many companies repeat an experiment involving $N$ preclinical programmes only pursuing only one of the positive signals to a phase 3 clinical trial, and then calculating what proportion of such trials will result in a licensing success.

1. We first calculate the probability of having $A$ causal targets among the $N$ targets selected at random from the pool of $t$possible targets. Each company will select a different number by chance ($A = 0, 1, 2, 3\ldots$) with the probabilities of each following the hypergeometric distribution:

$$P\left( A \right)=\frac{\left( \begin{aligned} c \\ A \end{aligned} \right)\left( \begin{aligned} t-c \\ N-A \end{aligned} \right)}{\left( \begin{aligned} t \\ N \end{aligned} \right)}$$

So, if $t=4000$ with $c=20$, and we run $N=20$ pre-clinical trials then:

$$P (A=0) = 0.90$$

$$P (A=1) = 0.09$$

1. We next calculate the probability of generating true signals ($St$) and false signals (Sf): The $A$ causal targets in the $N$ programmes can generate from $0$ to $A$ signals ($St = 0, 1\ldots A$), while the non-causal target can generate from $0$to $N-A$ signals ($Sf = 0, 1, 2 \ldots N-A$). Each of these probabilities follow a binomial distribution independent from each other:

$P \left( St \right)=\left( \begin{aligned} A \\ St \end{aligned} \right)\beta^{A-St}{(1-\beta)}^{St}$

$P \left( Sf \right)=\left( \begin{aligned} N-A \\ Sf \end{aligned} \right)\alpha^{Sf}{(1-\alpha)}^{N-A-St}$

Where ($1-\beta$) and α are the probabilities that a causal and non-causal target will produce a signal respectively. The two probabilities being independent, the probability of a particular combination of signals from causal and non-causal targets is the product of the separate probabilities: $P(St, Sf) = P(St) x P(Sf).$ For example, the probability that, in a given repetition the causal targets produce $2$ signals and the non-causal targets produce three signals is $P(St=2 , Sf=3) = P(St=2) \times P(Sf = 3)$

1. The probability of selecting a real target among a combination of true and false signals ($St, Sf$) is given by the proportion of true signals: $St / (St + Sf)$

Thus, for a given $N$, $c$ and $t$, the final probability of licensing success across all possible values of $A$, $St$ and $Sf$ is:

$$P\left( Success \right)=\sum_{A=0}^{N} P(A)\left[ \sum_{St=0}^{A} \sum_{Sf=0}^{N-A} P\left( St \right)P(Sf)\left( \frac{St}{St+Sf} \right) \right]$$

**Supplementary tables**

**Table S1:** The number of terms within widely used disease classification systems and ontologies as of 24 February 2016.

| **Coding Scheme** | **Type** | **Number**  **of terms** | **Data source** |
| --- | --- | --- | --- |
| **ICD-10** | Disease classification | 12,445 | http://apps.who.int/classifications/apps/icd/ClassificationDownload/DLArea/Download.aspx |
| **Human Disease Ontology** | Ontology | 9,196 | https://github.com/DiseaseOntology/HumanDiseaseOntology/tree/master/src/ontology |
| **Human Phenotype Ontology** | Ontology | 11,683 | http://human-phenotype-ontology.github.io/downloads.html |
| **Experimental Factor Ontology** | Ontology | 17,263 | https://sourceforge.net/p/efo/code/HEAD/tree/trunk/src/efoinobo/efo.obo |
| **Expanded Diagnostic Cluster** | Disease groups | 282 | The Johns Hopkins ACG® System Version 11.0 Technical Reference Guide |
| **Clinical Classification Software** | Disease groups | 259 | http://www.ahrq.gov/research/data/hcup/icd10usrgd.html |
| **PheWAS Catalog** | Disease groups | 1,645 | https://phewas.mc.vanderbilt.edu/ |
| **SNOMED CT** | Clinical Terminology | 422,382 | https://www.nlm.nih.gov/research/umls/licensedcontent/snomedctfiles.html |
| **READ CTV3** | Clinical Terminology | 329,147 | https://isd.hscic.gov.uk/trud3/user/guest/group/0/pack/9 |

**Table S2**. Effect of varying estimates of the number of causative genes per disease ($C$), and the number of diseases ($\boldsymbol{N}_{\boldsymbol{D}}$) on the probability of selecting a causal gene-disease pair ($\boldsymbol{\gamma}_{\boldsymbol{C}}$); the probability of selecting a causal, druggable, gene-disease pair ($\boldsymbol{\gamma}_{\boldsymbol{CT}}\boldsymbol{)}$; and the number diseases influenced by any one gene (or encoded protein) ($\boldsymbol{E}_{\boldsymbol{D}}$.). Estimates assume 20,000 protein-coding genes.

| $C$ | $N_{D}$ | $\gamma_{C}$ | $\gamma_{CT}$ | $E_{D}$ |
| --- | --- | --- | --- | --- |
| 10 | 2500 | 0.0005 | 0.0001 | 1.25 |
| 10 | 5000 | 0.0005 | 0.0001 | 2.5 |
| 10 | 10000 | 0.0005 | 0.0001 | 5 |
| 100 | 2500 | 0.005 | 0.001 | 12.5 |
| 100 | 5000 | 0.005 | 0.001 | 25 |
| 100 | 10000 | 0.005 | 0.001 | 50 |
| 1000 | 2500 | 0.05 | 0.01 | 125 |
| 1000 | 5000 | 0.05 | 0.01 | 250 |
| 1000 | 10000 | 0.05 | 0.01 | 500 |

**Table S3.** Number of drug development programmes ($N$) that to be pursued in parallel to have a probability ($P$) of at least one development success. Analyses are based on either $90\%$ or $50\%$ (evens) probability of at least one developmental success, and a range of development success rates ($p$) starting with the currently observed industry wide average success rate of $0.01$ (See text for details)

| $P$(≥1 success) in *N* programmes | Within-programme  developmental success rate ($P_{S}$) | Number of parallel programmes ($N$) |
| --- | --- | --- |
| $0.9$ | $0.01$ | $229$ |
| $0.9$ | $0.02$ | $114$ |
| $0.9$ | $0.1$ | $22$ |
| $0.9$ | $0.2$ | $10$ |
| $0.9$ | $0.5$ | $3$ |
| $0.5$ | $0.01$ | $69$ |
| $0.5$ | $0.02$ | $34$ |
| $0.5$ | $0.1$ | $7$ |
| $0.5$ | $0.2$ | $3$ |
| $0.5$ | $0.5$ | $1$ |

**Table S4**: Expected yield of causal druggable targets from orthodox (non-genomic) preclinical programmes according to the number of causal targets for each disease and whether the sampling frame is the whole genome or the druggable genome.

| Number of programmes | Number of causal,  druggable targets  per disease | Number of targets in sampling frame | Expected number ($SD$) of  causal, druggable targets  among all programmes | Number causal druggable targets detected ($1-\beta=0.8$) | Number of non-relevant targets declared positive ($\alpha=0.05$) |
| --- | --- | --- | --- | --- | --- |
| $10$ | $20$ | $20,000$ | $0.01 (0.07)$ | $0.008$ | $0.49$ |
| $20$ | $20$ | $20,000$ | $0.02 (0.1)$ | $0.016$ | $1.0$ |
| $50$ | $20$ | $20,000$ | $0.05 (0.2)$ | $0.04$ | $2.5$ |
| $100$ | $20$ | $20,000$ | $0.1 (0.2)$ | $0.08$ | $5.0$ |
| $200$ | $20$ | $20,000$ | $0.2 (0.3)$ | $0.16$ | $10.0$ |
| $10$ | $20$ | $4,000$ | $0.05 (0.2)$ | $0.04$ | $5.0$ |
| $20$ | $20$ | $4,000$ | $0.1 (0.2)$ | $0.08$ | $1.0$ |
| $50$ | $20$ | $4,000$ | $0.25 (0.4)$ | $0.2$ | $2.5$ |
| $100$ | $20$ | $4,000$ | $0.5 (0.5)$ | $0.4$ | $5.0$ |
| $200$ | $20$ | $4,000$ | $1 (0.7)$ | $0.8$ | $10.0$ |
| $10$ | $200$ | $20,000$ | $0.1 (0.2)$ | $0.08$ | $0.5$ |
| $20$ | $200$ | $20,000$ | $0.2 (0.3)$ | $0.16$ | $1.0$ |
| $50$ | $200$ | $20,000$ | $0.5 (0.5)$ | $0.4$ | $2.5$ |
| $100$ | $200$ | $20,000$ | $1 (0.7)$ | $0.8$ | $5.0$ |
| $200$ | $200$ | $20,000$ | $2 (1)$ | $1.6$ | $10.0$ |
| $10$ | $200$ | $4,000$ | $0.5 (0.5)$ | $0.4$ | $0.5$ |
| $20$ | $200$ | $4,000$ | $1 (1)$ | $0.8$ | $1.0$ |
| $50$ | $200$ | $4,000$ | $2.5 (1)$ | $2$ | $2.4$ |
| $100$ | $200$ | $4,000$ | $5 (1)$ | $4$ | $4.8$ |
| $200$ | $200$ | $4,000$ | $10 (2)$ | $8$ | $9.5$ |

**Table S5.** Expected number of true and false positives in parallel drug development programmes based on a sample of targets drawn from all or part of the druggable genome based on orthodox preclinical experiments designed with ($1-\beta)=0.8$ and $\alpha=0.05$ (left hand panel). Probability of eventual drug development success taking forward one positive preclinical programme to clinical phase (right hand panel). (See text for further details)

| **Targets in sampling frame** | **True causal genes** | **Number of parallel development programmes**  **pursued** | **Expected true positives**  **in sample** | **Expected**  **false**  **positives**  **in sample** | **Positive programmes are exclusively true positives** | **Positive programmes are a mixture of true and false positives** | **No positive programmes** | **Positive programmes are exclusively false positives** | **Overall probability of a development success** |
| --- | --- | --- | --- | --- | --- | --- | --- | --- | --- |
| $4000$ | $20$ | $20$ | $0.08$ | $1.00$ | $2.9\%$ | $4.8\%$ | $33.1\%$ | $59.2\%$ | $5.0\%$ |
| $2000$ | $20$ | $20$ | $0.16$ | $0.99$ | $5.7\%$ | $9.2\%$ | $30.6\%$ | $54.5\%$ | $9.7\%$ |
| $1000$ | $20$ | $20$ | $0.32$ | $0.98$ | $10.6\%$ | $17.2\%$ | $26.0\%$ | $46.2\%$ | $18.3\%$ |
| $200$ | $20$ | $20$ | $1.60$ | $0.90$ | $33.3\%$ | $49.0\%$ | $6.5\%$ | $11.1\%$ | $60.4\%$ |
| $4000$ | $20$ | $50$ | $0.20$ | $2.49$ | $1.5\%$ | $16.8\%$ | $6.3\%$ | $75.5\%$ | $7.0\%$ |
| $2000$ | $20$ | $50$ | $0.40$ | $2.48$ | $2.7\%$ | $30.6\%$ | $5.2\%$ | $61.5\%$ | $13.3\%$ |
| $1000$ | $20$ | $50$ | $0.80$ | $2.45$ | $4.7\%$ | $51.4\%$ | $3.4\%$ | $40.5\%$ | $24.0\%$ |
| $200$ | $20$ | $50$ | $4.00$ | $2.25$ | $9.9\%$ | $89.1\%$ | $0.1\%$ | $0.9\%$ | $64.6\%$ |
| $4000$ | $20$ | $200$ | $0.80$ | $9.95$ | $0.0\%$ | $55.9\%$ | $0.0\%$ | $44.1\%$ | $7.5\%$ |
| $2000$ | $20$ | $200$ | $1.60$ | $9.90$ | $0.0\%$ | $81.2\%$ | $0.0\%$ | $18.7\%$ | $14.0\%$ |
| $1000$ | $20$ | $200$ | $3.20$ | $9.80$ | $0.0\%$ | $97.0\%$ | $0.0\%$ | $3.0\%$ | $24.8\%$ |
| $200$ | $20$ | $200$ | $16.00$ | $9.00$ | $0.0\%$ | $100.0\%$ | $0.0\%$ | $0.0\%$ | $64.7\%$ |

**Table S6**. Examples of drug repurposing


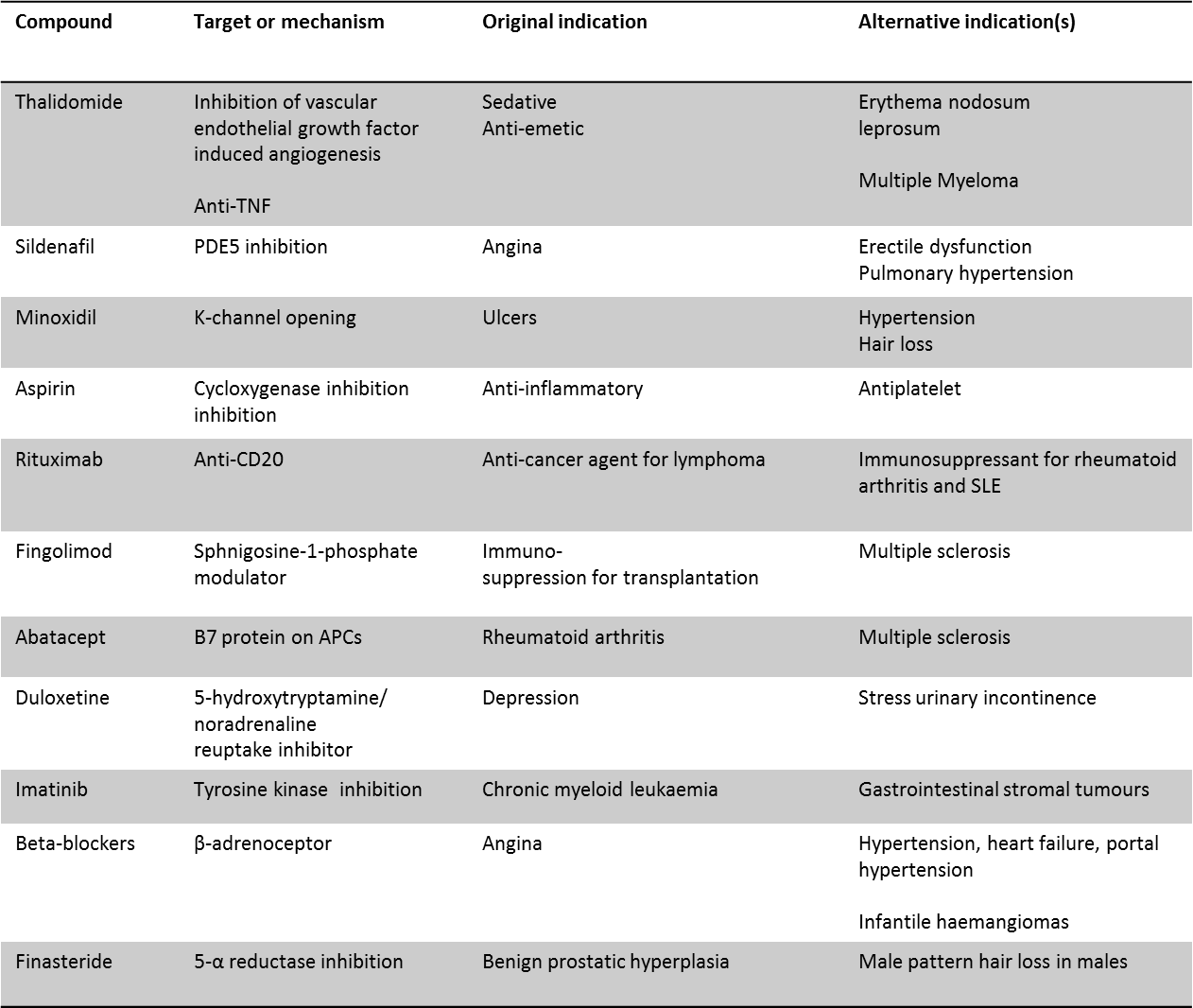


**Table S7.** Expected number of licensed drug targets rediscovered $\left( \boldsymbol{E}_{\boldsymbol{T}} \right)$by $200$ hypothetical GWAS of diseases with at least one licensed drug based on a range of plausible values of the power ($1-\beta$) to detect each genetic locus encoding a licensed drug target, and a range of plausible values for the average number of licensed drug targets per disease. (See text for further details)

| Number of licensed  drug targets per disease | Power  ($1-\beta$) | $E_{T}$($SD$) |
| --- | --- | --- |
| 1 | 0.6 | 120 (7) |
| 1 | 0.8 | 160 (6) |
| 1 | 0.9 | 180 (4) |
| 3 | 0.6 | 360 (12) |
| 3 | 0.8 | 480 (10) |
| 3 | 0.9 | 540 (7) |
| 5 | 0.6 | 600 (15) |
| 5 | 0.8 | 800 (13) |
| 5 | 0.9 | 900 (9) |
| 10 | 0.6 | 1200 (22) |
| 10 | 0.8 | 1600 (18) |
| 10 | 0.9 | 1800 (13) |

**Supplementary figures**

**Figure S1a.** Venn diagram illustrating the probabilities of selecting a causal, druggable gene-disease pair ($CD \cap TD$), a druggable gene disease pair ($TD$) and a causal, gene disease pair ($CD$) from a $200 x {10}^{6}$ gene disease pairings, $1000$ causal genes per disease and $4000$ druggable genes from the $20,000$ in the genome. (Not to scale).

**
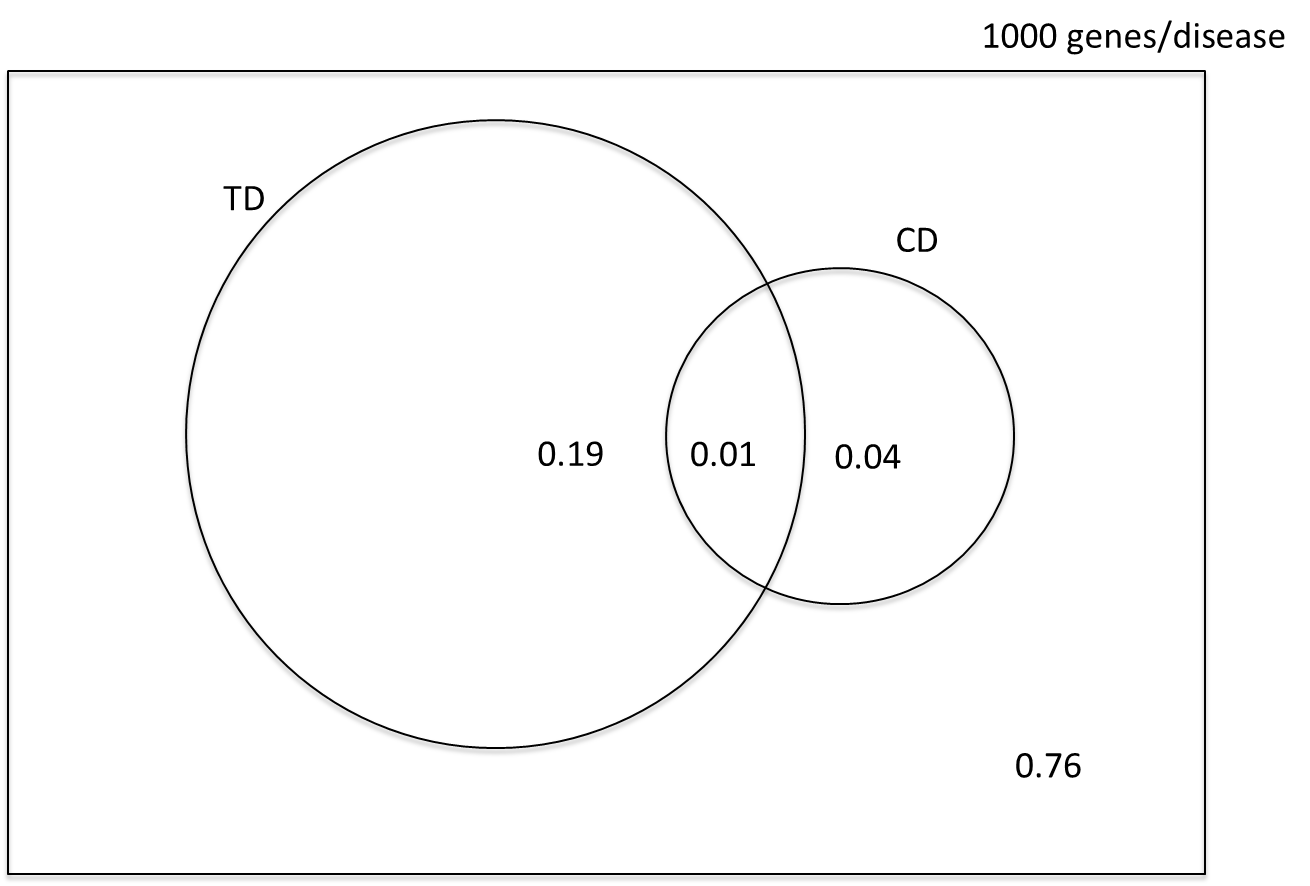
**

**Figure S1b**. Venn diagram illustrating the number of causal, druggable gene-disease

pairs ($CD \boldsymbol{\cap} TD$), druggable gene disease pairs ($TD$) and causal gene disease pairs ($CD$) from $200 x {10}^{6}$ gene disease pairings, $1000$ causal genes per disease and $4000$ druggable genes from the $20,000$ in the genome. T (Not to scale).

**
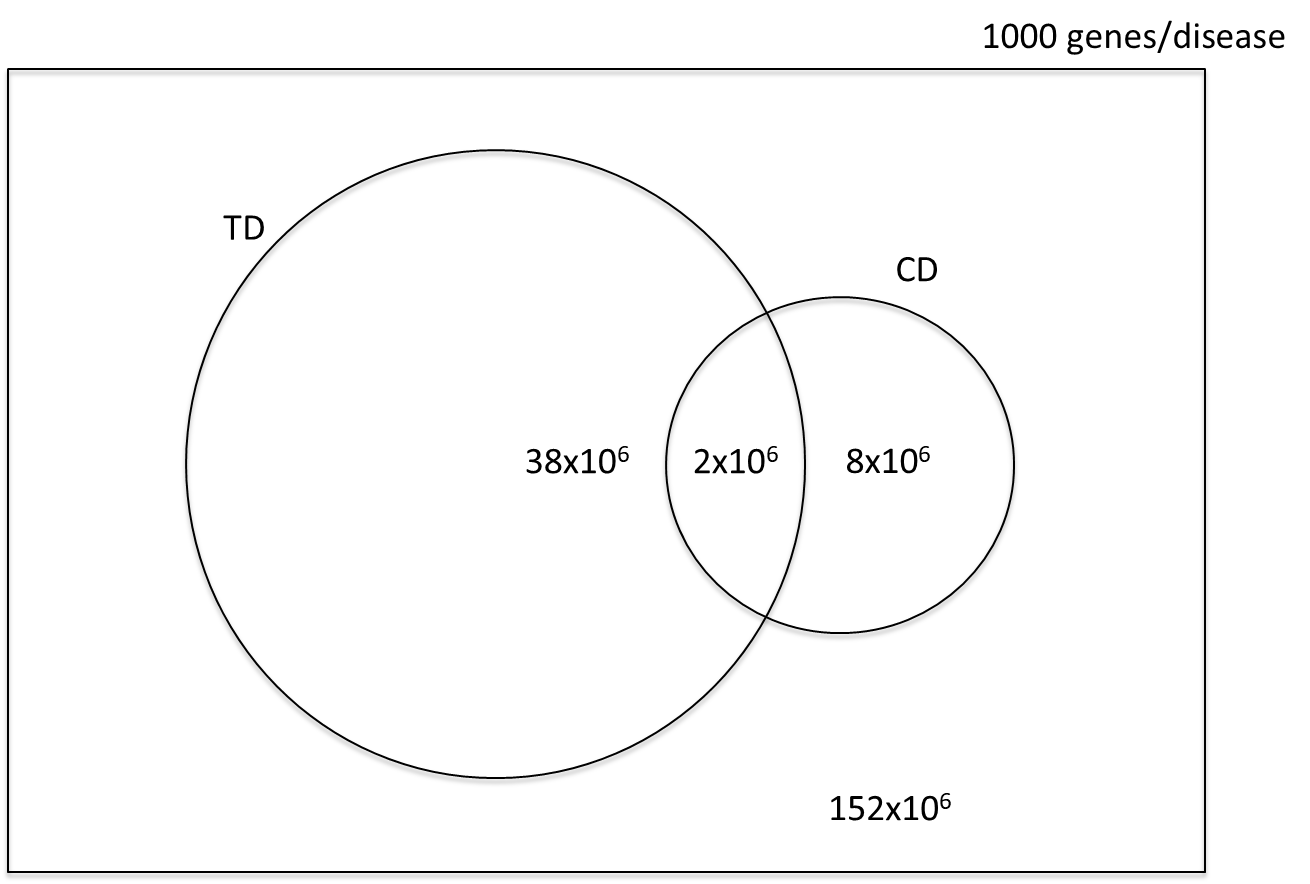
**

**Figure S2a**. Venn diagram illustrating the probabilities of selecting a causal, druggable gene-disease pair ($CD \boldsymbol{\cap} TD$), a druggable gene disease pair ($TD$) and a causal, gene disease pair ($CD$) from a sample space of $200 x {10}^{6}$ gene disease pairings, 10 causal genes per disease and $4000$ druggable genes from the $20,000$ in the genome. The dashed red circle encloses a probability space restricted to druggable genes. (Not to scale).

**
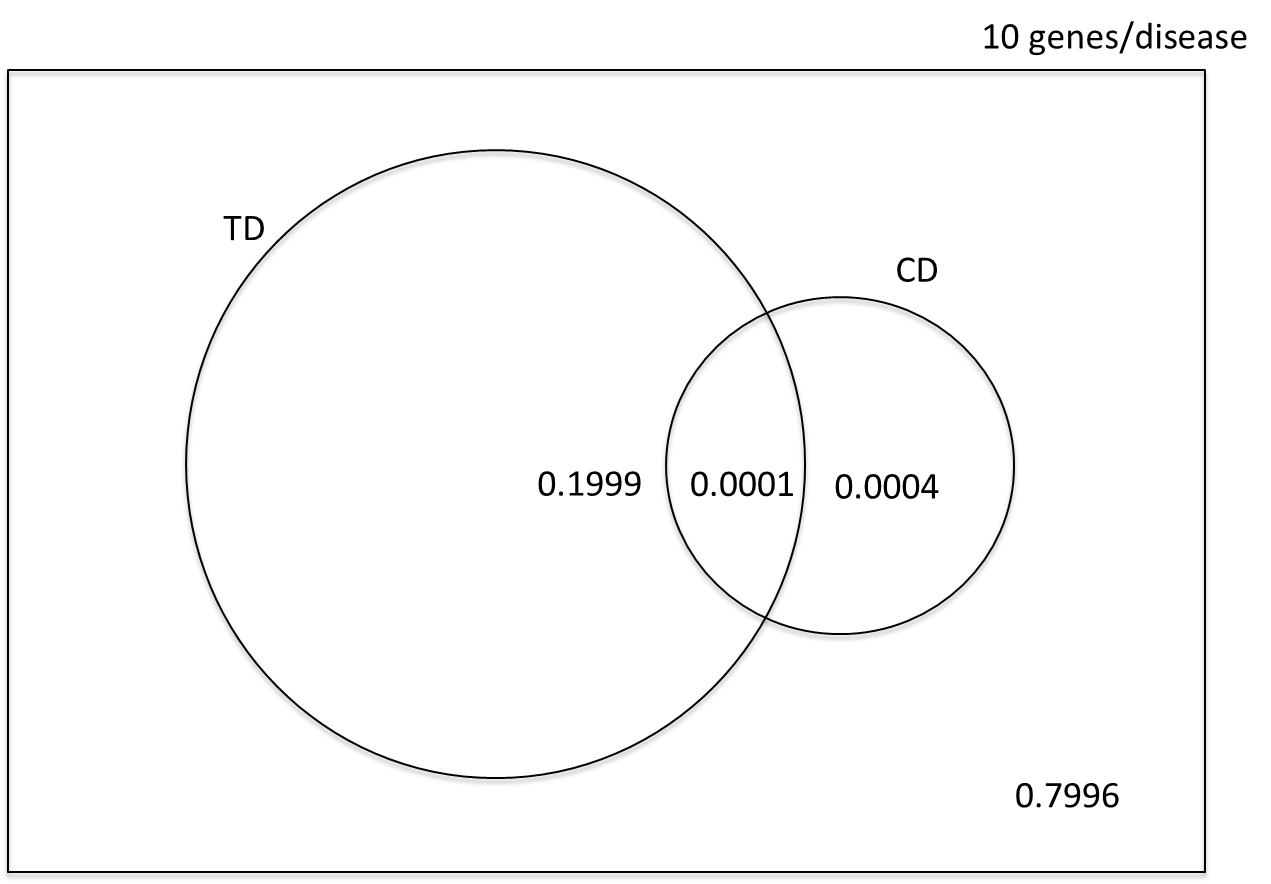
**

**Figure S2b**. Venn diagram illustrating the number of causal, druggable gene-disease

pairs ($CD \boldsymbol{\cap} TD$) , druggable gene disease pairs ($TD$) and causal gene disease pairs ($CD$) from a sample space of $200 x {10}^{6}$ gene disease pairings, $10$ causal genes per disease and $4000$druggable genes from the $20,000$ in the genome. The dashed red circle encloses a probability space restricted to druggable genes. (Not to scale).

**
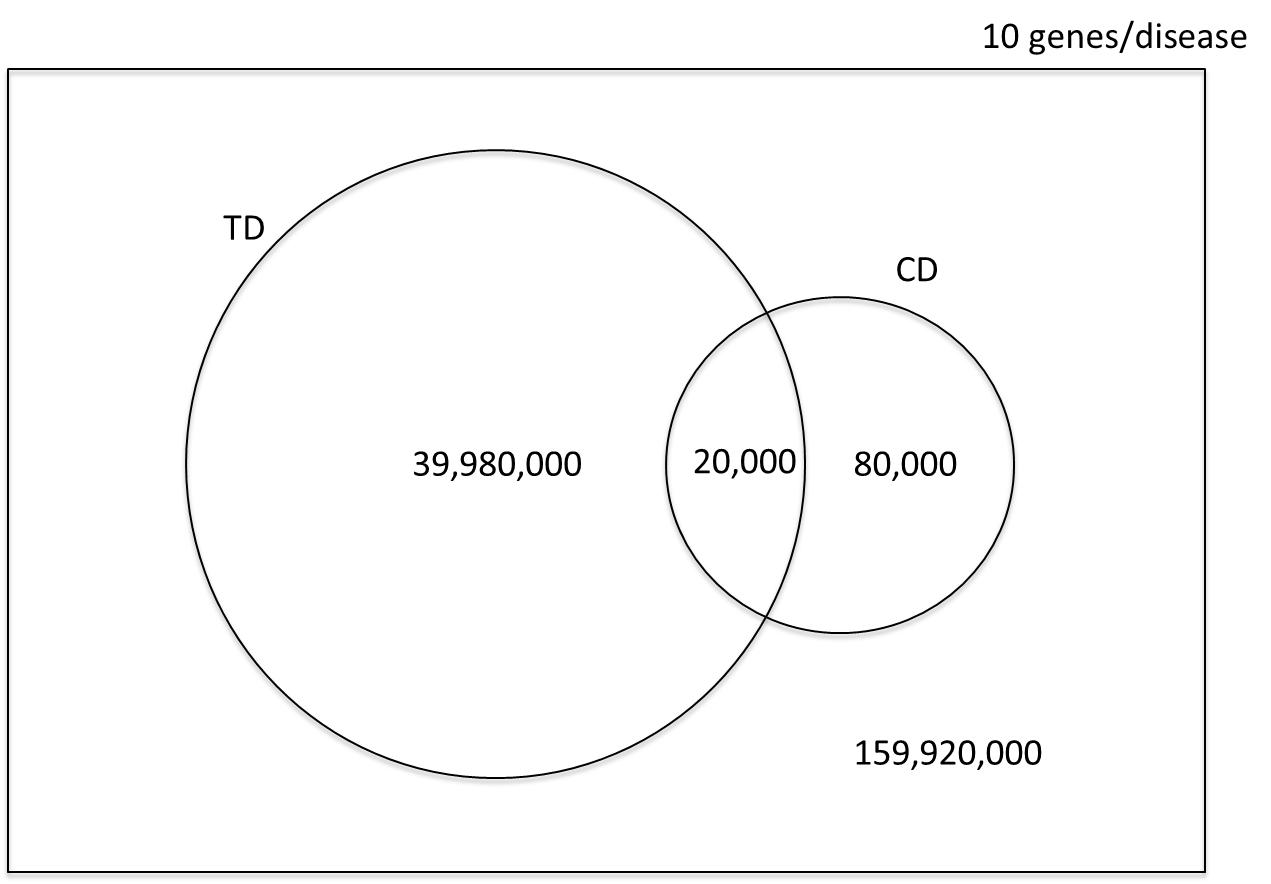
**

**Supplementary references**

1. Hughes, J. P., Rees, S. S., Kalindjian, S. B. & Philpott, K. L. Principles of early drug discovery. *British Journal of Pharmacology* (2011). doi:10.1111/j.1476-5381.2010.01127.x

2. Colquhoun, D. An investigation of the false discovery rate and the misinterpretation of p-values. *R. Soc. Open Sci.* (2014). doi:10.1098/rsos.140216

3. Button, K. S. *et al.* Power failure: Why small sample size undermines the reliability of neuroscience. *Nat. Rev. Neurosci.* (2013). doi:10.1038/nrn3475

4. Beck, T., Hastings, R. K., Gollapudi, S., Free, R. C. & Brookes, A. J. GWAS Central: A comprehensive resource for the comparison and interrogation of genome-wide association studies. *Eur. J. Hum. Genet.* (2014). doi:10.1038/ejhg.2013.274

5. Eicher, J. D. *et al.* GRASP v2.0: An update on the Genome-Wide Repository of Associations between SNPs and Phenotypes. *Nucleic Acids Res.* (2015). doi:10.1093/nar/gku1202

6. Aymé, S., Bellet, B. & Rath, A. Rare diseases in ICD11: Making rare diseases visible in health information systems through appropriate coding. *Orphanet J. Rare Dis.* (2015). doi:10.1186/s13023-015-0251-8

7. Robinson, P. N. Classification and coding of rare diseases: overview of where we stand, rationale, why it matters and what it can change. *Orphanet J. Rare Dis.* (2012). doi:10.1186/1750-1172-7-s2-a10

8. Pertea, M. & Salzberg, S. L. Between a chicken and a grape: estimating the number of human genes. *Genome Biology* (2010). doi:10.1186/gb-2010-11-s1-I1

9. Vogel F. A preliminary estimate of the number of human genes. *Nature* **201**, 847 (1964).

10. SA, K. Metabolic stability and epigenesis in randomly constructed genetic nets. *J. Theor. Biol.* **22**, 437–467 (1969).

11. Pennisi, E. HUMAN GENOME: A Low Number Wins the GeneSweep Pool. *Science (80-. ).* (2003). doi:10.1126/science.300.5625.1484b

12. Uhlén, M. *et al.* Tissue-based map of the human proteome. *Science (80-. ).* (2015). doi:10.1126/science.1260419

13. Ezkurdia, I. *et al.* Multiple evidence strands suggest that theremay be as few as 19 000 human protein-coding genes. *Hum. Mol. Genet.* (2014). doi:10.1093/hmg/ddu309

14. Fuchsberger, C. *et al.* The genetic architecture of type 2 diabetes. *Nature* (2016). doi:10.1038/nature18642

15. IBDGenetics - Home page. Available at: https://www.ibdgenetics.org/. (Accessed: 3rd July 2019)

16. Prasad, R. B. & Groop, L. Genetics of type 2 diabetes—pitfalls and possibilities. *Genes (Basel).* (2015). doi:10.3390/genes6010087

17. Deloukas, P. *et al.* Large-scale association analysis identifies new risk loci for coronary artery disease. *Nat. Genet.* (2013). doi:10.1038/ng.2480

18. Tenesa, A. & Haley, C. S. The heritability of human disease: Estimation, uses and abuses. *Nature Reviews Genetics* (2013). doi:10.1038/nrg3377

19. RA, F. The correlation between relatives on the supposition of Mendelian inheritance. *Trans. R. Soc. , Edinburgh* **52**, 399–433 (1918).

20. Boyle, E. A., Li, Y. I. & Pritchard, J. K. An Expanded View of Complex Traits: From Polygenic to Omnigenic. *Cell* (2017). doi:10.1016/j.cell.2017.05.038

21. Hopkins, A. L. & Groom, C. R. The druggable genome. *Nat. Rev. Drug Discov.* (2002). doi:10.1038/nrd892

22. Russ, A. P. & Lampel, S. The druggable genome: an update. *Drug Discov. Today* (2005). doi:10.1016/s1359-6446(05)03666-4

23. Griffith, M. *et al.* DGIdb: Mining the druggable genome. *Nat. Methods* (2013). doi:10.1038/nmeth.2689

24. Finan, C. *et al.* The druggable genome and support for target identification and validation in drug development. *Sci. Transl. Med.* **9**, (2017).

25. Santos, R. *et al.* A comprehensive map of molecular drug targets. *Nat. Rev. Drug Discov.* (2016). doi:10.1038/nrd.2016.230

26. Rask-Andersen, M., Masuram, S. & Schiöth, H. B. The Druggable Genome: Evaluation of Drug Targets in Clinical Trials Suggests Major Shifts in Molecular Class and Indication. *Annu. Rev. Pharmacol. Toxicol.* (2013). doi:10.1146/annurev-pharmtox-011613-135943

27. Beadle, G. W. & Tatum, E. L. Genetic Control of Biochemical Reactions in Neurospora. *Proc. Natl. Acad. Sci. U. S. A.* (1941).

28. Lee, Y. & Rio, D. C. Mechanisms and Regulation of Alternative Pre-mRNA Splicing. *Annu. Rev. Biochem.* (2015). doi:10.1146/annurev-biochem-060614-034316

29. Ponting, C. P. & Russell, R. R. The Natural History of Protein Domains. *Annu. Rev. Biophys. Biomol. Struct.* (2002). doi:10.1146/annurev.biophys.31.082901.134314

30. Mani, M. *et al.* MoonProt: A database for proteins that are known to moonlight. *Nucleic Acids Res.* (2015). doi:10.1093/nar/gku954

31. Sivakumaran, S. *et al.* Abundant pleiotropy in human complex diseases and traits. *Am. J. Hum. Genet.* (2011). doi:10.1016/j.ajhg.2011.10.004

32. Shi, H., Kichaev, G. & Pasaniuc, B. Contrasting the Genetic Architecture of 30 Complex Traits from Summary Association Data. *Am. J. Hum. Genet.* (2016). doi:10.1016/j.ajhg.2016.05.013

33. Pickrell, J. K. *et al.* Detection and interpretation of shared genetic influences on 42 human traits. *Nat. Genet.* (2016). doi:10.1038/ng.3570

34. Solovieff, N., Cotsapas, C., Lee, P. H., Purcell, S. M. & Smoller, J. W. Pleiotropy in complex traits: Challenges and strategies. *Nature Reviews Genetics* (2013). doi:10.1038/nrg3461

35. Swerdlow, D. I. *et al.* HMG-coenzyme A reductase inhibition, type 2 diabetes, and bodyweight: Evidence from genetic analysis and randomised trials. *Lancet* **385**, (2015).

36. Denny, J. C., Bastarache, L. & Roden, D. M. Phenome-Wide Association Studies as a Tool to Advance Precision Medicine. *Annu. Rev. Genomics Hum. Genet.* (2016). doi:10.1146/annurev-genom-090314-024956

37. Kotsovilis, S. & Andreakos, E. Therapeutic human monoclonal antibodies in inflammatory diseases. *Methods in Molecular Biology* (2014). doi:10.1007/978-1-62703-586-6_3

38. Deepak, P. & Sandborn, W. J. Ustekinumab and Anti-Interleukin-23 Agents in Crohn’s Disease. *Gastroenterology Clinics of North America* (2017). doi:10.1016/j.gtc.2017.05.013

39. Ferrara, N. & Adamis, A. P. Ten years of anti-vascular endothelial growth factor therapy. *Nature Reviews Drug Discovery* (2016). doi:10.1038/nrd.2015.17

40. Folkman, J. Angiogenesis: An organizing principle for drug discovery? *Nat. Rev. Drug Discov.* (2007). doi:10.1038/nrd2115

41. Cortes, A. *et al.* Bayesian analysis of genetic association across tree-structured routine healthcare data in the UK Biobank. *Nat. Genet.* (2017). doi:10.1038/ng.3926

42. Overington, J. P., Al-Lazikani, B. & Hopkins, A. L. How many drug targets are there? *Nat. Rev. Drug Discov.* (2006). doi:10.1038/nrd2199

43. Rask-Andersen, M., Almén, M. S. & Schiöth, H. B. Trends in the exploitation of novel drug targets. *Nat. Rev. Drug Discov.* (2011). doi:10.1038/nrd3478

44. Nelson, M. R. *et al.* The support of human genetic evidence for approved drug indications. *Nat. Genet.* (2015). doi:10.1038/ng.3314

45. Bryois, J. *et al.* Cis and Trans Effects of Human Genomic Variants on Gene Expression. *PLoS Genet.* (2014). doi:10.1371/journal.pgen.1004461

46. Melzer, D. *et al.* A genome-wide association study identifies protein quantitative trait loci (pQTLs). *PLoS Genet.* (2008). doi:10.1371/journal.pgen.1000072

47. Folkersen, L. *et al.* Mapping of 79 loci for 83 plasma protein biomarkers in cardiovascular disease. *PLoS Genet.* (2017). doi:10.1371/journal.pgen.1006706

48. Enroth, S., Johansson, Å., Enroth, S. B. & Gyllensten, U. Strong effects of genetic and lifestyle factors on biomarker variation and use of personalized cutoffs. *Nat. Commun.* (2014). doi:10.1038/ncomms5684

49. Suhre, K. *et al.* Connecting genetic risk to disease end points through the human blood plasma proteome. *Nat. Commun.* (2017). doi:10.1038/ncomms14357

50. Sun, B. B. *et al.* Genomic atlas of the human plasma proteome. *Nature* (2018). doi:10.1038/s41586-018-0175-2

51. ENCODE: Encyclopedia of DNA Elements – ENCODE. Available at: https://www.encodeproject.org/. (Accessed: 3rd July 2019)

52. Roadmap Epigenomics Project - Home. Available at: http://www.roadmapepigenomics.org/. (Accessed: 3rd July 2019)

53. Aguet, F. *et al.* Genetic effects on gene expression across human tissues. *Nature* **550**, 204–213 (2017).

54. Casas, J. P. *et al.* PLA2G7 Genotype, lipoprotein-associated phospholipase A2 activity, and coronary heart disease risk in 10 494 cases and 15 624 controls of european ancestry. *Circulation* **121**, (2010).

55. Holmes, M. V. *et al.* Novel genetic approach to investigate the role of plasma secretory phospholipase a2 (spla2)-v isoenzyme in coronary heart disease. *Circ. Cardiovasc. Genet.* **7**, (2014).

56. Sofat, R. *et al.* Separating the mechanism-based and off-target actions of cholesteryl ester transfer protein inhibitors with CETP gene polymorphisms. *Circulation* (2010). doi:10.1161/CIRCULATIONAHA.109.865444

57. Swerdlow, D. I. *et al.* HMG-coenzyme A reductase inhibition, type 2 diabetes, and bodyweight: Evidence from genetic analysis and randomised trials. *Lancet* (2015). doi:10.1016/S0140-6736(14)61183-1

58. Swerdlow, D. I. *et al.* The interleukin-6 receptor as a target for prevention of coronary heart disease: A mendelian randomisation analysis. *Lancet* **379**, (2012).

59. Würtz, P. *et al.* Metabolomic Profiling of Statin Use and Genetic Inhibition of HMG-CoA Reductase. *J. Am. Coll. Cardiol.* **67**, (2016).

60. Millwood, I. Y. *et al.* A phenome-wide association study of a lipoprotein-associated phospholipase A 2 loss-of-function variant in 90 000 Chinese adults. *Int. J. Epidemiol.* (2016). doi:10.1093/ije/dyw087

61. Investigators, T. M. I. G. C. Inactivating Mutations in NPC1L1 and Protection from Coronary Heart Disease. *N. Engl. J. Med.* (2014). doi:10.1056/NEJMoa1405386

62. Saleheen, D. *et al.* Human knockouts and phenotypic analysis in a cohort with a high rate of consanguinity. *Nature* (2017). doi:10.1038/nature22034

63. Haplotype Reference Consortium, T. *et al.* A reference panel of 64,976 haplotypes for genotype imputation. *Nat. Genet.* (2016). doi:10.1038/ng.3643

64. Koscielny, G. *et al.* Open Targets: A platform for therapeutic target identification and Validation. *Nucleic Acids Res.* (2017). doi:10.1093/nar/gkw1055

65. Plenge, R. M., Scolnick, E. M. & Altshuler, D. Validating therapeutic targets through human genetics. *Nature Reviews Drug Discovery* (2013). doi:10.1038/nrd4051

66. S., E. & G., D. S. Mendelian randomization: Can genetic epidemiology help redress the failures of observational epidemiology? *Hum. Genet.* (2008). doi:10.1007/s00439-007-0448-6

67. Plenge, R. M. Disciplined approach to drug discovery and early development. *Sci. Transl. Med.* (2016). doi:10.1126/scitranslmed.aaf2608

68. Cosman, F. *et al.* Romosozumab Treatment in Postmenopausal Women with Osteoporosis. *N. Engl. J. Med.* (2016). doi:10.1056/NEJMoa1607948

69. FDA approves new treatment for osteoporosis in postmenopausal women at high risk of fracture | FDA. Available at: https://www.fda.gov/news-events/press-announcements/fda-approves-new-treatment-osteoporosis-postmenopausal-women-high-risk-fracture. (Accessed: 3rd July 2019)

70. Paternoster, L., Tilling, K. & Davey Smith, G. Genetic epidemiology and Mendelian randomization for informing disease therapeutics: Conceptual and methodological challenges. *PLoS Genetics* (2017). doi:10.1371/journal.pgen.1006944

71. Hu, Y.-J. *et al.* Impact of Selection Bias on Estimation of Subsequent Event Risk. *Circ. Cardiovasc. Genet.* **10**, (2017).

72. Dudbridge, F. *et al.* Adjustment for index event bias in genome-wide association studies of subsequent events. *Nat. Commun.* (2019). doi:10.1038/s41467-019-09381-w
