# Supplementary material for: Improving the odds of drug development success through human genomics: modelling study
Source: Sci Rep. 2019 Dec 11;9:18911. doi: 10.1038/s41598-019-54849-w (PMC6906499; doi:10.1038/s41598-019-54849-w)
Supplement: Supplementary file 2 — Supplementary Dataset [file 41598_2019_54849_MOESM2_ESM.docx]

**Improving the odds of drug development success through human genomics – SUPPLEMENTARY DATASET**

Aroon D. Hingorani^1, 2#^ (a.hingorani@ucl.ac.uk)

Valerie Kuan^1, 2^* (v.kuan@ucl.ac.uk)

Chris Finan^1, 2^ (c.finan@ucl.ac.uk)

Felix A. Kruger^3^ (felix.kruger@benevolent.ai)

Anna Gaulton^4^ (agaulton@ebi.ac.uk)

Sandesh Chopade^1, 2^ (sandesh.chopade15@ucl.ac.uk)

Reecha Sofat^2, 5^ (r.sofat@ucl.ac.uk)

Raymond J. MacAllister^6^ (r.macallister@icloud.com)

John P. Overington^1,^ ^7^ (jpo@md.catapult.org.uk)

Harry Hemingway^2, 5^ (h.hemingway@ucl.ac.uk)

Spiros Denaxas^2, 5^ (s.denaxas@ucl.ac.uk)

David Prieto^5,9^* (d.prieto-merino@ucl.ac.uk)

Juan Pablo Casas^8^ (Juan.CasasRomero@va.gov)

^1^Institute of Cardiovascular Science, University College London, London, UK

^2^Health Data Research, UK

^3^Benevolent AI, London, UK

^4^European Molecular Biology Laboratory, European Bioinformatics Institute (EMBL-EBI), Wellcome Genome Campus, Cambridge, UK

^5^Institute of Health Informatics, University College London, London, UK

^6^Dorset County Hospital NHS Foundation Trust, Dorchester, UK

^7^Medicines Discovery Catapult, Mereside, Alderley Park, Alderley Edge, Cheshire, UK

^8^ Massachusetts Veterans Epidemiology Research and Information Center (MAVERIC), Veterans Administration, Boston MA, USA

^9^Applied Statistics in Medical Research Group, Catholic University of Murcia (UCAM), Spain

^#^Corresponding author email: [a.hingorani@ucl.ac.uk](mailto:a.hingorani@ucl.ac.uk)

*Contributed equally

**Contents**

**Supplementary information**

Tabular comparison of the findings from orthodox randomised controlled trials or meta-analyses, and Mendelian randomisation trials of the corresponding therapeutic target.

**Supplementary information - references**

**Supplementary information**

Tabular comparison of the findings from orthodox randomised controlled trials or meta-analyses, and Mendelian randomisation trials of the corresponding therapeutic target.

| **Orthodox drug development** | | | | | | **Mendelian randomisation trials (MRT)** | | | |
| --- | --- | --- | --- | --- | --- | --- | --- | --- | --- |
| **Drug target** | **Compound(s) evaluated** | **Developmental stage** | **Therapeutic area** | **Outcomes assessed** in **preclinical studies or RCTs of selective drug interventions** | **Findings from preclinical studies or RCTs of selective drug interventions** | **Encoding gene** | **Outcomes evaluated in MRTs** | **Findings from MRTs** | **Inferences drawn from comparison of the**  **findings from preclinical studies or RCTs and MRT** |
| **Cholesteryl ester transfer protein**[1] | Torcetrapib | Phase III | Cardiovascular disease | Blood lipids (total-, LDL-, and HDL cholesterol, triglycerides); blood pressure;  CVD events | HDL-elevation, triglyceride and LDL- reduction.  Unintended BP elevation.  Unintended increase in CVD events | ***CETP***[2] | Blood lipids (total-, LDL-, and HDL cholesterol, triglycerides);  blood pressure | Associations with blood lipids consistent with effects in RCTs. No genetic association with BP. | Blood pressure elevating effect of torcetrapib is off­target |
| **Hydroxy**  **methyl (HMG)-coA reductase**[3] | Statins | Phase IV  (post-  marketing) | Cardiovascular disease | Blood lipid fractions, weight, type 2 diabetes risk | Statin treatment in RCTs linked to  increased weight and risk of type 2 diabetes. | ***HMGCR***[3] | Blood lipid fractions, anthropometric measures, glucose and insulin, type 2 diabetes risk | *HMGCR* SNPs associated with lower LDL-C,  higher weight, fasting glucose and insulin, and type 2 diabetes risk | Increased risk of type 2 diabetes is an unintended on-target effect of statins mediated in part through weight gain |
| **Niemann-Pick C1-like 1** [4] | Ezetimibe | Phase III | Cardiovascular disease | LDL-cholesterol, cardiovascular death, non-fata myocardial infarction, unstable angina requiring hospitalisation and revascularisation | Ezetimibe added to statins produces modest additional benefit in cardiovascular outcomes in patients following an acute coronary syndrome | ***NPC1L1***  [5] | Plasma lipid levels and risk of coronary heart disease. | Inactivating mutations in NPC1L1 are associated with lower LDL-cholesterol and protection from myocardial infarction risk. | Niemann-Pick C1-like 1 is a validated target for LDL-cholesterol lowering and coronary heart disease prevention. |
| **Proprotein convertase subtilisin/kexin type 9 serine protease** [6] | Alirocumab, evolocumab | Phase II | Lipid lowering and cardiovascular disease | LDL-cholesterol | Alirocumab and evolocumab reduce LDL-cholesterol among patients with heterozygous familial or polygenic hypercholesterolaemiaand reduce cardiovascular events in patients with or at high risk of cardiovascular disease | ***PCSK9***  [7] | LDL-cholesterol and rosk of coronary heart disease | Inactivating mutations in PCSK9 associated with reduced LDL-cholesterol and CHD risk | Proprotein convertase subtilisin/kexin type 9 serine protease is a validated target for LDL-cholesterol lowering and reduction in cardiovascular risk |
| **Glucagon-like peptide-1 receptor** [8] | Liraglutide | Phase III | Diabetes and cardiovascular disease | Death from cardiovascular causes, non-fata myocardial infarction, or non-fata stroke. | Liraglutide reduced risk of death from cardiovascular causes, nonfatal myocardial infarction, or nonfatal stroke among patients with type 2 diabetes mellitus | ***GLP1R***  [9] | Body weight, glycaemic traits, lipids, blood pressure, risk of type 2 diabetes and coronary heart disease | A low frequency, coding region missense variant in GLP1R is associated with lower fasting glucose, diabetes risk and risk of coronary heart disease. | GLP1R is a validated target for treatment of diabetes and reducing coronary heart disease risk |
| **Drug target** | **Compound(s) evaluated** | **Developmental stage** | **Therapeutic area** | **Outcomes assessed** in **preclinical studies or RCTs of selective drug interventions** | **Findings from preclinical studies or RCTs of selective drug interventions** | **Encoding gene** | **Outcomes evaluated in MRTs** | **Findings from MRTs** | **Inferences drawn from comparison of the**  **findings from preclinical studies or RCTs and MRT** |
| **Lipoprotein- associated phospholipase A2 (Lp-**  **PLA2)** [10,11] | Darapladib | Phase III | Cardiovascular disease | Major cardiovascular events or major coronary events | No reduction in CVD events in patients with stable coronary disease or recent ACS; despite reductions in Lp-PLA2 mass and activity. | ***PLA2G7***[12, 13] | Lp-PLA2  concentration, blood lipids, inflammation markers, and CHD events | PLA2G7 variants were not associated with alterations in cardiovascular risk markers or CHD events | Lp-PLA2 is not involved in the development of cardiovascular disease; low priority as therapeutic target for this indication |
| **Interleukin-6 receptor**[14] | Tocilizumab | Phase III | Autoimmune disease | Blood lipid fractions and inflammation markers including IL- 6, CRP and fibrinogen | In patients with rheumatoid arthritis, tocilizumab induced alterations in  circulating  inflammation markers characteristic of IL-6 blockade | ***IL6R***[14] | Blood lipid fractions and inflammation markers including iL-6, CRP and fibrinogen. Cardiovascular events including CHD events and abdominal aortic aneurysm | Variants in the *IL6R*  gene that recapitulate the biomarker profile of IL6-R blockade were associated with a reduction in CHD events | IL-6 receptor signalling is involved in the  development of CHD. The IL-6 receptor blocker tocilizumab could be repurposed for the treatment of CVD |
| **C-reactive protein**[15] | No CRP inhibitors yet available for clinical use. | Preclinical | Cardiovascular disease | Effects of CRP on processes believed to contribute to atherosclerosis  studied *in vitro* or in animals*.*  Associations of CRP with CVD in human observational  studies. | Observational associations of CRP with CVD events in humans, but studies prone to confounding. Pro-atherogenic  effect of CRP *in vitro* and in animals later proved to be artefactual. | ***CRP***[16] | Inflammation and coagulation markers, blood lipid fractions, and coronary heart disease events | SNPs in the CRP gene exclusively associated with CRP exhibited no association with CHD. No causal association of CRP with CHD based on instrumental variables analysis. | CRP is not  Causal in CHD pathogenesis; priority as a therapeutic target for CHD prevention diminished |
| **Secretory phospholipase A2 (sPLA2)**[17] | Varespladib | Phase III | Cardiovascular disease | sPLA2 concentration, blood lipids, inflammation  markers, and CVD events | No beneficial effect of varespladib on CVD events in patients with recent acute coronary syndrome (ACS), despite a drug- induced reduction in sPLA2 concentration and activity | ***PLA2G2A***[18] | sPLA2 mass and  activity and major vascular events (MVE) in general populations and patients with ACS | SNPs in the PLA2G2A gene were associated with substantial alterations in sPLA2 mass and activity but not with MVE | sPLA2 is not involved in the development of cardiovascular disease; dismissed as a therapeutic target in CVD |
| **Potassium/sodium hyperpolarization-activated cyclic nucleotide-gated channel 4** [19] | Ivabradine | Phase IV  (post-  marketing) | Cardiovascular disease | Risk of atrial fibrillation | Developed for angina and heart failure, post-hoc meta-analysis of RCTs (motivated by genetic findings [14, 15], indicated ivabridine treatment is associated with a higher risk of atrial fibrillation. | ***HCN4*** [20,21] | Atrial fibrillation (genome wide association analysis) | Variants in the gene *HCN4* encoding the target of ivabridine associate with a higher risk of atrial fibrillation. | Atrial fibrillation is a mechanism-based adverse effect of ivabridine treatment. |
| **Drug target** | **Compound(s) evaluated** | **Developmental stage** | **Therapeutic area** | **Outcomes assessed** in **preclinical studies or RCTs of selective drug interventions** | **Findings from preclinical studies or RCTs of selective drug interventions** | **Encoding gene** | **Outcomes evaluated in MRTs** | **Findings from MRTs** | **Inferences drawn from comparison of the**  **findings from preclinical studies or RCTs and MRT** |
| **TNF receptor 1 and TNF** [22 23] | Monoclonal antibodies against tumour necrosis factor-alpha (TNF) | Phase II I and Phase IV | Neurological disease | Multiple sclerosis exacerbations | Multiple sclerosis exacerbations. | ***TNFRSF1A***  [24] | Multiple sclerosis | A variant in the TNFRSF1A that encodes the TNF receptor 1 gene indices expression of a soluble form of TNFR1 that blocks the effect of TNF, and associates with a higher risk of MS. The mechanism mimics that of monoclonal antibodies against TNF. | Exacerbation of MS induced by anti-TNF monoclonal antibodies is mechanism based. |

**Supplementary information references**

1. Sofat R, Hingorani AD, Smeeth L, Humphries SE, Talmud PJ, Cooper J, et al. Separating the Mechanism-Based and Off-Target Actions of Cholesteryl Ester Transfer Protein Inhibitors With CETP Gene Polymorphisms. Circulation. 2010;121: 52–62. doi:10.1161/CIRCULATIONAHA.109.865444
2. Barter PJ, Caulfield M, Eriksson M, Grundy SM, Kastelein JJP, Komajda M, et al. Effects of Torcetrapib in Patients at High Risk for Coronary Events. N Engl J Med. 2007;357: 2109–2122. doi:10.1056/NEJMoa0706628
3. Swerdlow DI, Preiss D, Kuchenbaecker KB, Holmes MV, Engmann JEL, Shah T, et al. HMG-coenzyme A reductase inhibition, type 2 diabetes, and bodyweight: evidence from genetic analysis and randomised trials. The Lancet. 2014;385: 351–361. doi:10.1016/S0140-6736(14)61183-1
4. Cannon CP, Blazing MA, Giugliano RP, McCagg A, White JA, Theroux P, Darius H, Lewis BS, Ophuis TO, Jukema JW, De Ferrari GM, Ruzyllo W, De Lucca P, Im K, Bohula EA, Reist C, Wiviott SD, Tershakovec AM, Musliner TA, Braunwald E, Califf RM; IMPROVE-IT Investigators.. N Engl J Med. 2015 Jun 18;372(25):2387-97
5. The Myocardial Infarction Genetics Consortium Investigators Inactivating mutations in NPC1L1 and protection from coronary heart disease. N Engl J Med 2014; 371:2072-2082
6. Schmidt AF, Pearce LS, Wilkins JT, Overington JP, Hingorani AD, Casas JP PCSK9 monoclonal antibodies for the primary and secondary prevention of cardiovascular disease. Cochrane Database Syst Rev. 2017 Apr 28;4:CD011748. doi: 10.1002/14651858.CD011748.pub2
7. Cohen JC, Boerwinkle E, Mosley TH, Hobbs HH. Sequence variations in PCSK9, low LDL, and protection against coronary heart disease. N Engl J Med. 2006;354:1264–72
8. Marso SP, Daniels GH, Brown-Frandsen K et al. for the LEADER Steering Committee on behalf of the LEADER Trial Investigators Liraglutide and Cardiovascular Outcomes in Type 2 Diabetes N Engl J Med 2016; 375:311-322
9. Scott RA, Freitag DF, Li L, et al. Genomic approach to therapeutic target validation identifies a glucose-lowering *GLP1R* variant protective for coronary heart disease Sci Transl Med. 2016 Jun 1; 8(341): 341ra76. doi: 10.1126/scitranslmed.aad3744
10. Darapladib for Preventing Ischemic Events in Stable Coronary Heart Disease. N Engl J Med. 2014;370: 1702–1711. doi:10.1056/NEJMoa1315878
11. O’Donoghue ML, Braunwald E, White HD, et al. Effect of darapladib on major coronary events after an acute coronary syndrome: The SOLID-TIMI-52 randomized clinical trial. JAMA. 2014;312: 1006–1015. doi:10.1001/jama.2014.11061
12. Casas JP, Ninio E, Panayiotou A, Palmen J, Cooper JA, Ricketts SL, et al. PLA2G7 Genotype, Lipoprotein-Associated Phospholipase A2 Activity, and Coronary Heart Disease Risk in 10 494 Cases and 15 624 Controls of European Ancestry. Circulation. 2010;121: 2284–2293. doi:10.1161/CIRCULATIONAHA.109.923383
13. Millwood IY, Bennett DA, Walters RG, Clarke R, Waterworth D, Johnson T, Chen Y, Yang L, Guo Y, Bian Z, Hacker A, Yeo A, Parish S, Hill MR, Chissoe S, Peto R, Cardon L, **Collins R**, Li L, **Chen Z**; China Kadoorie Biobank Collaborative Group. [Lipoprotein-Associated Phospholipase A2 Loss-of-Function Variant and Risk of Vascular Diseases in 90,000 Chinese Adults.](https://www.ncbi.nlm.nih.gov/pubmed/26791069) J Am Coll Cardiol. 2016 Jan 19;67(2):230-1
14. The interleukin-6 receptor as a target for prevention of coronary heart disease: a mendelian randomisation analysis. The Lancet. 2012;379: 1214–1224. doi:10.1016/S0140-6736(12)60110-X
15. Casas JP, Shah T, Hingorani AD, Danesh J, Pepys MB. C-reactive protein and coronary heart disease: a critical review. J Intern Med. 2008;264: 295–314. doi:10.1111/j.1365-2796.2008.02015.x
16. C Reactive Protein Coronary Heart Disease Genetics Collaboration (CCGC). Association between C reactive protein and coronary heart disease: mendelian randomisation analysis based on individual participant data. BMJ. 2011;342: d548–d548. doi:10.1136/bmj.d548
17. Nicholls SJ, Kastelein JP, Schwartz GG, et al. Varespladib and cardiovascular events in patients with an acute coronary syndrome: The VISTA-16 randomized clinical trial. JAMA. 2014;311: 252–262. doi:10.1001/jama.2013.282836
18. Holmes MV, Simon T, Exeter HJ, Folkersen L, Asselbergs FW, Guardiola M, et al. Secretory phospholipase A(2)-IIA and cardiovascular disease: a mendelian randomization study. J Am Coll Cardiol. 2013;62: 1966–76. doi:10.1016/j.jacc.2013.06.044
19. Martin RIR, Pogoryelova O, Koref MS, Bourke JP, Teare MD, Keavney BD. Atrial fibrillation associated with ivabradine treatment: meta-analysis of randomised controlled trials. Heart. 2014 Oct 1; 100(19): 1506–1510.
20. Ellinor PT, Lunetta KL, Albert CM, et al. Meta-analysis identifies six new susceptibility loci for atrial fibrillation. Nat Genet 2012;44:670–5
21. den Hoed M, Eijgelsheim M, Esko T, et al. Identification of heart rate-associated loci and their effects on cardiac conduction and rhythm disorders. Nat Genet 2013;45:621–31
22. van Oosten BW, et al. Increased MRI activity and immune activation in two multiple sclerosis patients treated with the monoclonal anti-tumor necrosis factor antibody cA2. Neurology. 1996;47:1531–1534.
23. The Lenercept Multiple Sclerosis Study Group. The University of British Columbia MS/MRI Analysis Group TNF neutralization in MS: results of a randomized, placebo-controlled multicenter study. Neurology. 1999;53:457–465
24. Gregory AP, Dendrou CA., Attfield KE et al. TNF receptor 1 genetic risk mirrors outcome of anti-TNF therapy in multiple sclerosis *Nature* 2012; 488: 508–511
